# Effective Decolorization and Detoxification of Single and Mixed Dyes with Crude Laccase Preparation from a White-Rot Fungus Strain *Pleurotus eryngii*

**DOI:** 10.3390/molecules29030669

**Published:** 2024-01-31

**Authors:** Mingrui Ge, Wei Deng, Ziyi Wang, Chenwen Weng, Yang Yang

**Affiliations:** School of Life Sciences, Hubei Key Laboratory of Genetic Regulation and Integrative Biology, Central China Normal University, Wuhan 430079, China

**Keywords:** white-rot fungi, laccase, dyes, dye mixtures, decolorization, repeated-batch decolorization, detoxification

## Abstract

To fully harness the potential of laccase in the efficient decolorization and detoxification of single and mixed dyes with diverse chemical structures, we carried out a systematic study on the decolorization and detoxification of single and mixed dyes using a crude laccase preparation obtained from a white-rot fungus strain, *Pleurotus eryngii*. The crude laccase preparation showed efficient decolorization of azo, anthraquinone, triphenylmethane, and indigo dyes, and the reaction rate constants followed the order Remazol Brilliant Blue R > Bromophenol blue > Indigo carmine > New Coccine > Reactive Blue 4 > Reactive Black 5 > Acid Orange 7 > Methyl green. This laccase preparation exhibited notable tolerance to SO_4_^2−^ salts such as MnSO_4_, MgSO_4_, ZnSO_4_, Na_2_SO_4_, K_2_SO_4_, and CdSO_4_ during the decolorization of various types of dyes, but was significantly inhibited by Cl^−^ salts. Additionally, this laccase preparation demonstrated strong tolerance to some organic solvents such as glycerol, ethylene glycol, propanediol, and butanediol. The crude laccase preparation demonstrated the efficient decolorization of dye mixtures, including azo + azo, azo + anthraquinone, azo + triphenylmethane, anthraquinone + indigo, anthraquinone + triphenylmethane, and indigo + triphenylmethane dyes. The decolorization kinetics of mixed dyes provided preliminary insight into the interactions between dyes in the decolorization process of mixed dyes, and the underlying reasons and mechanisms were discussed. Importantly, the crude laccase from *Pleurotus eryngii* showed efficient repeated-batch decolorization of single-, two-, and four-dye mixtures. This crude laccase demonstrated high stability and reusability in repeated-batch decolorization. Furthermore, this crude laccase was efficient in the detoxification of different types of single dyes and mixed dyes containing different types of dyes, and the phytotoxicity of decolorized dyes (single and mixed dyes) was significantly reduced. The crude laccase efficiently eliminated phytotoxicity associated with single and mixed dyes. Consequently, the crude laccase from *Pleurotus eryngii* offers significant potential for practical applications in the efficient decolorization and management of single and mixed dye pollutants with different chemical structures.

## 1. Introduction

Laccase is one of the important ligninolytic enzymes produced by white-rot fungi. Laccase, known for its ability to catalyze the oxidation of phenols, polyphenols, anilines, lignin, and polycyclic aromatic hydrocarbons, consists of a copper-containing polyphenol oxidase. This enzymatic process occurs in the presence of molecular oxygen, resulting in the formation of corresponding free radicals and the reduction of molecular oxygen to water. The catalytic center of laccase contains four copper atoms, which can be classified into three types based on their spectral and magnetic properties, namely, type I copper (T1), type II copper (T2), and type III copper (T3). T1 is located in the substrate-binding site, with T2 proximal to T3, forming a trinuclear copper center essential for O_2_ reduction [1,2]. The laccase-catalyzed oxidation reaction occurs in three steps. First, T1 extracts an electron from the reduced substrate, resulting in oxidization to a free radical, and the redox potential of T1 must exceed that of the substrate for this reaction to occur. Second, through the Cys-His bridge, the electron will be transferred from T1 to the trinuclear copper center, causing sequential reduction of T3 and T2. Finally, within the catalytic center, molecular oxygen will undergo reduction into water, mediated by superoxide formation. Water consists of the sole byproduct of laccase-catalyzed oxidation [3]. Laccase has broad application in the efficient degradation of various organic pollutants in the environment due to its outstanding characteristics, such as a broad substrate spectrum, molecular oxygen as the terminal electron acceptor, unique oxidation mechanism without the need for cofactors or peroxides, mild reaction conditions, and environmental friendliness [4,5]. Extensive research has shown that laccase can effectively degrade a variety of recalcitrant environmental pollutants, including industrial synthetic dyes [6,7], olefins [8,9], chlorophenols [10,11], polycyclic aromatic hydrocarbons [12,13], pesticides [14,15], and endocrine disruptors [16,17]. As a result, the ability of laccase to degrade various environmental pollutants has received increasing attention.

Dyes consist of a class of organic compounds known for imparting bright and durable colors to various materials. With the rapid development of modern industry, synthetic dyes have found application in photography, textile, papermaking, pharmacy, food, and cosmetics. Due to their extensive color range, color brightness, exceptional functionality, and broad suitability, dyes can serve the market demands for fibers and clothing materials. As a result, the textile industry has emerged as the primary consumer of synthetic dyes. Commercially, azo, triphenylmethane, and anthraquinone dyes are widely used in the textile sector [18,19]. Synthetic dyes can be categorized into five groups based on their chromophores, namely, azo, anthraquinone, indigo, triphenylmethane, and phthalocyanine dyes. Of these, azo, anthraquinone, and triphenylmethane are the most commonly used commercial dyes, with azo dyes constituting approximately 70% of all dyes used in industrial processes [20]. Anthraquinone dyes represent the second largest category of textile dyes due to their excellent colorfastness, lightfastness, and a diverse color spectrum. These dyes have typically been employed in the dyeing of cellulose-based fabrics, wool, and polyamide fibers [21]. Although dyes have found widespread application in industries, their extensive use and consequential discharge have led to significant environmental contamination, resulting in adverse impacts on both ecological ecosystems and human health [22]. During the dyeing process, approximately 10% to 15% of dyes are discharged into wastewater, causing serious environmental and health problems. Dyes at relatively low concentrations can significantly increase the chromaticity of water bodies, diminishing water transparency and self-purification. The presence of dyes in aquatic ecosystems will hinder light penetration into deeper layers, reducing photosynthetic processes in aquatic organisms, deteriorating the water quality, and posing a direct threat to the survival of aquatic organisms [23,24]. The discharge of dye-contaminated wastewater into environments such as lakes and rivers can cause severe damage to aquatic organisms, potentially inducing mutagenic and teratogenic effects [24]. Other than their ecological implications, dye pollutants can also pose a significant threat to human health. Azo, anthraquinone, and triphenylmethane dyes have carcinogenic properties, manifesting through direct contact with the human body and through the production of carcinogenic compounds as a result of dye degradation [25]. One carcinogenic degradation product of azo dyes is *p*-diaminobiphenyl. Within its molecular structure, -N=N- can undergo reduction by a reducing agent, generating aromatic amines in humans or animals, contributing to the development of liver, bladder, breast, and colon cancers [26]. Moreover, specific active groups in azo dyes can covalently bind to proteins or amino acids in skin and induce skin allergies, and certain substituents in anthraquinone dyes may also trigger skin sensitization in humans [27]. Some anthraquinone-based disperse dyes also contain one or more primary amines capable of inserting into the base pairs of the DNA helix, thus, posing a carcinogenic risk [28]. Several triphenylmethane dyes also have carcinogenic properties and can be highly irritating to the eyes, with various synthetic dyes, including acid dyes, basic dyes, and direct dyes, exhibiting carcinogenic effects. Reactive dyes such as reactive red and reactive blue have been associated with occupational asthma, while disperse dyes can lead to dermatological allergies [29]. Overall, the presence of dye pollutants represents a significant risk to the ecological environment and human health, making it imperative to adopt effective methods for the degradation of dye pollutants and mitigation of their toxic effects. Hence, the exploration of dye pollutant degradation and detoxification has scientific importance and practical implications for eliminating or mitigating the considerable damage caused to the ecosystem and human health.

Recent research has revealed that crude laccase preparations and purified laccases hold substantial promise for the efficient degradation of dyes with diverse structures [30]. Azo dyes contain -N=N- in their molecules and represent the most intensively and widely used synthetic dyes (>70%). Zeng et al. utilized *Trametes trogii* crude laccase preparation for the decolorization of azo dyes Acid Red 1 and Reactive Black 5, demonstrating significant enhancement in decolorization with a 1 mM redox mediator (1-hydroxybenzotriazole) [31]. Pan et al. employed a crude laccase preparation from *Phanerochaete chrysosporium* for the decolorization and degradation of the azo dye Reactive Turquoise Blue KGL, achieving a decolorization efficiency of up to 80% under optimal conditions within 20 min [32]. Yanto et al. explored the degradation of various dyes using crude laccase preparation derived from *Trametes hirsuta*. The study demonstrated variations in the decolorization efficacy associated with the chemical structures of dyes, and the efficient decolorization of the azo dye Reactive Black 5 was achieved with violuric acid as a mediator [33]. Couto et al. assessed the performance of crude laccase from *T*. *hirsuta* in the decolorization of various industrial azo dyes, demonstrating that the azo dye SSB 4GL exhibited a high level of decolorization within 4 h (73%), followed by DC NBS (67%). However, the azo dyes DP 5GL and SSY 4GL displayed a high resistance to decolorization, potentially due to the nature and position of the dye substituents [34]. Jasińska et al. studied the degradation of azo dyes using a crude laccase preparation from *Myrothecium roridum*. The introduction of a synthetic or natural mediator to a reaction mixture containing 1 U/mL laccase and the azo dye AB 113 resulted in a significant enhancement of the decolorization efficiency, indicating that appropriate mediator supplementation could promote the degradation of substrates that may be less amenable to laccase-mediated degradation [35]. Anthraquinone dyes contain an anthraquinone unit in their molecules, including anthraquinone derivatives and fused-ring ketone dyes produced from anthraquinone derivatives. Anthraquinone dyes have found extensive application in the dyeing and textile industries, primarily due to their vibrant colors, high fixation rate, and excellent colorfastness. Zeng et al. observed that a crude laccase preparation of *T*. *trogii* provided efficient decolorization of the anthraquinone dyes Remazol Brilliant Blue R (RBBR), Reactive Blue 4 (RB4), and Acid Blue 129 without a mediator [36]. Hou et al. highlighted the efficient decolorization of anthraquinone dyes through a crude laccase preparation derived from *Pleurotus ostreatus*, with the addition of an appropriate concentration of the redox mediator 2,2′-azino-bis(3-ethylbenzothiazoline-6-sulfonate) (ABTS), which further enhanced the degradation efficiency [37]. Wong et al. found that anthraquinone dyes served as substrates for laccase-mediated first-order reactions, and anthraquinone dyes, as excellent substrates of laccase, showed increased decolonization with increasing enzyme activity [38]. Yadav et al. reported on the efficient decolorization of the anthraquinone dye RBBR with laccase from *Arthrographis kalrae*, even in the absence of a redox mediator. Decolorization was influenced by parameters such as the enzyme dosage, pH, treatment duration, and initial dye concentration [39]. Osma et al. investigated the decolorization of RBBR using a crude laccase prepared from *Trametes pubescens*. The study found that the formation of enzyme inhibitors during the decolorization process led to a significant reduction in the decolorization rate after a certain period [40]. Following azo and anthraquinone dyes, triphenylmethane dyes represent the third-most commonly used synthetic dyes in the industry. These dyes feature a distinctive molecular structure with three benzene rings attached to a carbon atom, with each carrying different side chains. The inherent stability of this chemical structure results in resistance to degradation by common microorganisms. Consequently, triphenylmethane dyes with high toxicity and bio-degradation resistance may cause serious environmental pollution when discharged into wastewater. Grassi et al. employed a crude laccase preparation from *T*. *trogii* to degrade the triphenylmethane dyes malachite green, bromophenol blue, and crystal violet, indicating that the decolorization efficiency was higher than 85% within one day [41]. Li et al. utilized a crude laccase preparation from *Pycnoporus sanguineus* to decolorize crystal violet. However, the study noted that crystal violet was not a direct substrate and required the addition of the mediator ABTS to facilitate decolorization, resulting in a moderate decolorization efficiency of 35% within 180 min [42]. Hadibarata et al. reported that crude laccase prepared from *Armillaria* sp. F022 displayed a limited degradation of the triphenylmethane dye brilliant green, and the degradation efficiency was less than 40% after 96 h of treatment [43]. In addition, Núria et al. used *T*. *versicolor* laccase to decolorize the triphenylmethane dyes brilliant green 1, methyl green (MG), and acid fuchsin (AF). Brilliant green 1 and MG were completely decolorized within 24 h, and only 28% of AF was decolorized within 48 h [44]. These variations were attributed to the differences in chemical structures of the triphenylmethane dyes. The degradation of triphenylmethane dyes with laccase was influenced by the dye structures, likely due to variations in the redox potential of laccases from different sources. Indigo dyes contain a C=C bond connecting two heterocyclic rings with carbonyl groups, forming a chromophore comprised of a conjugated system with four atoms in the carbonyl-double bonded-carbonyl groups. Indigo dyes are primarily used in denim production, and the substantial consumption of denim has resulted in the production of substantial amounts of indigo-containing wastewater. Indigo dyes consist of aromatic reducing dyes with a stable conjugated structure and poor biodegradability, posing significant environmental risks. Given the structural stability of indigo dyes, small-molecule electron-transfer mediators with diverse structures have been incorporated to facilitate their oxidation and degradation. Hu et al. utilized 1-hydroxybenzotriazole as a mediator for indigo dye decolorization with a crude laccase prepared from *T*. *versicolor*, achieving up to 90.1% decolorization efficiency within 80 min. The study showed nearly complete decolorization (90–100%) of IC within 1 h using phloroglucinol, thymol, and violuric acids as laccase mediators [45].

Prior research on laccase-mediated dye degradation has focused on assessing the decolorization of a specific dye with laccase from a specific source, with limited information on the degradation of a broad spectrum of dyes containing various structures. Furthermore, industrial dye wastewater typically consists of a complex mixture containing various dyes. Therefore, the investigation of laccase-mediated decolorization of dye mixtures holds substantial practical and theoretical value for the efficient treatment of industrial dye wastewater. Past studies, however, have focused on single-dye degradation with laccase, with little exploration of multi-dye degradation, and interaction between various dyes in the laccase-mediated degradation of dye mixtures has remained largely unexplored. The ability of laccase to detoxify dye pollutants during decolorization remains critical for evaluating its effectiveness in treating dye pollutants. However, previous research has mostly been limited to the detoxification effect of laccase on a single dye, and there is still a lack of reports on the detoxification ability of laccase on mixed dyes (mixtures of different types of dyes). There are almost no reports on the detoxification effect of laccase on different types of dye mixtures. Industrial dye wastewater often contains high levels of metal ions and organic solvents, which may influence laccase activity and, consequently, its effectiveness in treating dye wastewater. As a result, the comprehensive examination of laccase tolerance to various metal ions and organic solvents during dye decolorization holds practical value for the efficient treatment of dye wastewater with abundant metal ions and organic solvents. Nevertheless, systematic and in-depth research in this area remains limited.

In this study, we addressed the unresolved issues outlined above and obtained a laccase resource with enhanced applicability for the efficient decolorization and detoxification of both single and mixed dyes. To explore and utilize the potential of laccase in the efficient degradation and detoxification of single and mixed dyes, a crude laccase was prepared from the white-rot fungus strain *Pleurotus eryngii*. Subsequently, we systematically assessed the decolorization efficacy and reaction rates of single azo, anthraquinone, triphenylmethane, and indigo dyes. We also investigated the degradation kinetics and tolerance of this enzyme to varying concentrations of metal salts and organic solvents during the decolorization of four types of dyes. We evaluated the potential of this crude laccase for the treatment of dye wastewater containing diverse salts and organic solvents. Besides the single dyes, we also systematically examined the decolorization of dye mixtures and revealed their decolorization kinetics (azo + azo, azo + anthraquinone, azo + triphenylmethane, azo + indigo, anthraquinone + indigo, anthraquinone + triphenylmethane, and indigo + triphenylmethane). Accordingly, we systematically investigated the repeated-batch decolorization of single dyes and dye mixtures containing two and four dyes. The efficiency of repeated-batch decolorization of both single and mixed dyes was demonstrated, and we evaluated the reusability, stability, and efficiency of the crude laccase in repeated-batch decolorization. Finally, we assessed the elimination of phytotoxicity through the crude laccase for single and mixed dyes. Our assessment involved analyzing the impact of these dyes (single and mixed dyes) on the growth of crop seedlings before and after laccase-mediated decolorization. Our findings revealed that the crude laccase from *Pleurotus eryngii* displayed distinct advantages and properties. These included the efficient decolorization of a wide range of dyes, high tolerance against salts and organic solvents, stable performance in repeated-batch decolorization of both single and mixed dyes, and simultaneous decolorization and detoxification for single dyes and mixtures of different types of dyes (mixed dyes). Therefore, the crude laccase obtained from this study offers significant potential in the efficient degradation and treatment of pollutants containing single or mixed dyes.

## 2. Results

### 2.1. Preparation of the Crude Laccase from Pleurotus eryngii and Determination of Laccase Activity

**Figure 1:**
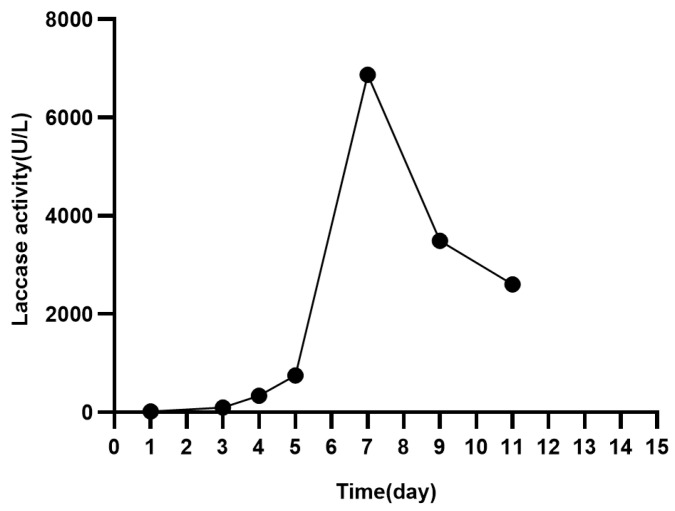
The production of laccase by *Pleurotus eryngii* after the addition of 2 mM CuSO_4_ as the inducer. A 2 mM solution of CuSO_4_ was added to the fungal culture on the third day.

The mycelia of *Pleurotus eryngii* were transferred from the PDB medium to GYP medium for laccase production. On day 3 of culturing in the GYP medium, CuSO_4_ was added to induce laccase production. As shown in Figure 1, the laccase activity significantly increased after CuSO_4_ addition and reached a maximum of 6868.33 U/L on day 7. Laccase activity was determined as described in the literature [46]. The crude laccase preparation was stored at −20 °C for later use.

### 2.2. Decolorization of Azo Dyes

#### 2.2.1. Decolorization Efficiency of Azo Dyes at Different Concentrations

**Figure 2:**
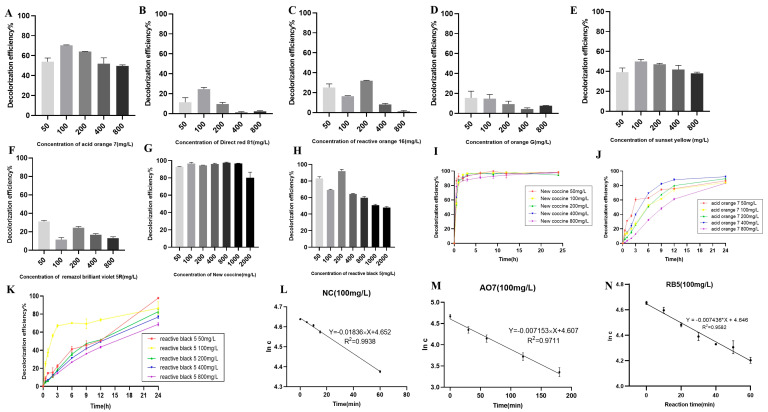
Decolorization of eight azo dyes with the crude laccase from *P. eryngii*. (**A**) Decolorization efficiencies of Acid Orange 7 at different concentrations. (**B**) Decolorization efficiencies of Direct Red 81 at different concentrations. (**C**) Decolorization efficiencies of Reactive Orange 16 at different concentrations. (**D**) Decolorization efficiencies of Orange G at different concentrations. (**E**) Decolorization efficiencies of Sunset Yellow at different concentrations. (**F**) Decolorization efficiencies of Remazol Brilliant violet 5R at different concentrations. (**G**) Decolorization efficiencies of New Coccine at different concentrations. (**H**) Decolorization efficiencies of Reactive Black 5 at different concentrations. (**I**) Time course of decolorization of New Coccine (final concentration was 50, 100, 200, 400, 800, 1000, 2000 mg/L). (**J**) Time course of decolorization of Acid Orange 7 (final concentration was 50, 100, 200, 400, 800 mg/L). (**K**) Time course of decolorization of Reactive Black 5 (final concentration was 50, 100, 200, 400, 800 mg/L). (**L**) The ln c-t curve of decolorization of the azo dye NC (100 mg/L). (**M**) The ln c-t curve of decolorization of the azo dye AO7 (100 mg/L). (**N**) The ln c-t curve of decolorization of the azo dye RB5 (100 mg/L). NC: New Coccine, AO7: Acid Orange 7, RB5: Reactive Black 5.

The crude laccase preparation showed excellent decolorization of NC at different concentrations. The 24 h decolorization efficiencies were 92.97%, 96.59%, and 94.48% for 50, 100, and 200 mg/L of NC, respectively, indicating that the 24 h decolorization efficiency remained high with increased NC concentrations at 400 (96.12%) and 800 mg/L (97.61%) (Figure 2G).

As shown in Figure 2A,E,H, the crude laccase preparation demonstrated efficient decolorization of AO7, SY, and RB5. The 24 h decolorization efficiencies were 72.58%, 70.34%, and 47.22% for 100 mg/L of RB5, AO7, and SY, respectively. The 24 h decolorization efficiencies were 59.81%, 49.71%, and 38.11% for 800 mg/L of RB5, AO7, and SY, respectively, indicating that laccase remained active with increased dye concentration.

The crude laccase preparation demonstrated moderate decolorization of DR81, RO16, OG, and RV5R (Figure 2B–D,F). The 24 h decolorization efficiency of these dyes at different concentrations remained below 30%. The 24 h decolorization efficiencies were 24.61%, 16.47%, 14.81%, and 11.57% for 100 mg/L DR81, RO16, OG, and RV5R, respectively.

In summary, the decolorization efficiency of eight azo dyes followed the order NC > RB5 > AO7 > SY > DR81 > RO16 > OG > RV5R.

#### 2.2.2. Time Courses of Decolorization of Azo Dyes at Different Concentrations

The time courses of decolorization were plotted for NC, AO7, and RB5 at 50, 100, 200, 400, and 800 mg/L. As shown in Figure 2I, the crude laccase preparation showed very rapid decolorization of 100 mg/L NC, with decolorization efficiencies of 83.95%, 98.01%, and 99.42% after 1, 2, and 3 h, respectively. The crude laccase preparation also showed rapid decolorization of 800 mg/L NC, and the decolorization efficiencies were 82.48%, 91.39%, and 97.61% after 1, 2, and 3 h, respectively. In contrast, decolorization was slow for RB5 and AO7. After 1, 3, and 6 h, the decolorization efficiencies were 37.33%, 67.05%, and 70.09%, respectively, for 100 mg/L RB5 and 10.15%, 26.22%, and 52.32%, respectively, for 100 mg/L AO7 (Figure 2J,K). For AO7, the decolorization rate gradually slowed down; however, for RB5, the decolorization rate did not significantly change during the process. In summary, the decolorization rate of the three azo dyes followed the order NC > RB5 > AO7.

#### 2.2.3. Kinetic Equations of Azo Dye Degradation

We further investigated the kinetic degradation equations of the azo dyes NC, AO7, and RB5 and calculated the reaction rate constant *k*. As shown in Figure 2L–N, degradation fit the linear regression models well, and the *R*^2^ values were all above 0.95, indicating a high goodness of fit. Therefore, the degradation of NC, AO7, and RB5 followed first-order kinetics, and the *k* values of 100 mg/L NC, RB5, and AO7 were $1.84\times 10^{-2}$, $7.44\times 10^{-3}$, and $7.15\times 10^{-3}$ min^−1^, respectively (Figure 2L–N). The reaction rates of the three azo dyes followed the order NC > RB5 > AO7. The results of the degradation kinetics were consistent with the time courses of decolorization.

### 2.3. Decolorization of the Anthraquinone Dyes

#### 2.3.1. Decolorization Efficiency of Anthraquinone Dyes at Different Concentrations

The crude laccase preparation demonstrated excellent decolorization of the anthraquinone dyes RBBR and RB4 at different concentrations. As shown in Figure S1A,B, the 24 h decolorization efficiencies were 98.73% and 95.52% for RBBR and RB4, respectively, at 100 mg/L, 90.70% and 87.26% at 200 mg/L, and 90.25% and 80.66% at 800 mg/L. Therefore, the decolorization efficiency remained high for RBBR and RB4 at high concentrations.

#### 2.3.2. Time Courses of Decolorization of Anthraquinone Dyes at Different Concentrations

As shown in Figure S1C,D, the crude laccase preparation demonstrated very rapid degradation of RBBR at different concentrations. The decolorization efficiencies of 100 mg/L RBBR at 1, 2, and 3 h were 88.03%, 95.97%, and 98.97%, respectively. The crude laccase preparation also showed rapid decolorization of RB4, with decolorization efficiencies of 55.88%, 72.43%, and 81.54% for 100 mg/L RB4 at 0.5, 1, and 2 h, respectively. These results indicated that the crude laccase preparation could rapidly decolorize anthraquinone dyes, and the decolorization rate followed the order RBBR > RB4.

#### 2.3.3. Kinetic Equations of Anthraquinone Dye Degradation

The degradation kinetic equations of 100 mg/L anthraquinone dyes RBBR and RB4 were plotted and the *k* values were determined. As shown in Figure S1E,F, the degradation of these anthraquinone dyes fit the linear regression models well, with *R*^2^ values above 0.95, indicating a high goodness of fit. Therefore, the degradation of RBBR and RB4 followed first-order kinetics, and the *k* values of 100 mg/L RBBR and RB4 were $6.05\times 10^{-2}$ and $1.81\times 10^{-2}$ min^−1^, respectively (Figure S1E,F). These results showed that the crude laccase preparation could rapidly degrade both RBBR and RB4, and the reaction rate was higher for RBBR than RB4. The degradation kinetics results were consistent with the time courses of decolorization.

### 2.4. Decolorization of Triphenylmethane Dyes

#### 2.4.1. Decolorization Efficiency of Triphenylmethane Dyes at Different Concentrations

The crude laccase preparation demonstrated different decolorization efficiencies for the triphenylmethane dyes. The decolorization efficiency was excellent for MG and BB at different concentrations, good for CR, and poor for AF. As shown in Figure S2A,B, the 24 h decolorization efficiencies were 89.42% and 99.50% for MG and BB, respectively, at 50 mg/L, 94.27% and 99.50% at 200 mg/L, and 84.04% and 99.02% at 800 mg/L, indicating very efficient decolorization. The 24 h decolorization efficiencies of 50, 100, 200, 400, and 800 mg/L CR were 52.00%, 58.84%, 56.36%, 53.37%, and 50.12%, respectively (Figure S2C), indicating efficient decolorization. The 24 h decolorization efficiencies of AF at different concentrations were all below 10% (Figure S2D), indicating inefficient decolorization. In summary, the decolorization efficiency of triphenylmethane dyes followed the order BB > MG > CR > AF.

#### 2.4.2. Time Courses of Decolorization of the Triphenylmethane Dyes at Different Concentrations

As shown in Figure S2F, the crude laccase preparation showed extremely rapid decolorization of BB. The decolorization efficiencies of 100 mg/L BB after 1, 2, and 3 h were 89.52%, 96.48%, and 98.07%, respectively, while the decolorization efficiencies of 800 mg/L BB after 1, 2, and 3 h were 86.58%, 93.49%, and 96.04%, respectively. As shown in Figure S2E, the crude laccase preparation also showed rapid decolorization of MG. The decolorization efficiencies of 100 mg/L MG at 3, 6, 9, and 12 h were 85.79%, 91.57%, 95.18%, and 98.56%, respectively. Decolorization slowed down with increasing MG concentration, and the decolorization efficiencies of 800 mg/L MG at 3, 6, 9, and 12 h were 38.34%, 57.84%, 64.35%, and 67.25%, respectively.

#### 2.4.3. Kinetic Equations of Triphenylmethane Dye Degradation

The degradation kinetic equations of 100 mg/L triphenylmethane dyes BB and MG were investigated and the *k* values were determined. As shown in Figure S2G,H, the degradation of these dyes fit well with the linear regression models and the *R*^2^ values were above 0.95, indicating a high goodness of fit. Therefore, the degradation of BB and MG followed first-order kinetics. The *k* values of 100 mg/L BB and MG were $4.344\times 10^{-2}$ and $7.232\times 10^{-4}$ min^−1^, respectively (Figure S2G,H), and these results showed that the crude laccase preparation could rapidly degrade BB and the reaction rate of BB was significantly higher than that of MG.

### 2.5. Decolorization of Indigo Dye

#### 2.5.1. Decolorization Efficiency of Indigo Dye at Different Concentrations

The crude laccase preparation demonstrated excellent decolorization of IC at different concentrations. The 24 h decolorization efficiencies were 94.81%, 94.93%, 97.24%, 99.13%, and 96.98% for IC at 50, 100, 200, 400, and 800 mg/L, respectively (Figure S3A). Decolorization was nearly complete, indicating excellent decolorization of IC at high concentrations.

#### 2.5.2. Time Courses of Decolorization of Indigo Dye at Different Concentrations

As shown in Figure S3B, the crude laccase preparation demonstrated rapid decolorization of 50 mg/L IC. The decolorization efficiencies at 1, 2, and 3 h were 86.25%, 95.55%, and 100%, respectively, and with 100 mg/L IC, the decolorization rate was slightly affected. The decolorization efficiencies of 100 mg/L IC at 3, 6, and 9 h were 66.05%, 90.58%, and 93.77%, respectively. With a further increase in IC concentration, the decolorization rate slowed down. The decolorization efficiencies of 800 mg/L IC at 3, 6, 9, and 12 h were 59.39%, 81.47%, 94.44%, and 98.21%, respectively (Figure S3B).

#### 2.5.3. Kinetic Equations of Indigo Dye Degradation

As shown in Figure S3C, the degradation of IC fit well with the linear regression model, with an *R*^2^ value above 0.95, indicating a high goodness of fit. Therefore, the degradation of IC followed first-order kinetics. The *k* value of 100 mg/L IC was $3.05\times 10^{-3}$ min^−1^, indicating rapid degradation of IC by the crude laccase preparation.

### 2.6. Comparison and Ranking of k Values of Various Dyes

The crude laccase preparation showed extremely rapid degradation of the anthraquinone dye RBBR and the triphenylmethane dye BB, with *k* values of $6.05\times 10^{-2}$ min^−1^ and $4.34\times 10^{-2}$ min^−1^, respectively. The decolorization was rapid for the azo dye NC and the anthraquinone dye RB4, with *k* values of $1.84\times 10^{-2}$ min^−1^ and $1.81\times 10^{-2}$ min^−1^, respectively, while the decolorization was slow for the azo dyes RB5 and AO7 as well as the indigo dye IC and the triphenylmethane dye MG, with *k* values of $7.44\times 10^{-3}$ min^−1^, $7.15\times 10^{-3}$ min^−1^, $3.05\times 10^{-3}$ min^−1^, and 7.23 $\times 10^{-4}$ min^−1^_,_ respectively. In summary, the *k* values followed the order RBBR > BB > NC > RB4 > RB5 > AO7 > IC > MG. A summary table for the reaction rate constant of single dyes is shown in Table 1.

### 2.7. Decolorization of Mixed Dyes with Crude Laccase Preparation: Determination of Decolorization Efficiency

#### 2.7.1. Decolorization of Azo + Azo Dyes

**Figure 3:**
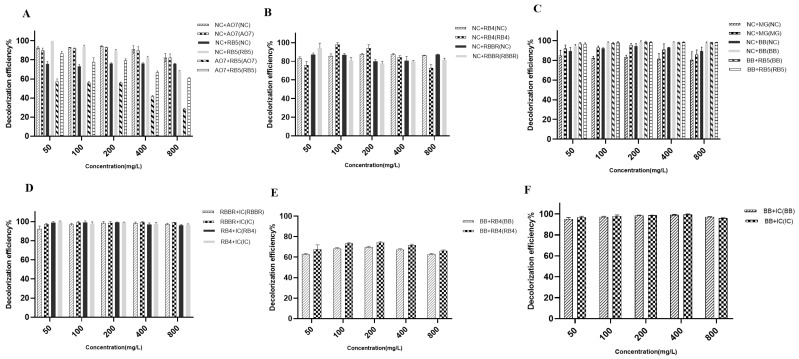
Decolorization of mixed dyes with the crude laccase from *P. eryngii*—determination of decolorization efficiency of each dye in the dye mixture. (**A**) Decolorization of azo + azo dyes. NC + AO7: New Coccine + Acid Orange 7 (each with final concentrations of 50, 100, 200, 400, and 800 mg/L); NC + RB5: New Coccine + Reactive Black 5 (each with final concentrations of 50, 100, 200, 400, and 800 mg/L); AO7 + RB5: Acid Orange 7 + Reactive Black 5 (each with final concentrations of 50, 100, 200, 400, and 800 mg/L). (**B**) Decolorization of azo + anthraquinone dyes. NC + RB4: New Coccine + Reactive Blue 4; NC + RBBR: New Coccine + Remazol Brilliant Blue R. (**C**) Decolorization of azo + triphenylmethane dyes. NC + MG: New Coccine + Methyl Green; NC + BB: New Coccine + Bromophenol Blue; BB + RB5: Bromophenol Blue + Reactive Black 5. (**D**) Decolorization of anthraquinone + indigo dyes. RBBR + IC: Remazol Brilliant Blue R + Indigo Carmine; RB4 + IC: Reactive Blue 4 + Indigo Carmine. (**E**) Decolorization of anthraquinone + triphenylmethane dyes. BB + RB4: Bromophenol Blue + Reactive Blue 4. (**F**) Decolorization of triphenylmethane + indigo dyes. BB + IC: Bromophenol Blue + Indigo Carmine.

The crude laccase preparation showed efficient decolorization of the azo + azo dyes. For NC + AO7, the decolorization efficiency was similar for NC and AO7 at different concentrations. The 24 h decolorization efficiencies exceeded 85% for 50, 100, 200, and 400 mg/L NC and AO7, while the 24 h decolorization efficiencies were 84.81% and 84.42% for 800 mg/L NC and AO7, respectively (Figure 3A). For NC + RB5, the 24 h decolorization efficiencies of NC and RB5 were 76.25% and 88.86%, respectively, at 200 mg/L, 76.22% and 82.39% at 400 mg/L, and 75.94% and 68.29% at 800 mg/L (Figure 3A). In summary, the crude laccase preparation demonstrated efficient decolorization of azo + azo dyes.

#### 2.7.2. Decolorization of Azo + Anthraquinone Dyes

The crude laccase preparation demonstrated efficient decolorization of the azo + anthraquinone dyes at different concentrations. For NC + RB4, the decolorization efficiency was approximately 85% for NC at different concentrations. With an increase in dye concentration, the decolorization efficiency of RB4 gradually decreased. But the decolorization efficiency of RB4 in the mixture remained high at different concentrations (Figure 3B). For NC + RBBR, the 24 h decolorization efficiencies were 87.24% and 91.25% for NC and RBBR, respectively, at 50 mg/L, 86.93% and 80.65% at 100 mg/L, 80.72% and 79.97% at 400 mg/L, and 78.20% and 77.89% at 800 mg/L (Figure 3B). Thus, the crude laccase preparation also demonstrated efficient decolorization of NC + RBBR dyes.

#### 2.7.3. Decolorization of Azo + Triphenylmethane Dyes

The crude laccase preparation showed efficient decolorization of the azo + triphenylmethane dyes. For NC + MG, the decolorization efficiency was approximately 85% for NC at different concentrations, and the efficiency was slightly higher for MG (approximately 90%). Compared to single dyes, the decolorization efficiency decreased for NC, but increased for MG (Figure 3C). For NC + BB, the 24 h decolorization efficiencies were 89.58% and 94.55% for NC and BB, respectively, at 50 mg/L, 92.21% and 97.76% at 100 mg/L, 93.12% and 98.37% at 400 mg/L, and 89.49% and 97.44% at 800 mg/L (Figure 3C). For RB5 + BB, the 24 h decolorization efficiencies were 98.26% and 98.03% for 400 mg/L RB5 and BB, respectively, and 98.01% and 98.19% for 800 mg/L RB5 and BB, respectively (Figure 3C). Thus, the crude laccase preparation demonstrated efficient decolorization of the NC + BB and RB5 + BB dyes.

#### 2.7.4. Decolorization of Anthraquinone + Indigo Dyes

The crude laccase preparation demonstrated very efficient decolorization of anthraquinone + indigo dyes. For RBBR + IC and RB4 + IC, the decolorization efficiency exceeded 95% for all dyes, with no significant differences in decolorization efficiency compared to the single dyes. For RBBR + IC, the 24 h decolorization efficiencies were 98.68% and 99.35% for RBBR and IC, respectively, at 200 mg/L, 98.38% and 99.30% at 400 mg/L, and 97.65% and 98.95% at 800 mg/L (Figure 3D). For RB4 + IC, the 24 h decolorization efficiencies were 99.21% and 98.95% for RB4 and IC, respectively, at 200 mg/L, 97.04% and 97.38% at 400 mg/L, and 96.25% and 96.86% at 800 mg/L (Figure 3D).

#### 2.7.5. Decolorization of Anthraquinone + Triphenylmethane Dyes

The crude laccase preparation showed efficient decolorization of the anthraquinone + triphenylmethane dyes. For BB + RB4, the 24 h decolorization efficiencies were 68.47% and 73.51% for BB and RB4, respectively, at 100 mg/L, 69.83% and 74.13% at 200 mg/L, 67.48% and 71.83% at 400 mg/L, and 62.75% and 66.20% at 800 mg/L (Figure 3E).

#### 2.7.6. Decolorization of Triphenylmethane + Indigo Dyes

The crude laccase preparation showed very efficient decolorization of the triphenylmethane + indigo dyes (BB + IC), and the experiment demonstrated similar 24 h decolorization efficiencies (>90%) for BB and IC at different concentrations, with no significant differences in decolorization efficiency compared to the single dyes. The 24 h decolorization efficiencies were 98.62% and 98.77% for BB and IC, respectively, at 200 mg/L, 99.13% and 99.47% at 400 mg/L, and 97.33% and 96.16% at 800 mg/L (Figure 3F).

### 2.8. Decolorization Kinetics of Mixed Dyes

#### 2.8.1. Decolorization Kinetics of Azo + Azo Dyes

The decolorization kinetics of the azo + azo dyes (100 mg/L NC + 100 mg/L RB5) were investigated with 100 mg/L NC or 100 mg/L RB5 as the controls. The ln *c*-*t* curves were plotted for the mixed and single dyes.

As shown in Figure S4A, the *k* value of NC decreased from $1.84\times 10^{-2}$ min^−1^ in the control (decolorization of single dye) to $6.04\times 10^{-3}$ min^−1^ in NC + RB5 (decolorization of mix dyes), and the *k* value of RB5 decreased from $7.44\times 10^{-3}$ min^−1^ in the control to $2.76\times 10^{-3}$ min^−1^ in NC + RB5. Therefore, in NC + RB5, the *k* values (decolorization rates) of both dyes decreased compared to the single dyes, with NC and RB5 showing mutual inhibition during decolorization of the two azo dyes.

#### 2.8.2. Decolorization Kinetics of Azo + Anthraquinone Dyes

The degradation kinetics of the azo + anthraquinone dyes (100 mg/L NC + 100 mg/L RBBR and 100 mg/L NC + 100 mg/L RB4) were investigated using single dyes as the controls (100 mg/L NC, 100 mg/L RBBR, and 100 mg/L RB4). The ln *c*-*t* curves were plotted for the mixed and single dyes.

As shown in Figure S4B, the *k* value of NC increased from $1.84\times 10^{-2}$ min^−1^ in the control to $2.00\times 10^{-2}$ min^−1^ in NC + RB4, while the *k* value of RB4 decreased from $1.81\times 10^{-2}$ min^−1^ in the control to $1.28\times 10^{-2}$ min^−1^ in NC + RB4. As shown in Figure S4C, the *k* value of NC increased from $1.84\times 10^{-2}$ min^−1^ in the control to $2.28\times 10^{-2}$ min^−1^ in NC + RBBR, while the *k* value of RBBR decreased from 6.05$\;\times \;10^{-2}$ min^−1^ in the control to 2.68$\;\times \;10^{-2}$ min^−1^ in NC + RBBR. These results indicated that during the decolorization of azo + anthraquinone dyes, the presence of the anthraquinone dye significantly enhanced the decolorization rate of the azo dye, while the decolorization rate of the anthraquinone dye was inhibited. 

#### 2.8.3. Decolorization Kinetics of Azo + Indigo Dyes

As shown in Figure S4D, the *k* value of NC significantly decreased from 1.84$\;\times \;10^{-2}$ min^−1^ in the control to 7.58$\;\times \;10^{-4}$ min^−1^ in NC + IC, while the *k* value of IC increased from 3.05$\;\times \;10^{-3}$ min^−1^ in the control to 4.62$\;\times \;10^{-3}$ min^−1^ in NC + IC. These results indicated that in the decolorization of azo + indigo dyes, the decolorization rate of the indigo dye increased while the decolorization rate of the azo dye significantly decreased compared to the single dyes. In NC + IC, the azo dye promoted the decolorization of the indigo dye, while the indigo dye inhibited the decolorization of the azo dye.

#### 2.8.4. Decolorization Kinetics of Azo + Triphenylmethane Dyes

As shown in Figure S4E, the *k* value of NC decreased from 1.84$\;\times \;10^{-2}$ min^−1^ in the control to 9.25$\;\times \;10^{-3}$ min^−1^ in NC + MG, while the *k* value of MG increased from 7.23$\;\times \;10^{-4}$ min^−1^ in the control to 3.30$\;\times \;10^{-3}$ min^−1^ in NC + MG. These results indicated that in the decolorization of azo + triphenylmethane dyes, the azo dye NC promoted the decolorization rate of the triphenylmethane dye MG, while MG inhibited the decolorization rate of NC.

#### 2.8.5. Decolorization Kinetics of Anthraquinone + Indigo Dyes

As shown in Figure S4F, the *k* value of RB4 increased from 1.81$\;\times \;10^{-2}$ min^−1^ in the control to 7.01$\;\times \;10^{-2}$ min^−1^ in RB4 + IC, and the *k* value of IC increased from 3.05$\;\times \;10^{-3}$ min^−1^ in the control to 6.17$\;\times \;10^{-2}$ min^−1^ in RB4 + IC. The reaction rate of IC greatly increased. These results indicated that in the decolorization of anthraquinone + indigo dyes, the anthraquinone dye RB4 significantly enhanced the decolorization rate of the indigo dye IC.

#### 2.8.6. Decolorization Kinetics of Indigo + Triphenylmethane Dyes

As shown in Figure S5A, the *k* value of CR decreased from 1.6$2\;\times \;10^{-2}$ min^−1^ in the control to 1.00$\;\times \;10^{-2}$ min^−1^ in IC + CR, while the *k* value of IC increased from 3.05$\;\times \;10^{-3}$ min^−1^ in the control to $1.55\;\times \;10^{-1}$ min^−1^ in IC + CR. The improvement effect was very obvious. These results indicated that in the decolorization of indigo + triphenylmethane dyes, the triphenylmethane dye CR significantly promoted the decolorization rate of the indigo dye IC, while IC inhibited the decolorization rate of CR.

#### 2.8.7. Decolorization Kinetics of Anthraquinone + Triphenylmethane Dyes

As shown in Figure S5B, the *k* value of RBBR decreased from 6.05$\;\times \;10^{-2}$ min^−1^ in the control to 3.46$\;\times \;10^{-2}$ min^−1^ in RBBR + CR, while the *k* value of CR increased from 1.62$\;\times \;10^{-2}$ min^−1^ in the control to 1.81$\;\times \;10^{-2}$ min^−1^ in RBBR + CR. These results indicated that in the decolorization of anthraquinone + triphenylmethane dyes, the decolorization rate of the triphenylmethane dye CR was promoted, but the decolorization rate of the anthraquinone dye RBBR was inhibited compared to the single dyes. In RBBR + CR, RBBR promoted the decolorization rate of CR, while CR inhibited the decolorization rate of RBBR.

#### 2.8.8. Decolorization Kinetics of Triphenylmethane + Triphenylmethane Dyes

As shown in Figure S5C, in the single and mixed dyes, the concentrations of both MG and BB were 100 mg/L. The *k* value of MG increased from 7.23$\;\times \;10^{-4}$ min^−1^ in the control to 5.78$\;\times \;10^{-3}$ min^−1^ in MG + BB, while the *k* value of BB decreased from 4.34$\;\times \;10^{-2}$ min^−1^ in the control to 3.34$\;\times \;10^{-3}$ min^−1^ in MG + BB. As shown in Figure S5D, the *k* value of MG increased significantly from 7.23$\;\times \;10^{-4}$ min^−1^ in the control to 1.48$\;\times \;10^{-2}$ min^−1^ in MG + CR, while the *k* value of CR decreased from 1.62$\;\times \;10^{-2}$ min^−1^ in the control to 1.11$\;\times \;10^{-2}$ min^−1^ in MG + CR. In addition, the *k* value of AF increased from 3.87$\;\times \;10^{-4}$ min^−1^ in the control to 7.8$\;\times \;10^{-3}$ min^−1^ in BB + AF, while the *k* value of BB decreased from 4.34$\;\times \;10^{-2}$ min^−1^ in the control to 1.59$\;\times \;10^{-2}$ min^−1^ in BB + AF (Figure S5E). These results indicated that in the decolorization of triphenylmethane + triphenylmethane dyes, the decolorization rate of one triphenylmethane dye was significantly enhanced, while the decolorization rate of the other triphenylmethane dye was inhibited. In a mixture containing two triphenylmethane dyes, the first dye significantly promoted the decolorization rate of the second dye, while the second dye inhibited the decolorization rate of the first dye.

A summary table for the reaction rate constant of dye mixtures was shown in Table 2.

### 2.9. Effects of Metal Salts at Different Concentrations on the Degradation of Dyes: Metal Salt Tolerance

#### 2.9.1. Effects of Metal Salts at Different Concentrations on the Degradation of the Azo Dye NC

**Figure 4:**
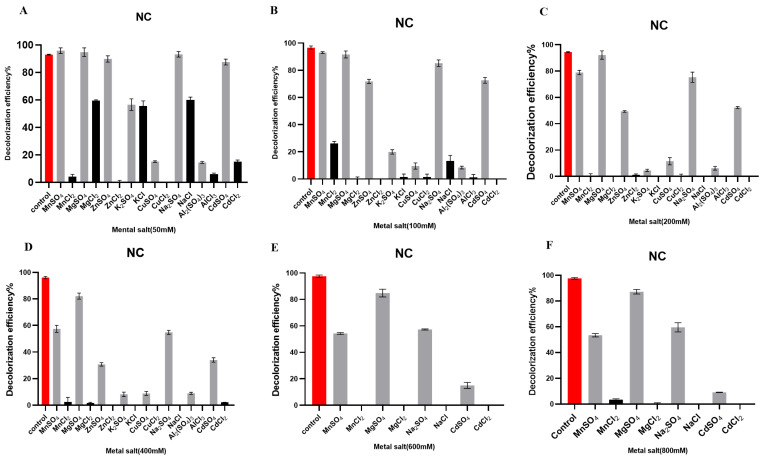
Effects of metal salts at different concentrations on the degradation of the azo dye NC (New Coccine) with the crude laccase from *P. eryngii*. The metal salts used in this study were MnSO_4_, MnCl_2_, MgSO_4_, MgCl_2_, ZnSO_4_, ZnCl_2_, K_2_SO_4_, KCl, CuSO_4_, CuCl_2_, Na_2_SO_4_, NaCl, Al_2_(SO_4_)_3_, AlCl_3_, CdSO_4_, and CdCl_2_. The control group consisted of a 1 mL decolorization system without metal salts. (**A**) The final concentration of metal salt was 50 mM. (**B**) The final concentration of metal salt was 100 mM. (**C**) The final concentration of metal salt was 200 mM. (**D**) The final concentration of metal salt was 400 mM. (**E**) The final concentration of metal salt was 600 mM. (**F**) The final concentration of metal salt was 800 mM. Red color: control, no metal salts added. Grey color: SO_4_^2−^ salts. Black color: Cl^−^ salts.

The crude laccase preparation showed a high tolerance to SO_4_^2−^ salts such as MnSO_4_, MgSO_4_, ZnSO_4_, and Na_2_SO_4_ during the degradation of NC. With 50 mM of MnSO_4_, MgSO_4_, ZnSO_4_, and Na_2_SO_4_, the 24 h decolorization efficiencies of NC were 95.75%, 95.06%, 89.06%, and 93.02%, respectively. With 200 mM salts, the decolorization was not significantly affected, and the 24 h decolorization efficiencies were 79.97%, 92.70%, 49.68%, and 79.00%, respectively. With 800 mM salts, the laccase remained active and the 24 h decolorization efficiencies were 52.13%, 86.44%, 30.65%, and 63.64%, respectively (Figure 4).

The crude laccase preparation showed a low tolerance to Cl^−^ salts during NC decolorization. With 50 mM Cl^−^ salts, the enzyme preparation was tolerant to NaCl, KCl, NiCl, and MgCl_2_, and the 24 h decolorization efficiencies were 59.60%, 55.09%, 57.22%, and 56.68%, respectively. With 100 mM Cl^−^ salts, the tolerance was greatly reduced, while with ≥200 mM Cl^−^ salts, the 24 h decolorization efficiency of NC was almost zero.

Compared to the SO_4_^2−^ salts, the Cl^−^ salts demonstrated significantly stronger inhibition for NC degradation. With 100 mM MnSO_4_ and MgSO_4_, the 24 h decolorization efficiencies were 93.25% and 91.59%, respectively, and NC decolorization was slightly affected. With 100 mM MnCl_2_ and MgCl_2_, the decolorization efficiencies were only 26.44% and 1.65%, respectively. Compared to the 100 mM SO_4_^2−^ salts, the 100 mM Cl^−^ salts significantly inhibited NC degradation (Figure 4B). These results indicated that MnCl_2_ and MgCl_2_ exhibited significant inhibition of the decolorization of NC.

#### 2.9.2. Effects of Metal Salts at Different Concentrations on the Degradation of the Anthraquinone Dye RBBR

The crude laccase preparation showed a high tolerance to SO_4_^2−^ salts such as MnSO_4_, MgSO_4_, ZnSO_4_, Na_2_SO_4_, K_2_SO_4_, and CdSO_4_ during the degradation of RBBR. With 50 mM MnSO_4_, MgSO_4_, ZnSO_4_, Na_2_SO_4_, K_2_SO_4_, and CdSO_4_, decolorization was efficient and the 24 h decolorization efficiencies were 100.00%, 74.29%, 77.03%, 100.00%, 100.00%, and 81.18%, respectively. With 200 mM SO_4_^2−^ salts, decolorization remained efficient and the decolorization efficiencies were 86.49%, 87.02%, 91.24%, 88.96%, 40.30%, and 83.27%, respectively. With 800 mM MnSO_4_, MgSO_4_, ZnSO_4_, Na_2_SO_4_, and CdSO_4_, the 24 h decolorization efficiencies were 34.39%, 55.58%, 57.18%, 90.32%, and 76.67%, respectively, and laccase remained active for RBBR decolorization (Figure S6). The crude laccase preparation demonstrated the highest tolerance to Na_2_SO_4_ during RBBR degradation, and with 800 mM Na_2_SO_4_, the 24 h decolorization efficiency of RBBR still reached 88.72%, which was close to the control without salts (Figure S6F).

The crude laccase preparation demonstrated a low tolerance to the Cl^−^ salts during RBBR degradation. With 400 mM Cl^−^ salts, RBBR decolorization was significantly inhibited, except for CdCl_2_ and LiCl, and the 24 h decolorization efficiency was below 10%.

Compared to the SO_4_^2−^ salts, the Cl^−^ salts showed a significantly stronger inhibition of RBBR degradation. With 100 mM MnSO_4_ and MgSO_4_, the 24 h decolorization efficiencies were 100.00% and 68.94%, respectively, and with 100 mM MnCl_2_ and MgCl_2_, the 24 h decolorization efficiencies decreased to 24.81% and 21.43%, respectively (Figure S6B). Therefore, the Cl^−^ salts were stronger inhibitors of RBBR degradation compared to the SO_4_^2−^ salts.

#### 2.9.3. Effects of Metal Salts at Different Concentrations on the Degradation of the Triphenylmethane Dye BB

The crude laccase preparation showed a high tolerance to SO_4_^2−^ salts such as MnSO_4_, MgSO_4_, ZnSO_4_, Na_2_SO_4_, K_2_SO_4_, and CdSO_4_ during the degradation of BB. With 50 mM MnSO_4_, MgSO_4_, ZnSO_4_, Na_2_SO_4_, K_2_SO_4_, and CdSO_4_, BB decolorization was slightly affected, and with 200 mM MnSO_4_, MgSO_4_, ZnSO_4_, Na_2_SO_4_, K_2_SO_4_, and CdSO_4_, the 24 h decolorization efficiencies were 90.08%, 95.75%, 93.87%, 97.38%, 83.59%, and 95.98%, respectively, indicating that decolorization was efficient. With 800 mM MnSO_4_, MgSO_4_, ZnSO_4_, Na_2_SO_4_, and CdSO_4_, the 24 h decolorization efficiencies were 88.89%, 98.26%, 91.76%, 96.91%, and 95.98%, respectively (Figure S7), and decolorization remained efficient. These results indicated that the crude laccase preparation was tolerant to high concentrations of SO_4_^2−^ salts during the degradation of BB.

The crude laccase preparation showed a low tolerance to Cl^−^ salts during the degradation of BB. With ≥600 mM Cl^−^ salts, BB degradation was significantly inhibited and the decolorization efficiency was almost zero.

Compared to the SO_4_^2−^ salts, Cl^−^ salts showed significantly stronger inhibition of BB degradation. With 100 mM MnSO_4_ and MgSO_4_, the 24 h decolorization efficiencies were 96.37% and 98.05%, respectively, and the decolorization of BB was slightly affected. With 100 mM MnCl_2_ and MgCl_2_, the decolorization efficiencies decreased to 42.19% and 73.09%, respectively, and MnCl_2_ and MgCl_2_ demonstrated a remarkable inhibition of BB decolorization (Figure S7B). With 200 mM MnSO_4_ and MgSO_4_, the 24 h decolorization efficiencies were 95.69% and 100.00%, respectively, and with 200 mM MnCl_2_ and MgCl_2_, the 24 h decolorization efficiencies significantly decreased to 0% and 45.47%, respectively (Figure S7C). The decrease in decolorization efficiencies was very significant.

#### 2.9.4. Effects of Metal Salts at Different Concentrations on the Degradation of the Indigo Dye IC

The crude laccase preparation showed a moderate tolerance to SO_4_^2−^ salts such as MnSO_4_, MgSO_4_, ZnSO_4_, Na_2_SO_4_, and CdSO_4_, as well as a low tolerance to Cl^−^ salts during IC degradation (Figure S8). With 50 mM Cl^−^ salts, IC degradation was significantly inhibited, except for CdCl_2_ and NiCl_2_, while for ≥200 mM Cl^−^ salts, stronger inhibition was recorded. Compared to the SO_4_^2−^ salts, Cl^−^ salts demonstrated stronger inhibition of IC decolorization (Figure S8).

### 2.10. Effects of Organic Solvents at Different Concentrations on the Degradation of Dyes: Organic Solvent Tolerance

#### 2.10.1. Effects of Organic Solvents at Different Concentrations on the Degradation of the Azo Dye NC

**Figure 5:**
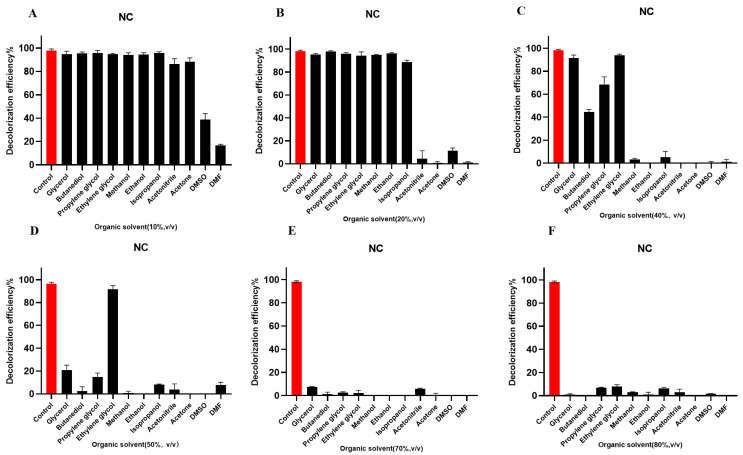
Effects of organic solvents at different concentrations on the degradation of the azo dye NC (New Coccine) with the crude laccase from *P. eryngii*. The organic solvents used in this study were glycerol, butanediol, propylene glycol, ethylene glycol, methanol, ethanol, isopropanol, acetonitrile, acetone, DMSO, and DMF. The control group consisted of a 1 mL decolorization system without metal salts. (**A**) The final concentration of organic solvent was 10% (*v*/*v*). (**B**) The final concentration of organic solvent was 20% (*v*/*v*). (**C**) The final concentration of organic solvent was 40% (*v*/*v*). (**D**) The final concentration of organic solvent was 50% (*v*/*v*). (**E**) The final concentration of organic solvent was 70% (*v*/*v*). (**F**) The final concentration of organic solvent was 80% (*v*/*v*). Red color: control, no organic solvents added. Black color: different organic solvents added.

As shown in Figure 5A, the degradation of NC was slightly affected by 10% (*v*/*v*) glycerol, butanediol, propanediol, ethylene glycol, methanol, ethanol, acetonitrile, isopropanol, and acetone, while 10% (*v*/*v*) DMSO and DMF significantly inhibited NC decolorization. The degradation of NC was slightly affected by 20% (*v*/*v*) glycerol, butanediol, propanediol, ethylene glycol, methanol, ethanol, and isopropanol, though 20% (*v*/*v*) acetonitrile and acetone significantly inhibited the decolorization of NC with the crude laccase (Figure 5B). The crude laccase preparation showed a strong tolerance to 40% (*v*/*v*) glycerol, ethylene glycol, propanediol, and butanediol during NC degradation, and the 24 h decolorization efficiencies were 91.53%, 93.94%, 68.50%, and 44.44%, respectively. However, 40% (*v*/*v*) of other organic solvents almost completely inhibited NC decolorization and the decolorization efficiency was almost zero (Figure 5C). The enzyme preparation was still tolerant to 50% (*v*/*v*) ethylene glycol, glycerol, and propanediol, and the 24 h decolorization efficiencies were 91.80%, 20.72%, and 14.87%, respectively (Figure 5D). At 70% (*v*/*v*), all organic solvents demonstrated significant inhibitory effects, and the 24 h decolorization efficiencies were below 10% (Figure 5E). In summary, among the 11 organic solvents, the crude laccase preparation showed high tolerance to ethylene glycol, glycerol, propanediol, and butanediol, with the highest tolerance to ethylene glycol during NC degradation. In contrast, DMSO and DMF demonstrated strong inhibitory effects for NC decolorization (low tolerance to DMSO and DMF).

#### 2.10.2. Effects of Organic Solvents at Different Concentrations on the Degradation of the Anthraquinone Dye RBBR

As shown in Figure S9A, RBBR degradation was slightly affected by 10% (*v*/*v*) glycerol, butanediol, propanediol, ethylene glycol, methanol, ethanol, acetonitrile, isopropanol, and acetone. In addition, RBBR degradation was slightly influenced by 20% (*v*/*v*) glycerol, butanediol, propanediol, ethylene glycol, methanol, and ethanol, though the crude laccase preparation showed a decreased tolerance to 20% (*v*/*v*) isopropanol (Figure S9B). The crude laccase preparation still showed a high tolerance to 40% and 50% (*v*/*v*) glycerol, butanediol, and propanediol, and the 24 h decolorization efficiencies were 78.36%, 86.97%, and 62.56% for glycerol, butanediol, and propanediol, respectively, at 40%, and 39.76%, 41.34%, and 38.89% at 50% (Figure S9C,D). Even with 80% (*v*/*v*) glycerol and butanediol, the 24 h decolorization efficiencies could still reach 16.36% and 26.82%, respectively (Figure S9F).

#### 2.10.3. Effects of Organic Solvents at Different Concentrations on the Degradation of the Triphenylmethane Dye BB

As shown in Figure S10, among the 11 organic solvents, the crude laccase preparation demonstrated the highest tolerance to glycerol for BB degradation. With 10%, 20%, 40%, 50%, and 70% glycerol, the 24 h decolorization efficiencies were 99.66%, 100.00%, 100.00%, 100.00%, and 76.20%, respectively, and with 80% (*v*/*v*) glycerol, the 24 h decolorization efficiencies could still reach 50.8% (Figure S10). In addition to glycerol, the crude laccase preparation demonstrated moderate tolerance to ethylene glycol and propanediol during BB degradation.

#### 2.10.4. Effects of Organic Solvents at Different Concentrations on the Degradation of the Indigo Dye IC

As shown in Figure S11, among the 11 organic solvents, the crude laccase preparation showed the highest tolerance to glycerol and ethylene glycol during the degradation of IC. With 10%, 20%, 40%, 50%, and 70% glycerol, the 24 h decolorization efficiencies were 99.74%, 100%, 78.83%, 58.51%, and 33.87%, respectively, and with 10%, 20%, 40%, 50%, and 70% ethylene glycol, the 24 h decolorization efficiencies were 99.17%, 98.55%, 71.06%, 61.41%, and 32.14%, respectively (Figure S11). The inhibition of decolorization increased with increasing organic solvent concentration, and at 80% (*v*/*v*), all organic solvents demonstrated significant inhibition of IC decolorization, and the decolorization efficiency of IC was below 20% (Figure S11F).

### 2.11. Repeated-Batch Decolorization of Different Types of Dyes

#### 2.11.1. Repeated-Batch Decolorization of Single Dyes

**Figure 6:**
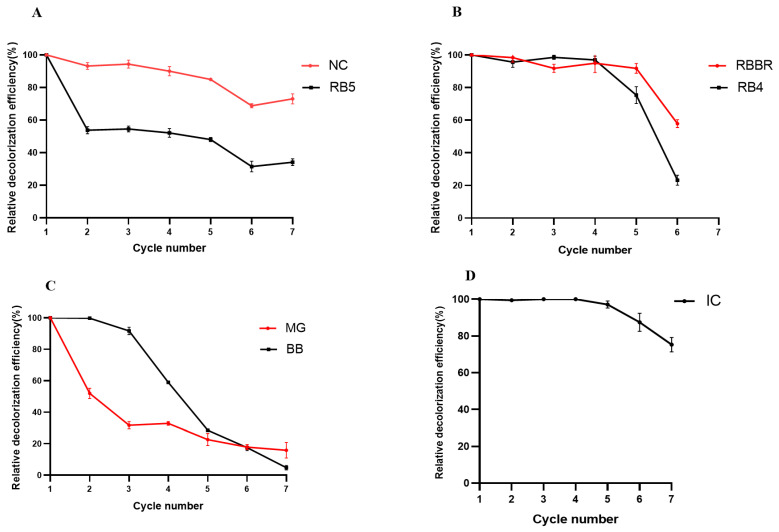
Repeated-batch decolorization of single dyes with the crude laccase from *P. eryngii*. The decolorization efficiency of the first batch was set to 100% for calculation of the relative decolorization efficiency of subsequent batches. Seven consecutive batches of decolorization were carried out in this study. (**A**) Repeated-batch decolorization of the azo dyes NC and RB5. (**B**) Repeated-batch decolorization of the anthraquinone dyes RBBR and RB4. (**C**) Repeated-batch decolorization of the triphenylmethane dyes MG and BB. (**D**) Repeated-batch decolorization of the indigo dye IC.

We investigated the repeated-batch decolorization of the azo dyes NC and RB5. The decolorization efficiency of the first batch was set to 100%, and the relative decolorization efficiency of the following batches (relative to the first batch) was calculated, with the results shown in Figure 6A. The crude laccase preparation showed a high efficiency in the repeated-batch decolorization of the azo dye NC, and in batch 5, the relative decolorization efficiency of NC was 84.85%, while in batch 7, the relative decolorization efficiency of NC was 74.65%, indicating efficient decolorization (Figure 6A). The crude laccase preparation showed a less-efficient repeated-batch decolorization of the azo dye RB5, while in batches 5 and 7, the relative decolorization efficiencies of RB5 were 48.02% and 33.36%, respectively (Figure 6A). These results indicated that the crude laccase demonstrated a significantly higher efficiency in repeated-batch decolorization of NC compared to RB5.

We investigated the repeated-batch decolorization of the anthraquinone dyes RBBR and RB4, with the results shown in Figure 6B. The crude laccase preparation showed efficient repeated-batch decolorization of the anthraquinone dye RBBR, and the relative decolorization efficiencies of batches 2, 3, 4, and 5 were 98.47%, 92.73%, 96.54%, and 93.21%, respectively (Figure 6B). No significant changes in decolorization efficiency were observed in these batches relative to batch 1 (90.46%), and the relative decolorization efficiency of batch 6 was 58.10%, which was significantly lower than batch 1. The crude laccase preparation also showed efficient repeated-batch decolorization of the anthraquinone dye RB4, and the relative decolorization efficiencies in batches 2, 3, and 4 were 94.62%, 98.24%, and 95.24%, indicating no significant changes relative to batch 1 (Figure 6B). The relative decolorization efficiencies of batches 5 and 6 were 76.29% and 24.23%, which were significantly lower than batch 1. These results indicated that repeated-batch decolorization was more efficient for RBBR compared to RB4.

We also investigated the repeated-batch decolorization of the triphenylmethane dyes MG and BB, with the results shown in Figure 6C. The crude laccase preparation showed inefficient repeated-batch decolorization of the triphenylmethane dyes MG and BB. With repeated batches, the relative decolorization efficiency of MG and BB decreased considerably. For BB, the relative decolorization efficiencies of batches 2, 3, 4, 5, and 6 were 99.25%, 93.27%, 59.26%, 33.73%, and 20.51%, respectively, while for MG, the relative decolorization efficiencies of batches 2, 3, 4, 5, and 6 were 53.34%, 35.25%, 37.13%, 23.32%, and 20.86%, respectively (Figure 6C). Repeated-batch decolorization was more efficient for BB than for MG.

We investigated the repeated-batch decolorization of the indigo dye IC, and the results are shown in Figure 6D. The crude laccase preparation showed highly efficient decolorization of IC with repeated batches. The relative decolorization efficiencies of batches 2, 3, 4, and 5 were 100.00%, 100.00%, 99.82%, and 97.23%, respectively. After five consecutive decolorization batches, the relative decolorization efficiency of IC was 97.23%, which was almost unchanged compared to batch 1. In batch 7, the relative decolorization efficiency of IC was 74.42%, which was slightly lower than in batch 1, but the decolorization still remained efficient (Figure 6D).

We also tested the laccase activity changes in each round during the repeated-batch decolorization of single dyes (Figure S13). The initial laccase activity of the crude laccase (cycle 0) was set to 100%, and the relative laccase activity of each cycle (%) was calculated. As shown in Figure S13, the overall trend of laccase activity decreased, but it still maintained a certain level during seven consecutive rounds of degradation. As shown in Figure S13A, during the repeated-batch decolorization of the azo dye NC, using the initial laccase activity (cycle 0) as the control (100%), the relative laccase activities of the first, second, third, fourth, fifth, sixth, and seventh cycles were 65.39%, 44.33%, 42.86%, 38.91%, 39.16%, 36.45%, and 27.34%, respectively. As shown in Figure S13F, during the repeated-batch decolorization of the indigo dye IC, the relative laccase activities of the first, second, third, fourth, fifth, sixth, and seventh cycles were 87.44%, 79.44%, 96.81%, 99.63%, 95.96%, 89.27%, and 78.54%, respectively. As shown in Figure S13G, during the repeated-batch decolorization of triphenylmethane dye MG, the relative laccase activities of the first, second, third, fourth, fifth, sixth, and seventh cycles were 93.10%, 79.06%, 82.02%, 74.87%, 67.24%, 52.95%, and 47.78%, respectively. The above results indicated that during the repeated-batch decolorization of single dyes, the laccase activity of the crude enzyme exhibited good stability. Especially during the repeated-batch decolorization of IC, the laccase activity of the crude laccase remained at a very high level throughout the entire seven rounds of reaction.

#### 2.11.2. Repeated-Batch Decolorization of Two-Dye Mixtures

##### Azo + Anthraquinone Dyes

**Figure 7:**
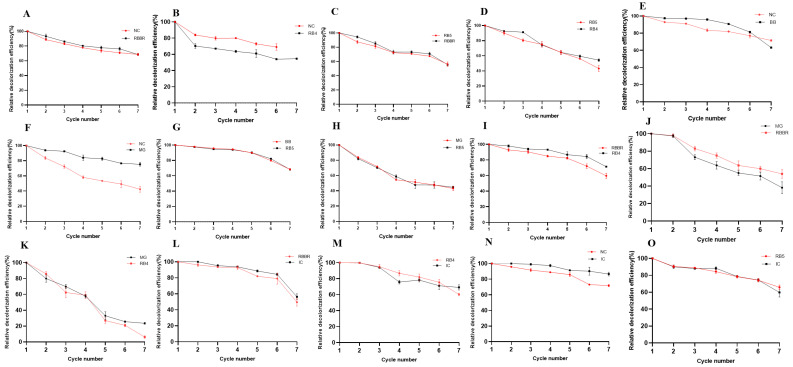
Repeated-batch decolorization of two-dye mixtures with the crude laccase from *P. eryngii*. The decolorization efficiency of the first batch was set to 100% for calculation of the relative decolorization efficiency of subsequent batches. Seven consecutive batches of decolorization were carried out in this study. (**A**) Repeated-batch decolorization of azo + anthraquinone dyes (NC + RBBR). (**B**) Repeated-batch decolorization of azo + anthraquinone dyes (NC + RB4). (**C**) Repeated-batch decolorization of azo + anthraquinone dyes (RB5 + RBBR). (**D**) Repeated-batch decolorization of azo + anthraquinone dyes (RB5 + RB4). (**E**) Repeated-batch decolorization of azo + triphenylmethane dyes (NC + BB). (**F**) Repeated-batch decolorization of azo + triphenylmethane dyes (NC + MG). (**G**) Repeated-batch decolorization of azo + triphenylmethane dyes (RB5 + BB). (**H**) Repeated-batch decolorization of azo + triphenylmethane dyes (RB5 + MG). (**I**) Repeated-batch decolorization of anthraquinone + anthraquinone dyes (RBBR + RB4). (**J**) Repeated-batch decolorization of anthraquinone + triphenylmethane dyes (RBBR + MG). (**K**) Repeated-batch decolorization of anthraquinone + triphenylmethane dyes (RB4 + MG). (**L**) Repeated-batch decolorization of anthraquinone + indigo dyes (RBBR + IC). (**M**) Repeated-batch decolorization of anthraquinone + indigo dyes (RB4 + IC). (**N**) Repeated-batch decolorization of azo + indigo dyes (NC + IC). (**O**) Repeated-batch decolorization of azo + indigo dyes (RB5 + IC).

We investigated the repeated-batch decolorization of azo + anthraquinone dyes and the results are shown in Figure 7A–D. The crude laccase preparation showed efficient decolorization of NC + RBBR in repeated batches, and in NC + RBBR, the relative decolorization efficiencies of the azo dye NC in batches 2, 3, 4, 5, 6, and 7 were 87.23%, 83.76%, 79.13%, 73.72%, 70.94%, and 69.12%, respectively. The relative decolorization efficiencies of the anthraquinone dye RBBR in batches 2, 3, 4, 5, 6, and 7 were 93.47%, 86.58%, 81.37%, 80.38%, 78.72%, and 69.35%, respectively. NC and RBBR were efficiently decolorized in batch 7 (Figure 7A). The crude laccase preparation also showed efficient decolorization of NC + RB4 in repeated batches. In the degradation system of the NC + RB4 mixture, the relative decolorization efficiencies of the azo dye NC in batches 2, 3, 4, 5, and 6 were 83.85%, 80.52%, 81.25%, 74.37%, and 70.26%, respectively, and the relative decolorization efficiencies of the anthraquinone dye RB4 in batches 2, 3, 4, 5, and 6 were 69.07%, 65.27%, 62.19%, 60.79%, and 54.61%, respectively (Figure 7B).

The crude laccase preparation also showed efficient repeated-batch decolorization of RB5 + RBBR and RB5 + RB4. In RB5 + RBBR, the relative decolorization efficiencies of the azo dye RB5 in batches 2, 3, 4, 5, 6, and 7 were 86.27%, 81.95%, 73.34%, 69.62%, 65.18%, and 56.23%, respectively, and the relative decolorization efficiencies of the anthraquinone dye RBBR in batches 2, 3, 4, 5, 6, and 7 were 92.47%, 82.83%, 73.24%, 75.48%, 69.62%, and 55.64%, respectively (Figure 7C). In batch 7, the crude laccase preparation still showed efficient decolorization of RB5 and RBBR, while in RB5 + RB4, the relative decolorization efficiencies decreased with repeated batches. In batch 7, the relative decolorization efficiencies of RB5 and RB4 were 43.25% and 54.27%, respectively (Figure 7D). Thus, laccase remained active for RB5 and RB4 decolorization.

##### Azo + Triphenylmethane Dyes

We subsequently investigated the repeated-batch decolorization of azo + triphenylmethane, with the results shown in Figure 7E–H. The crude laccase preparation showed efficient repeated-batch decolorization of NC and BB in NC + BB. In the degradation system of the NC + BB mixture, the relative decolorization efficiencies of the azo dye NC in batches 2, 3, 4, 5, 6, and 7 were 92.54%, 89.73%, 83.49%, 81.27%, 78.27%, and 71.53%, respectively. The relative decolorization efficiencies of the triphenylmethane dye BB in batches 2, 3, 4, 5, 6, and 7 were 96.89%, 95.72%, 95.94%, 90.97%, 82.43%, and 63.17%, respectively, while in batch 7, the relative decolorization efficiencies of NC and BB were 71.53% and 63.77%, respectively, and decolorization still remained efficient (Figure 7E). The crude laccase preparation showed efficient repeated-batch decolorization of MG in NC + MG, but the relative decolorization efficiency of the azo dye NC decreased with the batches. In NC + MG, the relative decolorization efficiencies of the triphenylmethane dye MG in batches 2, 3, 4, 5, 6, and 7 were 93.24%, 91.72%, 84.01%, 83.59%, 79.47%, and 75.41%, respectively, and the relative decolorization efficiencies of the azo dye NC in batches 2, 3, 4, 5, 6, and 7 were 82.61%, 71.21%, 60.13%, 58.12%, 51.43%, and 42.27%, respectively (Figure 7F).

The crude laccase preparation showed highly efficient repeated-batch decolorization of RB5 + BB. The relative decolorization efficiencies of the azo dye RB5 in batches 2, 3, 4, 5, 6, and 7 were 98.47%, 96.74%, 91.25%, 90.47%, 85.63%, and 68.39%, respectively, and the relative decolorization efficiencies of triphenylmethane dye BB in batches 2, 3, 4, 5, 6, and 7 were 98.56%, 97.14%, 91.43%, 90.72%, 84.53%, and 68.25%, respectively. In batch 7, the relative decolorization efficiencies of BB and RB5 were 68.25% and 68.39%, respectively, and decolorization remained efficient (Figure 7G). The crude laccase preparation also showed efficient repeated-batch decolorization of RB5 + MG, and the relative decolorization efficiencies of the azo dye RB5 in batches 2, 3, 4, 5, 6, and 7 were 80.63%, 68.26%, 59.47%, 48.25%, 49.37%, and 43.52%, respectively. The relative decolorization efficiencies of the triphenylmethane dye MG in batches 2, 3, 4, 5, 6, and 7 were 81.17%, 68.79%, 56.29%, 51.37%, 49.62%, and 42.92%, respectively (Figure 7H). Thus, repeated-batch decolorization was significantly more efficient for RB5 + BB than for RB5 + MG.

##### Azo + Indigo Dyes

We investigated the repeated-batch decolorization of azo + indigo dyes, with the results shown in Figure 7N,O. The crude laccase preparation showed extremely efficient repeated-batch decolorization of the azo + indigo dyes (NC + IC and RB5 + IC). In NC + IC, the relative decolorization efficiencies of the azo dye NC in batches 2, 3, 4, 5, 6, and 7 were 92.16%, 90.47%, 88.42%, 82.14%, 72.79%, and 71.90%, respectively, and the relative decolorization efficiencies of the indigo dye IC in batches 2, 3, 4, 5, 6, and 7 were 99.56%, 97.35%, 95.46%, 91.23%, 90.27%, and 86.60%, respectively. The decolorization was still efficient in batch 7, and the relative decolorization efficiencies of NC and IC were 71.90% and 86.60%, respectively (Figure 7N). In RB5 + IC, the relative decolorization efficiencies of the azo dye RB5 in batches 2, 3, 4, 5, 6, and 7 were 88.54%, 86.75%, 83.33%, 77.34%, 75.6%, and 65.89%, respectively. The relative decolorization efficiencies of the indigo dye IC in batches 2, 3, 4, 5, 6, and 7 were 87.90%, 87.23%, 88.73%, 77.28%, 75.92%, and 59.77%, respectively. The decolorization still remained efficient in batch 7, and the relative decolorization efficiencies of RB5 and IC were 65.89% and 59.77%, respectively (Figure 7O). The crude laccase preparation showed similar efficiency in the repeated-batch decolorization of RB5 and IC in the dye mixture, and the above results indicated that the crude laccase preparation was stable in repeated-batch decolorization of azo + indigo dyes.

##### Anthraquinone + Triphenylmethane Dyes

We investigated the repeated-batch decolorization of anthraquinone + triphenylmethane dyes, with the results shown in Figure 7J,K. The crude laccase preparation showed efficient repeated-batch decolorization of RBBR + MG. In the degradation system of the RBBR + MG mixture, the relative decolorization efficiencies of the anthraquinone dye RBBR in batches 2, 3, 4, 5, 6, and 7 were 97.44%, 81.35%, 74.93%, 65.28%, 61.48%, and 53.96%, respectively. The relative decolorization efficiencies of the triphenylmethane dye MG in batches 2, 3, 4, 5, 6, and 7 were 97.66%, 71.46%, 64.75%, 57.29%, 49.73%, and 38.11%, respectively (Figure 7J), indicating that the crude laccase preparation was more efficient for RBBR than for MG in repeated-batch decolorization of the RBBR + MG mixture. The crude laccase preparation showed moderate efficiency in the repeated-batch decolorization of RB4 + MG, and the relative decolorization efficiencies of RB4 and MG in the mixture significantly decreased with the batches. The relative decolorization efficiencies of the anthraquinone dye RB4 in batches 2, 3, 4, 5, 6, and 7 were 84.37%, 61.27%, 59.99%, 26.84%, 21.18%, and 6.21%, respectively, and the relative decolorization efficiencies of the triphenylmethane dye MG in batches 2, 3, 4, 5, 6, and 7 were 79.48%, 68.26%, 57.75%, 33.24%, 23.78%, and 23.54%, respectively (Figure 7K). In batch 7, the relative decolorization efficiencies of MG and RB4 significantly decreased to 23.54% and 6.21%, respectively, and these results indicated that the crude laccase preparation was significantly more efficient for RBBR + MG than for RB4 + MG in repeated-batch decolorization.

##### Anthraquinone + Indigo Dyes

We investigated the repeated-batch decolorization of anthraquinone + indigo dyes, with the results shown in Figure 7L,M. The crude laccase preparation showed high efficiency in the repeated-batch decolorization of RB4 + IC, and the relative decolorization efficiencies of the anthraquinone dye RB4 in batches 2, 3, 4, 5, 6, and 7 were 98.73%, 91.26%, 84.56%, 82.17%, 78.64%, and 60.27%, respectively, while the relative decolorization efficiencies of the indigo dye IC in batches 2, 3, 4, 5, 6, and 7 were 98.52%, 90.99%, 77.38%, 79.25%, 72.36%, and 69.09%, respectively (Figure 7M). The crude laccase preparation showed similar efficiency in the repeated-batch decolorization of RB4 and IC in the dye mixture. The crude laccase preparation also showed high efficiency in the repeated-batch decolorization of RBBR + IC, where the relative decolorization efficiencies of the anthraquinone dye RBBR in batches 2, 3, 4, 5, 6, and 7 were 94.55%, 91.58%, 91.10%, 80.27%, 79.06%, and 49.51%, respectively, while the relative decolorization efficiencies of the indigo dye IC in batches 2, 3, 4, 5, 6, and 7 were 99.27%, 93.29%, 90.78%, 84.49%, 83.56%, and 56.18%, respectively (Figure 7L).

##### The Change in Laccase Activity during the Repeated-Batch Decolorization of Two-Dye Mixtures

As shown in Figure S14D, during the repeated-batch decolorization of the RBBR + MG mixture, using the initial laccase activity (cycle 0) as the control (100%), the relative laccase activities of the first, second, third, fourth, fifth, sixth, and seventh cycles were 64.53%, 64.78%, 62.31%, 48.27%, 38.91%, 32.51%, and 31.77%, respectively. As shown in Figure S14F, during the repeated-batch decolorization of the RBBR + IC mixture, the relative laccase activities of the first, second, third, fourth, fifth, sixth, and seventh cycles were 53.45%, 50.24%, 43.35%, 35.96%, 26.35%, 23.15%, and 24.14%, respectively. The above results indicated that during the repeated-batch decolorization of two-dye mixtures, the laccase activity of the crude enzyme also exhibited good stability.

#### 2.11.3. Repeated-Batch Decolorization of Four-Dye Mixtures

We further investigated the repeated-batch decolorization of four-dye mixtures (azo + anthraquinone + triphenylmethane + indigo) (Figure S12). The crude laccase preparation showed high efficiency in the repeated-batch decolorization of RB5 + RBBR + MG + IC and RB5 + RB4 + MG + IC, where in RB5 + RBBR + MG + IC, the crude laccase showed efficient decolorization of all four dyes. The relative decolorization efficiencies of the azo dye RB5 in batches 2, 3, 4, 5, 6, and 7 were 93.76%, 84.55%, 82.18%, 77.25%, 72.39%, and 67.49%, respectively, while the relative decolorization efficiencies of the anthraquinone dye RBBR in batches 2, 3, 4, 5, 6, and 7 were 96.54%, 82.20%, 81.44%, 73.64%, 67.73%, and 63.56%, respectively. The relative decolorization efficiencies of the triphenylmethane dye MG in batches 2, 3, 4, 5, 6, and 7 were 96.73%, 89.48%, 85.76%, 83.66%, 82.65%, and 79.35%, respectively, and the relative decolorization efficiencies of the indigo dye IC in batches 2, 3, 4, 5, 6, and 7 were 95.98%, 88.57%, 84.67%, 79.73%, 74.68%, and 71.76%, respectively. In batch 7, the crude laccase preparation still showed efficient decolorization of the four dyes in the mixture, and the four dyes showed similar changes in relative decolorization efficiency during the seven batches (Figure S12B). In RB5 + RB4 + MG + IC, the relative decolorization efficiencies of the azo dye RB5 in batches 2, 3, 4, 5, 6, and 7 were 91.83%, 79.45%, 71.10%, 57.08%, 64.36%, and 52.94%, respectively, while the relative decolorization efficiencies of the anthraquinone dye RB4 in batches 2, 3, 4, 5, 6, and 7 were 91.53%, 78.49%, 70.78%, 59.61%, 64.23%, and 54.49%, respectively. The relative decolorization efficiencies of the triphenylmethane dye MG in batches 2, 3, 4, 5, 6, and 7 were 92.53%, 80.87%, 75.03%, 70.14%, 65.14%, and 60.33%, respectively, and the relative decolorization efficiencies of the indigo dye IC in batches 2, 3, 4, 5, 6, and 7 were 93.51%, 81.02%, 71.94%, 61.20%, 58.33%, and 56.00%, respectively (Figure S12D).

The crude laccase preparation also showed high efficiency in the repeated-batch decolorization of NC + RBBR + MG + IC and NC + RB4 + MG + IC (Figure S12A,C). Among the four dyes, the crude laccase preparation showed high efficiency in the repeated-batch decolorization of RBBR/RB4, MG, and IC, while the efficiency was relatively low for the azo dye NC. In NC + RBBR + MG + IC, the relative decolorization efficiencies of the azo dye NC in batches 2, 3, 4, 5, 6, and 7 were 82.91%, 62.41%, 53.36%, 52.49%, 51.06%, and 49.10%, respectively, while the relative decolorization efficiencies of the anthraquinone dye RBBR in batches 2, 3, 4, 5, 6, and 7 were 96.54%, 76.78%, 71.79%, 72.90%, 73.23%, and 68.01%, respectively. The relative decolorization efficiencies of the triphenylmethane dye MG in batches 2, 3, 4, 5, 6, and 7 were 96.00%, 85.91%, 83.09%, 84.75%, 85.40%, and 82.96%, respectively, while the relative decolorization efficiencies of the indigo dye IC in batches 2, 3, 4, 5, 6, and 7 were 94.29%, 88.76%, 81.27%, 79.80%, 78.58%, and 74.98%, respectively (Figure S12A). Compared to RBBR, MG, and IC, the relative decolorization efficiency of NC in the dye mixture decreased significantly with the batches.

The change in laccase activity during the repeated-batch decolorization of four-dye mixtures is shown in Figure S14G–J. As shown in Figure S14H, during the repeated-batch decolorization of RB5 + RBBR + MG + IC mixture, the relative laccase activities of the first, second, third, fourth, fifth, sixth, and seventh cycles were 61.82%, 50.98%, 42.61%, 33.25%, 28.82%, 31.53%, and 34.48%, respectively. As shown in Figure S14J, during the repeated-batch decolorization of the RB5 + RB4 + MG + IC mixture, the relative laccase activities of the first, second, third, fourth, fifth, sixth, and seventh cycles were 66.99%, 56.89%, 51.48%, 41.13%, 34.97%, 42.86%, and 43.84%, respectively. As shown in Figure S14G, during the repeated-batch decolorization of the NC + RBBR + MG + IC mixture, the relative laccase activities of the first, second, third, fourth, fifth, sixth, and seventh cycles were 62.81%, 51.72%, 40.39%, 31.28%, 26.60%, 31.53%, and 31.77%, respectively. The above results indicated that during the repeated-batch decolorization of four-dye mixtures, the laccase activity of the crude enzyme also showed strong stability.

In summary, the crude laccase preparation showed high efficiency in the repeated-batch decolorization of four-dye mixtures (azo + anthraquinone + triphenylmethane + indigo), and the crude laccase was stable in the repeated-batch degradation of dye mixtures.

### 2.12. Detoxification of Single and Mixed Dyes

#### 2.12.1. Detoxification of Single Dyes

##### Azo Dye NC

We investigated the toxicity of the untreated and decolorized azo dye NC to rice seedling shoot and root growth.

**Figure 8:**
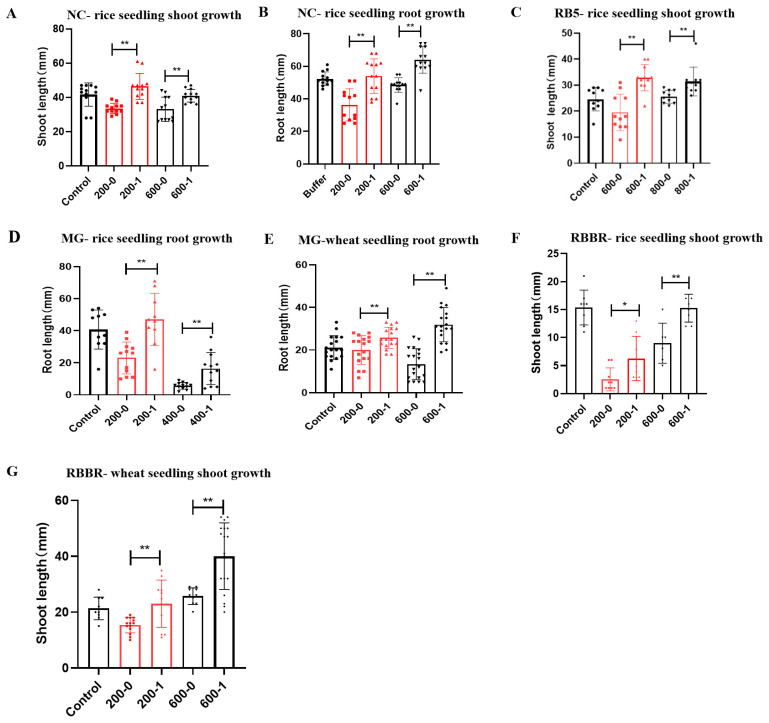
Detoxification of single dyes by treatment with the crude laccase from *P. eryngii*. (**A**) The toxicity of untreated and decolorized azo dye NC to rice seedling shoot growth. Control: acetic acid–sodium acetate buffer (pH = 5.0) added (not adding dye). 200-0: 200 mg/L untreated NC, 200-1: 200 mg/L decolorized NC, 600-0: 600 mg/L untreated NC, 600-1: 600 mg/L decolorized NC. *p* < 0.01 indicates a highly significant difference (**). (**B**) The toxicity of untreated and decolorized azo dye NC to rice seedling root growth. Control: acetic acid–asodium acetate buffer (pH = 5.0) added (not adding dye). 200-0: 200 mg/L untreated NC, 200-1: 200 mg/L decolorized NC, 600-0: 600 mg/L untreated NC, 600-1: 600 mg/L decolorized NC. *p* < 0.01 indicates a highly significant difference (**). (**C**) The toxicity of untreated and decolorized azo dye RB5 to rice seedling shoot growth. Control: acetic acid–sodium acetate buffer (pH = 5.0) added (not adding dye). 600-0: 600 mg/L untreated RB5, 600-1: 600 mg/L decolorized RB5, 800-0: 800 mg/L untreated RB5, 800-1: 800 mg/L decolorized RB5. *p* < 0.01 indicates a highly significant difference (**). (**D**) The toxicity of untreated and decolorized triphenylmethane dye MG to rice seedling root growth. Control: acetic acid–sodium acetate buffer (pH = 5.0) added (not adding dye). 200-0: 200 mg/L untreated MG, 200-1: 200 mg/L decolorized MG, 400-0: 400 mg/L untreated MG, 400-1: 400 mg/L decolorized MG. *p* < 0.01 indicates a highly significant difference (**). (**E**) The toxicity of untreated and decolorized triphenylmethane dye MG to wheat seedling root growth. Control: acetic acid–sodium acetate buffer (pH = 5.0) added (not adding dye). 200-0: 200 mg/L untreated MG, 200-1: 200 mg/L decolorized MG, 600-0: 600 mg/L untreated MG, 600-1: 400 mg/L decolorized MG. *p* < 0.01 indicates a highly significant difference (**). (**F**) The toxicity of untreated and decolorized anthraquinone dye RBBR to rice seedling shoot growth. Control: acetic acid–sodium acetate buffer (pH = 5.0) added (not adding dye). 200-0: 200 mg/L untreated RBBR, 200-1: 200 mg/L decolorized RBBR, 600-0: 600 mg/L untreated RBBR, 600-1: 600 mg/L decolorized RBBR. *p* < 0.01 indicates a highly significant difference (**). *p* < 0.05 indicates a significant difference (*). (**G**) The toxicity of untreated and decolorized anthraquinone dye RBBR to wheat seedling shoot growth. Control: acetic acid–sodium acetate buffer (pH = 5.0) added (not adding dye). 200-0: 200 mg/L untreated RBBR, 200-1: 200 mg/L decolorized RBBR, 600-0: 600 mg/L untreated RBBR, 600-1: 600 mg/L decolorized RBBR. *p* < 0.01 indicates a highly significant difference (**). NC: New Coccine; RB5: Reactive Black 5; MG: Methyl Green; RBBR: Remazol Brilliant Blue R.

As shown in Figure 8A,B, untreated NC showed strong inhibition of rice seedling shoot and root growth. The shoot lengths of the control (buffer without dye) were 1.24- and 1.26-fold those of the 200 and 600 mg/L untreated NC (200-0 and 600-0), respectively, while the root lengths of the control (buffer without dye) were 1.41- and 1.18-fold those of the 200 and 600 mg/L untreated NC (200-0 and 600-0), respectively. These results indicated that untreated NC was highly toxic to rice seedling shoot and root growth.

As shown in Figure 8A, the shoot lengths with 200 and 600 mg/L decolorized NC (200-1 and 600-1) were 1.38- and 1.24-fold those of 200 and 600 mg/L untreated NC (200-0 and 600-0), respectively, and the differences were highly significant (*p* < 0.01). Thus, the shoot growth of 200 and 600 mg/L decolorized NC was significantly better than untreated NC, and these results indicated that decolorized NC demonstrated lower toxicity to rice seedling shoot growth compared to untreated NC. Degradation with the crude laccase preparation could reduce the toxicity of the azo dye NC to rice seedling shoot growth.

As shown in Figure 8B, the root lengths of 200 and 600 mg/L decolorized NC (200-1 and 600-1) were 1.41- and 1.40-fold those of 200 and 600 mg/L untreated NC (200-0 and 600-0), respectively. These results indicated that decolorized NC demonstrated lower toxicity to rice seedling root growth compared to untreated NC. Thus, degradation with the crude laccase preparation could reduce the toxicity of azo dye NC to rice seedling root growth.

In summary, the untreated azo dye NC was highly toxic to the root and shoot growth of rice seedlings, while the toxicity of NC was significantly reduced after decolorization with the crude laccase. Decolorization with the crude laccase preparation efficiently reduced the phytotoxicity of the azo dye NC.

##### Azo Dye RB5

We subsequently investigated the toxicity of the untreated and decolorized azo dye RB5 to rice seedling shoot growth.

As shown in Figure 8C, 600 and 800 mg/L of untreated RB5 showed a strong inhibition of rice seedling shoot growth, and the shoot lengths of the control (buffer without dye) were 1.5- and 1.1-fold those of 600 and 800 mg/L untreated RB5 (600-0 and 800-0), respectively. These results indicated that untreated RB5 was highly toxic to rice seedling shoot growth.

As shown in Figure 8C, the shoot lengths of 600 and 800 mg/L decolorized NC (600-1 and 800-1) were 1.70- and 1.15-fold those of 600 and 800 mg/L untreated RB5 (600-0 and 800-0), respectively, and the differences were highly significant (*p* < 0.01). These results indicated that decolorized RB5 demonstrated a lower toxicity to rice seedling shoot growth compared to untreated RB5. Decolorization with the crude laccase preparation could efficiently reduce the toxicity of RB5 to rice seedling shoot growth, and decolorization with the crude laccase preparation efficiently reduced the phytotoxicity of the azo dye RB5.

##### Triphenylmethane Dye MG

We investigated the toxicity of the untreated and decolorized triphenylmethane dye MG to rice seedling root growth. As shown in Figure 8D, 200 and 400 mg/L untreated MG showed strong inhibition of root growth, and inhibition increased with increasing dye concentration, with untreated 400 mg/L MG almost completely inhibiting root growth. The root lengths of the control (buffer without dye) were 1.89- and 7.48-fold those of 200 and 400 mg/L untreated MG (200-0 and 400-0), respectively. These results indicated that untreated MG was highly toxic to the root growth of rice seedlings.

As shown in Figure 8D, the rice seedling root lengths of 200 and 400 mg/L decolorized MG (200-1 and 400-1) were 2.19- and 4.00-fold those of 200 and 400 mg/L untreated MG (200-0 and 400-0), respectively, and the differences were highly significant (*p* < 0.01), indicating that the root growth of 200 and 400 mg/L decolorized MG was significantly better than that of untreated MG. These results demonstrated that decolorized MG showed a much lower toxicity to rice seedling root growth compared to untreated MG. Thus, decolorization with the crude laccase preparation could efficiently reduce the toxicity of MG to rice seedling root growth.

As shown in Figure 8E, 200 and 600 mg/L untreated MG inhibited wheat seedling root growth. The root lengths of the control (buffer without dye) were 1.09- and 1.71-fold those of 200 and 600 mg/L untreated MG (200-0 and 600-0), respectively, and these results indicated that untreated MG was toxic to wheat seedling root growth.

As shown in Figure 8E, the wheat seedling root lengths of 200 and 600 mg/L decolorized MG (200-1 and 600-1) were 1.22- and 2.55-fold those of 200 and 600 mg/L untreated MG (200-0 and 600-0), and the differences were highly significant (*p* < 0.01). These results indicated that decolorized MG demonstrated a lower toxicity to wheat seedling root growth compared to untreated MG. Thus, decolorization with the crude laccase preparation could also reduce the toxicity of MG to wheat seedling root growth.

In summary, the untreated triphenylmethane dye MG was highly toxic to the root growth of rice and wheat seedlings, and the toxicity of MG was reduced by decolorization. Decolorization with the crude laccase preparation efficiently reduced the phytotoxicity of the triphenylmethane dye MG.

##### Anthraquinone Dye RBBR

We subsequently investigated the toxicity of the untreated and decolorized anthraquinone dye RBBR to rice and wheat seedling shoot growth.

As shown in Figure 8F, the rice seedling shoot lengths of 200 and 600 mg/L decolorized RBBR (200-1 and 600-1) were 2.45- and 1.69-fold those of 200 and 600 mg/L untreated RBBR (200-0 and 600-0), respectively, and the differences were highly significant (*p* < 0.01). As shown in Figure 8G, the wheat seedling shoot lengths of 200 and 600 mg/L decolorized RBBR (200-1 and 600-1) were 1.65- and 1.58-fold those of 200 and 600 mg/L untreated RBBR (200-0 and 600-0), respectively. These results indicated that the toxicity toward rice and wheat seedling shoot growth was significantly reduced with decolorized RBBR. Thus, decolorization with the crude laccase preparation efficiently reduced the phytotoxicity of the anthraquinone dye RBBR.

#### 2.12.2. Detoxification of Mixed Dyes

##### Mixed Dyes: NC (Azo) + RB5 (Azo) + RB4 (Anthraquinone) + RBBR (Anthraquinone)

We next investigated the toxicity of untreated and decolorized NC + RB5 + RB4 + RBBR to wheat seedling shoot growth.

**Figure 9:**
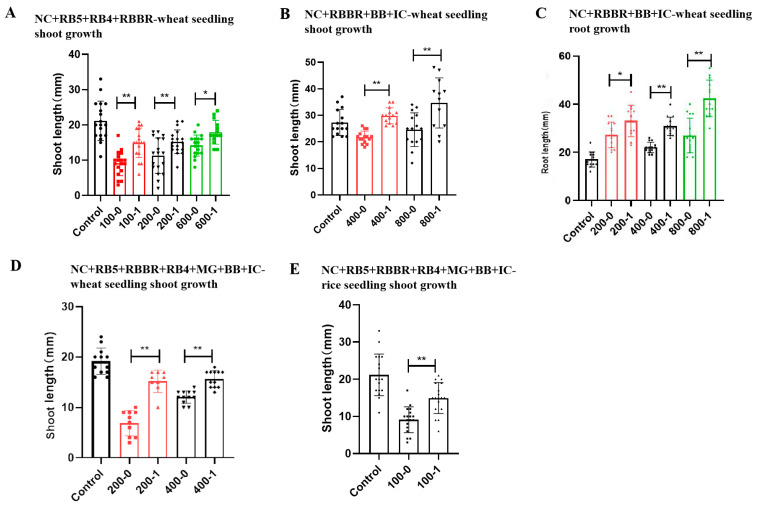
Detoxification of mixed dyes (four- and seven-dye mixture) with the treatment with the crude laccase from *P. eryngii*. (**A**) The toxicity of untreated and decolorized NC (azo) + RB5 (azo) + RB4 (anthraquinone) + RBBR (anthraquinone) to wheat seedling shoot growth. Control: acetic acid–sodium acetate buffer (pH = 5.0) added (not adding dye). 100-0: 100 mg/L untreated dye mixture (the concentration of each dye was 100 mg/L). 100-1: 100 mg/L decolorized dye mixture (the concentration of each dye was 100 mg/L). 200-0: 200 mg/L untreated dye mixture (the concentration of each dye was 200 mg/L). 200-1: 200 mg/L decolorized dye mixture (the concentration of each dye was 200 mg/L). 600-0: 600 mg/L untreated dye mixture (the concentration of each dye was 600 mg/L). 600-1: 600 mg/L decolorized dye mixture (the concentration of each dye was 600 mg/L). *p* < 0.01 indicates a highly significant difference (**). *p* < 0.05 indicates a significant difference (*). (**B**) The toxicity of untreated and decolorized NC (azo) + RBBR (anthraquinone) + BB (triphenylmethane) + IC (indigo) to wheat seedling shoot growth. Control: acetic acid–sodium acetate buffer (pH = 5.0) added (not adding dye). 400-0: 400 mg/L untreated dye mixture (the concentration of each dye was 400 mg/L). 400-1: 400 mg/L decolorized dye mixture (the concentration of each dye was 400 mg/L). *p* < 0.01 indicates a highly significant difference (**). (**C**) The toxicity of untreated and decolorized NC (azo) + RBBR (anthraquinone) + BB (triphenylmethane) + IC (indigo) to wheat seedling root growth. Control: acetic acid–sodium acetate buffer (pH = 5.0) added (not adding dye). 200-0: 200 mg/L untreated dye mixture (the concentration of each dye was 200 mg/L). 200-1: 200 mg/L decolorized dye mixture (the concentration of each dye was 200 mg/L). 400-0: 400 mg/L untreated dye mixture (the concentration of each dye was 400 mg/L). 400-1: 400 mg/L decolorized dye mixture (the concentration of each dye was 400 mg/L). 800-0: 800 mg/L untreated dye mixture (the concentration of each dye was 800 mg/L). 800-1: 800 mg/L decolorized dye mixture (the concentration of each dye was 800 mg/L). *p* < 0.01 indicates a highly significant difference (**). *p* < 0.05 indicates a significant difference (*). (**D**) The toxicity of untreated and decolorized NC (azo) + RB5 (azo) + RBBR (anthraquinone) + RB4 (anthraquinone) + MG (triphenylmethane) + BB (triphenylmethane) + IC (indigo) to wheat seedling shoot growth. Control: acetic acid–sodium acetate buffer (pH = 5.0) added (not adding dye). 200-0: 200 mg/L untreated seven-dye mixture (the concentration of each dye was 200 mg/L). 200-1: 200 mg/L decolorized seven-dye mixture (the concentration of each dye was 200 mg/L). 400-0: 400 mg/L untreated seven-dye mixture (the concentration of each dye was 400 mg/L). 400-1: 400 mg/L decolorized seven-dye mixture (the concentration of each dye was 400 mg/L). *p* < 0.01 indicates a highly significant difference (**). (**E**) The toxicity of untreated and decolorized NC (azo) + RB5 (azo) + RBBR (anthraquinone) + RB4 (anthraquinone) + MG (triphenylmethane) + BB (triphenylmethane) + IC (indigo) to rice seedling shoot growth. Control: acetic acid–sodium acetate buffer (pH = 5.0) added (not adding dye). 100-0: 100 mg/L untreated seven-dye mixture (the concentration of each dye was 100 mg/L). 100-1: 100 mg/L decolorized seven-dye mixture (the concentration of each dye was 100 mg/L). *p* < 0.01 indicates a highly significant difference (**). NC: New Coccine; RB5: Reactive Black 5; RBBR: Remazol Brilliant Blue R; RB4: Reactive Blue 4; MG: Methyl Green; BB: Bromophenol Blue; IC: Indigo Carmine.

As shown in Figure 9A, compared to the control (buffer without dye), untreated NC + RB5 + RB4 + RBBR showed significantly reduced wheat seedling shoot length, indicating that the untreated dye mixture inhibited the shoot growth of wheat seedlings. These results demonstrated that untreated NC + RB5 + RB4 + RBBR was highly toxic to wheat seedling shoot growth.

As shown in Figure 9A, the wheat seedling shoot lengths of the 100, 200, and 600 mg/L decolorized dye mixtures (100-1, 200-1, and 600-1) were 1.58-, 1.30-, and 1.18-fold those of the 100, 200, and 600 mg/L untreated dye mixtures (100-0, 200-0, and 600-0), respectively, and the differences were highly significant (*p* < 0.01). These results indicated that the toxicity of NC + RB5 + RB4 + RBBR was significantly reduced by decolorization. Thus, decolorization with the crude laccase preparation efficiently reduced the toxicity of the mixed dyes NC + RB5 + RB4 + RBBR to wheat seedling shoot growth.

##### Mixed Dyes: NC (Azo) + RBBR (Anthraquinone) + BB (Triphenylmethane) + IC (Indigo)

As shown in Figure 9B, the wheat seedling shoot lengths of 400 and 800 mg/L decolorized NC + RBBR + BB + IC (400-1 and 800-1) were 1.96- and 1.47-fold those of 400 and 800 mg/L untreated NC + RBBR + BB + IC (400-0 and 800-0), respectively. These results indicated that decolorized NC + RBBR + BB + IC demonstrated significantly a lower toxicity to wheat seedling shoot growth compared to the untreated dye mixture. Thus, decolorization with the crude laccase preparation could efficiently reduce the toxicity of the mixed dyes NC + RBBR + BB + IC to wheat seedling shoot growth.

As shown in Figure 9C, the wheat seedling root lengths of 400 and 800 mg/L decolorized NC + RBBR + BB + IC (400-1 and 800-1) were 1.37- and 1.12-foldthose of 400 and 800 mg/L untreated NC + RBBR + BB + IC (400-0 and 800-0), respectively, and the differences were highly significant (*p* < 0.01). These results indicated that decolorized NC + RBBR + BB + IC demonstrated a lower toxicity to wheat seedling root growth compared to the untreated dye mixture. Thus, decolorization with the crude laccase preparation could also reduce the toxicity of the mixed dyes NC + RBBR + BB + IC to wheat seedling root growth.

##### Mixed Dyes: NC (Azo) + RB5 (Azo) + RBBR (Anthraquinone) + RB4 (Anthraquinone) + MG (Triphenylmethane) + BB (Triphenylmethane) + IC (Indigo)

We further investigated the phytotoxicity of the untreated and decolorized seven-dye mixture. As shown in Figure 9D, compared to the control (buffer without dye), the shoot growth of the wheat seedlings decreased significantly with untreated NC + RB5 + RBBR + RB4 + MG + BB + IC, and the untreated seven-dye mixture could inhibit wheat seedling shoot growth. These results indicated that untreated NC + RB5 + RBBR + RB4 + MG + BB + IC was highly toxic to wheat seedling shoot growth.

As shown in Figure 9D, the wheat seedling shoot lengths of 200 and 400 mg/L decolorized NC + RB5 + RBBR + RB4 + MG + BB + IC (200-1 and 400-1) were 1.73- and 1.20-fold those of 200 and 400 mg/L untreated NC + RB5 + RBBR + RB4 + MG + BB + IC (200-0 and 400-0), respectively, and the differences were highly significant (*p* < 0.01). These results indicated that decolorized NC + RB5 + RBBR + RB4 + MG + BB + IC demonstrated a reduced toxicity to wheat seedling shoot growth compared to the untreated dye mixture. Thus, decolorization with the crude laccase preparation could reduce the toxicity of the seven-dye mixture NC + RB5 + RBBR + RB4 + MG + BB + IC to wheat seedling shoot growth.

As shown in Figure 9E, the rice seedling shoot length of 100 mg/L decolorized NC + RB5 + RBBR + RB4 + MG + BB + IC (100-1) was 1.32-fold that of the 100 mg/L untreated NC + RB5 + RBBR + RB4 + MG + BB + IC (100-0), and the differences were highly significant (*p* < 0.01). These results indicated that decolorization with the crude laccase preparation efficiently reduced the toxicity of the seven-dye mixture to the shoot growth of rice seedlings.

In summary, decolorization with the crude laccase preparation could efficiently reduce the phytotoxicity of the dye mixtures NC + RB5 + RB4 + RBBR, NC + RBBR + BB + IC, and NC + RB5 + RBBR + RB4 + MG + BB + IC.

## 3. Discussion

### 3.1. Comparison of Crude Laccase from Pleurotus eryngii (Used in This Study) with Other Crude Laccases Reported Previously in Decolorization of Different Dyes

We compared the differences in degradation of the azo dyes between the crude laccase from *Pleurotus eryngii* and other laccases reported in previous studies. Enayatzamir et al. used a crude laccase preparation (500 U/L) from *T*. *pubescens* to decolorize 60 mg/L of RB5 at pH 4.5 and room temperature and achieved a 24 h decolorization efficiency of 22.7% [47]. Zeng et al. used a crude laccase preparation from *T*. *trogii* to decolorize 18.3 mg/L of RB5 at 30 °C and pH 4.0, and the decolorization efficiency reached 65.4% at 30 min [36]. In this study, the crude laccase from *Pleurotus eryngii* (1 U/mL) was used to degrade 100 mg/L of RB5 at 30 °C, and the 24 h decolorization efficiency reached 72.58%. Even when the concentration of RB5 was increased to 800 mg/L, the 24 h decolorization efficiency was still 50.82%. Compared to previously reported crude laccase preparations [36,47], the crude laccase from *Pleurotus eryngii* achieved rapid and efficient decolorization of higher azo dye RB5 concentrations with an equal (or lesser) enzyme dosage. Compared to previously reported crude laccase preparations, the crude laccase from *Pleurotus eryngii* was more efficient for the decolorization of the azo dye RB5, suggesting that the crude laccase from *Pleurotus eryngii* offers significant potential in the decolorization and degradation of azo dye pollutants.

We also compared the similarities and differences of the crude laccase from *Pleurotus eryngii* and other laccases reported in previous studies, specifically regarding the degradation of the anthraquinone dye RBBR. Zeng et al. used 2 U/mL of a crude laccase preparation from *T*. *trogii* to degrade 50 mg/L of the anthraquinone dye RBBR, and the 6-day decolorization efficiency was 89.4 ± 2.9% [31]. In contrast, 1 U/mL of crude laccase from *Pleurotus eryngii* showed a 24 h decolorization efficiency of 98.73% for 100 mg/L of RBBR, and the dye was almost completely decolorized. Both the crude laccase preparation derived from *T*. *trogii* and the crude laccase from *Pleurotus eryngii* showed efficient RBBR decolorization. However, compared to the crude laccase preparation from *T*. *trogii* [31], the crude laccase from *Pleurotus eryngii* achieved near-complete decolorization (98.73%) with a higher concentration (100 mg/L) of RBBR in a shorter period (24 h) with a lower dosage (1 U/mL). Our results also demonstrated that even when the concentration of RBBR increased to 800 mg/L, the 24 h decolorization efficiency remained at 90.25%. The above comparisons indicated that the crude laccase from *Pleurotus eryngii* was more efficient in the decolorization of the anthraquinone dye RBBR compared to the crude laccase preparation from *T*. *trogii* [31]. For the decolorization of the anthraquinone dye RBBR at high concentrations, the crude laccase from *Pleurotus eryngii* was superior to the crude laccase prepared from *T*. *trogii* [31].

For the decolorization of triphenylmethane dyes, Darvishi et al. used a 50 U/L crude laccase preparation from *Yarrowia lipolytica* to decolorize 33.33 mg/L BB, and the 24 h decolorization efficiency was 50%, while the 24 h decolorization efficiency was 35% for MG [48]. Hadibarata et al. showed that a crude laccase preparation from *Armillaria* sp. F022 was inefficient in the degradation of brilliant green, and the 96 h decolorization efficiency was less than 40% [43]. In our study, 1 U/mL crude laccase from *Pleurotus eryngii* demonstrated 24 h decolorization efficiencies of 99.50% and 85.79% for 100 mg/L BB and MG, respectively, while the 24 h decolorization efficiencies of higher concentrations of BB and MG (800 mg/L) remained at 99.02% and 84.04%, respectively, maintaining a high level of decolorization. These comparisons showed that compared to the *Y*. *lipolytica* crude laccase preparation [48], the crude laccase from *Pleurotus eryngii* was more efficient in the decolorization of the triphenylmethane dyes BB and MG, even at very high concentrations.

For the decolorization of the indigo dye IC, Domınguez et al. achieved nearly 100% decolorization of 150 μM IC after 24 h using 800 U/L crude laccase prepared from *T*. *hirsuta* [49]. Grassi et al. also used a crude laccase preparation from *T*. *hirsuta* to achieve 96% decolorization efficiency of IC after 24 h. These results suggested excellent decolorization of IC with crude laccase preparations [41]. In our study, the 24 h decolorization efficiencies of 50, 100, and 200 mg/L IC were 94.81%, 94.93%, and 97.24%, respectively, using 1 U/mL crude laccase from *Pleurotus eryngii*. These results suggested that the crude laccase from *Pleurotus eryngii* was very efficient for IC decolorization, which was consistent with the results of previous studies. However, previous studies did not document the decolorization efficiency of IC at higher concentrations with crude laccase preparations. Our study showed that the crude laccase from *Pleurotus eryngii* was very efficient in the decolorization of IC at high levels, and the 24 h decolorization efficiencies of 400 and 800 mg/L IC were 99.13% and 96.98%, respectively, with the dye nearly completely decolorized.

### 3.2. Possible Reasons for the Different Decolorization Efficiencies of Azo Dyes

Our results showed differences in the decolorization of azo dyes with the crude laccase from *Pleurotus eryngii*, with the highest decolorization efficiency achieved for NC and relatively low efficiency for RO16, DR81, and RBV5R. This could be explained by the changes in structures of the azo dyes via the laccase degradation of chromophores, where an electron was extracted from the phenolic/naphthol ring to produce a phenoxy radical. Then, a second electron was extracted to generate an aromatic cation, which was subjected to nucleophilic attack by water, resulting in the breakdown of the dye molecule [50,51]. The formation of aromatic cations could be promoted by the electron-donating group on the aromatic ring. For azo dyes carrying strong electron-donating hydroxyl groups in the ortho and para positions of the azo bond, the best performance of biochemical decolorization would be achieved. Conversely, the presence of electron-accepting groups on the aromatic ring, such as halogen and nitro, would impede the formation of radical cations, consequently leading to inefficient dye degradation [52].

The chemical structure of a dye will affect its decolorization efficiency with laccase, i.e., decolorization will be affected by the molecular structure of the dye. Compared to dyes with substantial substitutions and high molecular weights, dyes with simple structures and low molecular weights will be amenable to decolorization [53]. The high decolorization efficiency of NC relative to RO16, DR81, and RBV5R could be attributed to its simple structure and low molecular weight.

Differences in the electron distribution, charge density, and steric hindrance of the dyes will significantly influence the decolorization efficacy and reaction rate [53,54]. The resistance (recalcitrance) of RBV5R to decolorization could be attributed to the presence of stable aromatic rings connected to the azo group.

### 3.3. Possible Reasons for the Different Decolorization Efficiencies of Triphenylmethane Dyes

Our results showed that the decolorization efficiency of the crude laccase from *Pleurotus eryngii* varied for triphenylmethane dyes with different structures, where the decolorization efficiencies of BB and MG were the highest, while the decolorization efficiency of AF was very low. This could be explained by the central carbon atom of triphenylmethane, which was chemically active and susceptible to replacement by metal atoms. Nevertheless, the arrangement of the three benzene rings in a non-coplanar, three-bladed propeller structure introduced substantial steric hindrance. This hindrance prevented the catalytic center of laccase from accessing the central methine group, thus inhibiting the reaction. Additionally, it was not feasible to achieve simultaneous electron delocalization across all three benzene rings, and electron delocalization with a benzene ring would only occur when the sp^2^ hybrid orbital of the carbon anion was appropriately aligned with one of the aromatic systems. This arrangement reduced the catalytic decolorization efficiency of triphenylmethane dyes [55]. The substrate structure serves as a key factor affecting degradation efficiency, and in MG and BB, simple groups such as -NH_2_ or -OH were directly attached to the aromatic rings, consisting of electron-donating groups that can facilitate substrate degradation. Nevertheless, the high molecular weight of AF rendered it less amenable to binding with laccase, potentially accounting for its low decolorization efficiency.

### 3.4. Explanation of How the Decolorization Activity of Laccase Was Higher at Higher Concentrations of Dyes 

Our results suggested that the decolorization activity of laccase was higher at higher concentrations of dyes. A possible explanation for this phenomenon is as follows. The intermediate products of laccase degradation of some dyes can serve as mediators for laccase reactions. Mediators can improve the catalytic reaction ability of laccase and ultimately improve the decolorization efficiency of dyes. Therefore, the higher the dye concentration, the higher the level of mediator produced by the reaction, leading to a higher decolorization activity of laccase and stronger dye decolorization.

### 3.5. Crude Laccase from Pleurotus eryngii Had a High Tolerance to Various Metal Salts and Organic Solvents in the Degradation of Dyes

Dye wastewater originating from the textile industry typically contains high levels of metal salts, which can affect the degradation process by modulating various factors associated with biodegradation. Consequently, the bioremediation of industrial dye wastewater must consider the inhibitory effect of metal salts on the degradation process [56].

Previous studies have predominantly investigated the effects of low concentrations of metal ions on the degradation of dyes with laccase [57,58]. In this study, we systematically investigated the impacts of metal ions at high concentrations on the degradation of azo, anthraquinone, triphenylmethane, and indigo dyes through crude laccase from *Pleurotus eryngii*. Our results showed that the crude laccase was tolerant to high concentrations of SO_4_^2−^ salts such as 800 mM MnSO_4_, MgSO_4_, ZnSO_4_, K_2_SO_4_, and Na_2_SO_4_ during the decolorization of different types of dyes. Even at elevated concentrations, these salts did not exert significant impacts on the degradation of dyes. The crude laccase from *Pleurotus eryngii* demonstrated a high tolerance to a variety of SO_4_^2−^ salts, even at extremely high concentrations, in the degradation of the triphenylmethane dye BB. These findings underscored the advantages of crude laccase from *Pleurotus eryngii* in the decolorization of dyes. This crude laccase preparation may hold great potential for the efficient treatment of dye wastewater containing metal ions at high concentrations.

### 3.6. Decolorization Kinetics of Mixed Dyes

The kinetics of azo + anthraquinone dye decolorization showed that for NC + RB4, the *k* value of the azo dye NC increased from $1.84\;\times \;10^{-2}$ min^−1^ in the control (single-dye decolorization) to 2.00 $\;\times \;10^{-2}$ min^−1^ in the mixture, while for NC + RBBR, the *k* value of the azo dye NC increased from $1.84\;\times \;10^{-2}$ min^−1^ in the control (single-dye decolorization) to 2.84$\;\times \;10^{-2}$ min^−1^ in the mixture. These results demonstrated that the presence of an anthraquinone dye significantly increased the reaction rate of the azo dye, where an anthraquinone dye in the mixture promoted the decolorization rate of the azo dye. A potential explanation for this phenomenon is as follows. Zeng et al. used a *T*. *trogii* crude laccase preparation to decolorize azo and anthraquinone dyes without exogenous redox mediators. The study found that the enzyme could decolorize azo dyes only in the presence of a mediator, such as 20 μM anthraquinone dye, suggesting that anthraquinone dyes played a mediating role in azo dye degradation [31]. This was also consistent with our results. During the degradation of mixed azo and anthraquinone dyes with crude laccase from *Pleurotus eryngii*, the anthraquinone dye promoted the decolorization of the azo dye (increased the decolorization rate of the azo dye). The anthraquinone dye appeared to act as a mediator rather than a catalyst, as it was gradually degraded. Notably, the degradation rate of the azo dye significantly decreased when the concentration of the anthraquinone dye was reduced to zero. This phenomenon could be attributed to the enzymatic oxidation of the dye molecule (M) (anthraquinone dye), which was a substrate of laccase, forming a radical cation (M). This reactive and unstable radical cation returned to its original state (M) via spontaneous degradation, generating enzymatic oxidation products (P^+^_M_) or the oxidization of non-substrate dye molecules (D) (azo dye). The oxidized non-substrate dye molecules would be unstable and undergo degradation, yielding the final product (P_D_). The electrons extracted by laccase would then be transferred to molecular oxygen, resulting in the formation of water. According to this reaction mechanism, dye molecules functioning as substrates for laccase could potentially act as mediators, facilitating the degradation of dyes that were not substrates for laccase.

The decolorization kinetics of the azo + indigo dyes (NC + IC) showed that the azo dye in the mixture increased the decolorization rate of the indigo dye; however, the indigo dye inhibited the decolorization rate of the azo dye. This was because the indigo dye was not a direct substrate of laccase, and low-molecular-weight mediators would be added in the degradation of indigo dye, forming highly active and relatively stable intermediates under the action of laccase. These active intermediates could transfer electrons from molecular oxygen to the indigo dye to facilitate its degradation [59]. In a mixture of the azo dye NC and the indigo dye IC, NC could function as a low-molecular-weight mediator and promote the decolorization of IC due to its simple structure and the ease of binding to the active center of laccase. When an indigo dye is mixed with an azo dye, polymerization may occur, impeding the binding of the azo dye to the active center of laccase, subsequently reducing the rate of laccase-mediated decolorization of the azo dye.

The decolorization kinetics of the azo + triphenylmethane dyes (NC + MG) showed that the azo dye NC in the mixture promoted the decolorization rate of the triphenylmethane dye MG; however, the triphenylmethane dye inhibited the decolorization of the azo dye. A possible explanation for this phenomenon is as follows. Upon mixing of the azo dye NC and triphenylmethane dye MG, non-covalent interactions, such as van der Waals forces, hydrogen bonds, or π-π stacking, possibly facilitated their association. This association could lead to the formation of complexes or aggregates, complicating the structure of NC and reducing its binding to the active center of laccase, thus reducing the decolorization rate of NC. When NC was bound to the active center of laccase, it could induce conformational changes in the enzyme. These changes could expose the active center and facilitate the association of MG with laccase, thus increasing the decolorization rate of MG.

The kinetics of decolorization of anthraquinone + indigo dyes (RB4 + IC) showed that the anthraquinone dye RB4 greatly increased the decolorization rate of the indigo dye IC. A potential explanation for this phenomenon is as follows. Studies have shown that an anthraquinone dye capable of electron transfer can be directly degraded as a substrate of laccase [60]. Furthermore, when laccase and a mediator combine to create a laccase–mediator system, the decolorization and degradation of non-laccase substrates, such as indigo dye, could be significantly enhanced. When RB4 was mixed with IC, RB4 could act as a redox mediator to promote the decolorization of IC through the crude laccase preparation.

The decolorization kinetics of indigo + triphenylmethane dyes (IC + CR) showed that the triphenylmethane dye CR greatly promoted the decolorization rate of the indigo dye IC, but the indigo dye inhibited the decolorization rate of the triphenylmethane dye. This phenomenon could be explained by the following mechanism. In the process of laccase-mediated degradation of CR, electron donation through the hydroxyl group within CR led to the formation of a free radical. This free radical acted as an activator to facilitate the laccase-mediated degradation of IC. When laccase was engaged in the degradation of a mixture of indigo dye and triphenylmethane dye, the presence of both dyes collectively increased the overall dye concentration. Therefore, the degradation rate of the triphenylmethane dye in the mixture was significantly reduced due to the limited availability of laccase to target individual dye molecules.

The decolorization kinetics of anthraquinone + triphenylmethane dyes (RBBR + CR) showed that the anthraquinone dye RBBR promoted the decolorization rate of the triphenylmethane dye CR. A possible explanation for this phenomenon is as follows. Similar to the promotion of indigo dye decolorization by anthraquinone dye, the anthraquinone dye could also serve as a mediator in the laccase-mediated degradation of the triphenylmethane dye through electron transfer. When RBBR was mixed with CR, RBBR underwent reduction by the electrons from the medium and promptly transferred these electrons to CR to enhance its decolorization rate.

The decolorization kinetics of triphenylmethane + triphenylmethane dyes (MG + BB, MG + CR, and BB + AF) showed that the decolorization rate of one triphenylmethane dye significantly improved, while the decolorization rate of the other triphenylmethane dye was reduced. A possible explanation is as follows. The generally low degradation efficiency of triphenylmethane dyes with laccase could be attributed to the substantial size of triphenylmethane, which possibly impeded its access to the active center of laccase [61]. When a triphenylmethane dye (A) with a relatively simple structure is bound to the active center of laccase, it could induce a conformational change in the enzyme, facilitating the subsequent binding of another triphenylmethane dye (B) with a more complex structure, thus improving the decolorization rate of B. However, competition for laccase between dyes B and A results in inhibited decolorization of dye A.

The decolorization kinetics of azo + azo dyes (NC + RB5) showed that the decolorization rate of both NC and RB5 in the mixture was reduced compared to the single dyes. Possible explanations for this phenomenon are elucidated as follows. In the degradation of NC + RB5, the competitive binding of two structurally similar azo dyes to the active center of laccase diminished the decolorization rate and reduced the *k* values of both dyes. In addition, Andrea et al. identified the generation of phenolic compounds and an abundance of polymerization products during the laccase-mediated degradation of azo dyes. These products contributed to maintaining the structural integrity of azo groups and participated in connecting bound and unbound dyes [62]. The formation of these polymerization products constrained the mobility of dye molecules, subsequently limiting their accessibility to laccase and slowing down the decolorization process.

### 3.7. Possible Reasons for the Decrease in Toxicity of Dyes after Decolorization

Our results suggested that decolorization with the crude laccase preparation efficiently reduced the phytotoxicity of different dyes. The possible mechanism for reducing the toxicity of dyes to plants is as follows. Our results indicated that the untreated dye was highly toxic to plant seedling shoot and root growth. Dyes that have not been decolorized had strong toxicity to the germination and growth of plant seeds. Untreated dyes can have negative effects on various physiological functions of plant seeds. For example, when plant seeds absorb a large amount of azo dyes, the azo dyes will firstly be converted into corresponding aromatic amines through the reduction reaction of -N=N- bonds, and finally form N+ ions through the activation mechanism of aromatic amines, which can bind to the electronegative centers of biological macromolecules (such as N and O atoms in DNA bases and -SH and -OH groups in protein molecules). This can lead to severe inhibition of normal physiological functions such as respiration and nutrient transport in plant seeds. Therefore, untreated dyes had strong toxicity to the germination and growth of plant seeds.

The inhibitory effect of dyes decolorized with laccase on the normal physiological functions of plant seeds, such as respiration and nutrient transport, was weakened, thereby reducing the toxicity of dyes to plant seedling shoot and root growth.

### 3.8. Advantages and Practical Application Value of Crude Laccase Preparation for Decolorization and Detoxification of Dye Pollutants

Our findings highlighted the substantial potential of crude laccase from *Pleurotus eryngii* for the efficient decolorization and detoxification of both single and mixed dyes with varying structures. The use of a crude laccase preparation for the decolorization and detoxification of dye pollutants had the following advantages and practical applications. Zeng et al. showed that the degradation efficiency of a crude laccase preparation was higher than that of purified laccase, which could be explained by the fact that fungi secrete laccases along with redox mediators to facilitate the decolorization of dyes [36]. A crude laccase preparation can efficiently decolorize different types of dyes without requiring additional mediators, as these molecules will be inherently present in the crude laccase preparation. Therefore, the use of a crude laccase preparation can substantially reduce the overall cost of laccase-mediated dye-pollutant degradation. Furthermore, compared to the purification of laccase, the preparation of crude laccase offers a simple process that eliminates the need for a series of complex purification steps. This approach will eliminate the requirement for methods and materials for laccase separation and purification. Consequently, the costs associated with using laccase in pollutant treatment will be greatly reduced. Our results showed that the crude laccase from *Pleurotus eryngii* could efficiently decolorize and detoxify single and mixed dyes, greatly enhancing the feasibility and practicality of large-scale laccase applications in dye-pollutant treatment. In summary, the direct use of a crude laccase preparation for dye wastewater treatment yielded satisfactory results while substantially reducing the overall cost. This approach offers significant promise for the practical application of laccase in pollutant bioremediation.

Furthermore, our investigation revealed the efficacy of crude laccase from *Pleurotus eryngii* in the repeated-batch decolorization of single and mixed dyes, such as azo + triphenylmethane dyes (RB5 + BB) and azo + indigo dyes (NC + IC and RB5 + IC). The crude laccase from *Pleurotus eryngii* had a strong continuous batch decolorization ability for single and mixed dyes. In the practical application of laccase for the efficient treatment of environmental pollutants and the reusability and stability of laccase serve as pivotal factors in cost reduction for industrial applications. In this work, we systematically investigated the repeated-batch decolorization of single and mixed dyes with crude laccase from *Pleurotus eryngii*. After several batches, the crude laccase preparation retained its decolorization activity for both single and mixed dyes. Our results showed that the crude laccase from *Pleurotus eryngii* was stable and reusable in repeated-batch degradation of dyes. These characteristics significantly reduced the cost of crude laccase from *Pleurotus eryngii* for practical application, markedly enhancing its value in real-world applications. This remarkable attribute distinguished the use of crude laccase from *Pleurotus eryngii* as a cost-effective and highly feasible solution for the efficient treatment of dye pollutants.

## 4. Materials and Methods

### 4.1. Strain and Culture Media

*Pleurotus eryngii* ACCC 50757 was obtained from the Agricultural Culture Collection of China (ACCC) and a GYP medium was used for laccase production (20 g/L of glucose, 5 g/L of yeast extract, 5 g/L of peptone, 1 g/L of MgSO_4_·7H_2_O, and 0.002 g/L of CuSO_4_·5H_2_O).

### 4.2. Induction of Laccase Production and Preparation of Crude Laccase

*Pleurotus eryngii* was inoculated onto a PDA medium and incubated at 28 °C for 7 days. Ten mycelial plugs were then collected using a puncher and transferred with an inoculation needle into the PDB medium. The inoculated medium was allowed to stand for 12 h and then incubated at 28 °C and 180 r/min for 7 days to obtain uniform mycelial pellets.

The mycelial pellets (5 mL) were transferred from PDB to GYP medium (100 mL) and incubated at 28 °C and 180 r/min for 3 days. Then, CuSO_4_ at a final concentration of 2 mM was added to induce laccase production, and incubation was continued at 28 °C and 180 r/min. Samples were collected every day to determine the laccase activity. The culture broth was collected under maximum laccase activity and filtered through eight layers of gauze to remove the mycelia, with the filtrate stored at −20 °C overnight. After thawing at room temperature, the polysaccharides were removed with precipitation and filtration to obtain the crude laccase preparation. The polysaccharides precipitated included glucan, mannan, etc. Laccase activity was determined with 2,2′-azino-bis(3-ethylbenzothiazoline-6-sulfonic acid) (ABTS) as the substrate. One unit of enzyme activity was defined as the amount of enzyme required to oxidize 1 μmol of ABTS per min.

### 4.3. Decolorization of the Dyes with Crude Laccase from Pleurotus eryngii and Calculation of the Decolorization Efficiency

Table 3 presents some information on the dyes used in this study, including azo dyes, anthraquinone dyes, triphenylmethane dyes, and indigo dye.

The 1 mL decolorization system included the crude laccase preparation (1 U/mL), dyes at final concentrations of 50, 100, 200, 400, and 800 mg/L (diluted from 5 g/L stock solutions), and 100 mM acetic acid–sodium acetate buffer (pH = 5.0). The acetic acid–sodium acetate buffer (pH 5.0) was used to dissolve the stock solution of dyes. The mixture was thoroughly mixed and then incubated in a water bath at 30 °C for 24 h in the dark. The change in the maximum absorbance value before and after 24 h of dye degradation was determined using a UV-visible spectrophotometer. The decolorization efficiency was calculated as follows:

decolorization efficiency (%) = (*A*_0_ − *A_t_*)/*A*_0_ × 100%,

where *A*_0_ is the initial absorbance and *A_t_* denotes the absorbance after 24 h of decolorization.

### 4.4. Time Courses of Decolorization of Dyes at Different Concentrations

The 1 mL decolorization system consisted of the crude laccase preparation (1 U/mL), dyes at final concentrations of 50, 100, 200, 400, and 800 mg/L (diluted from 5 g/L stock solutions), and 100 mM acetic acid–sodium acetate buffer (pH = 5.0). The mixture was thoroughly mixed and incubated in a water bath at 30 °C in the dark, with samples collected at 0, 0.5, 1, 2, 3, 6, 9, 12, and 24 h. The absorption peaks were observed using a UV-visible spectrophotometer and the absorbance at the maximum absorption wavelength was measured. The decolorization efficiency was calculated as described in Section 4.3, and the time course of dye decolorization was plotted according to the decolorization efficiency at different time points.

### 4.5. Kinetic Equations of Single-Dye Degradation

The 5 g/L stock solution was diluted to 10, 20, 50, 80, 100, 120, 160, and 200 mg/L. The absorbance *A* at the maximum absorption wavelength of the standard dye solution was determined, and the *A*-*c* curve was plotted. The absorbance at the maximum absorption wavelength of each concentration of dye standards at 10, 20, 50, 80, 100, 120, 160, and 200 mg/L was measured using a UV-visible spectrophotometer. A straight line is plotted for the dye concentration c against absorbance A, and the slope *K* of the line is the molar absorption coefficient of each dye. The values of the coefficients are shown in Table S1. With a molar absorption coefficient *K* of the dye, the dye concentration at time *t* (*C_t_*) of decolorization can be calculated from the time-course curve. For the first-order reaction, the integral form of the reaction rate equation can be expressed by

lnCtC0=−kt

where *C*_0_ is the initial dye concentration, *C_t_* is the dye concentration at time *t*, and *k* denotes the reaction rate constant (min^−1^), which directly reflects the rate of reaction. The ln *c*-*t* curve was plotted with *t* on the horizontal axis and lnCtC0 on the vertical axis, and *k* was subsequently calculated.

### 4.6. Decolorization of Mixed Dyes: Determination of Decolorization Efficiency of Each Dye in the Mixtures

The 1 mL dye mixture decolorization system included 1 U/mL of the crude laccase preparation, two-dye mixtures (each with final concentrations of 50, 100, 200, 400, and 800 mg/L), and 100 mM acetic acid–sodium acetate buffer (pH = 5.0). The reaction system was thoroughly mixed and incubated in a water bath at 30 °C for 24 h in the dark. The absorption peaks were observed using a UV-visible spectrophotometer and the absorbance at the maximum absorption wavelength was measured to calculate the decolorization efficiency of each dye, as described in Section 4.3.

### 4.7. Decolorization Kinetics of the Mixed Dyes

During the degradation of the dyes, samples were collected at 0, 10, 20, 30, 40, 50, 60, 90, 120, and 180 min. The absorbance at the maximum absorption wavelength was measured using a UV-visible spectrophotometer, and the ln *c*-*t* curve was plotted, with the *k* value calculated as described in Section 4.5.

### 4.8. Effects of Metal Salts at Different Concentrations on Decolorization

The decolorization system of the experimental group included 1 U/mL of the crude laccase preparation, 100 mg/L of dye, metal salts (MnSO_4_, MnCl_2_, MgSO_4_, MgCl_2_, ZnSO_4_, ZnCl_2_, K_2_SO_4_, KCl, CuSO_4_, CuCl_2_, Na_2_SO_4_, NaCl, Al_2_(SO_4_)_3_, AlCl_3_, CdSO_4_, CdCl_2_, CaCl_2_, CoCl_2_, NiCl_2_, and LiCl at final concentrations of 50, 100, 200, 400, and 800 mM), and 100 mM of acetic acid–sodium acetate buffer (pH = 5.0). The control group consisted of a 1 mL decolorization system without metal salts. The reaction system was thoroughly mixed and incubated in a water bath at 30 °C for 24 h in the dark, and the absorbance of each dye at the maximum absorption wavelength was measured at 0 and 24 h. The decolorization efficiency was calculated as described in Section 4.3.

### 4.9. Effects of Organic Solvents at Different Concentrations on Decolorization

The decolorization system of the experimental group included 1 U/mL of the crude laccase preparation, 100 mg/L of dye, organic solvents (glycerol, butanediol, propanediol, ethylene glycol, methanol, ethanol, isopropanol, acetonitrile, acetone, DMSO, and DMF at final concentrations of 10%, 20%, 40%, 50%, 70%, and 80%, *v*/*v*), and 100 mM acetic acid–sodium acetate buffer (pH = 5.0). The control group consisted of a 1 mL decolorization reaction system without organic solvents, and the reaction system was thoroughly mixed and incubated in a water bath at 30 °C for 24 h in the dark. The absorbance of each dye at the maximum absorption wavelength was measured at 0 and 24 h, and the decolorization efficiency was calculated as described in Section 4.3.

### 4.10. Repeated-Batch Decolorization

#### 4.10.1. Repeated-Batch Decolorization of Single Dyes

The decolorization system of the experimental group included 100 mg/L of dye and 100 mM of acetic acid–sodium acetate buffer (pH = 5.0). The reaction system was thoroughly mixed and the initial absorbance *A*_01_ was measured. Subsequently, 0.5 U/mL of the crude laccase preparation was added and thoroughly mixed. The reaction system was incubated in a water bath at 30 °C for 24 h in the dark, the absorbance *A_t_*_1_ was measured, and the 24 h decolorization efficiency of the first-cycle reaction was calculated as described in Section 4.3. The decolorization efficiency of the first batch was set to 100% for calculation of the relative decolorization efficiency of subsequent batches. After the first decolorization batch, 100 mg/L of the same dye was added to the decolorization system and the absorbance *A*_02_ was measured. The reaction system was thoroughly mixed and incubated at 30 °C for 24 h. Then, the absorbance *A_t_*_2_ was measured to calculate the relative decolorization efficiency. Seven consecutive batches of decolorization were carried out in this study.

#### 4.10.2. Repeated-Batch Decolorization of Mixed Dyes

The decolorization system of the experimental group included mixed dyes (each with a final concentration of 100 mg/L) and 100 mM acetic acid–sodium acetate buffer (pH = 5.0). The mixture was thoroughly mixed and the initial absorbance *A*_01_ of each dye was measured. The remaining steps were the same as in Section 4.10.1.

### 4.11. Detoxification of the Dyes with Crude Laccase Preparation

#### 4.11.1. Detoxification of Single Dyes

Twenty wheat or rice seeds of the same size were selected and placed in a Petri dish. Then, 5 mL reaction systems for the experimental group, which included single dyes (final concentrations of 200, 400, 600, and 800 mg/L), 0.5 U/mL of crude laccase preparation, and 100 mM of acetic acid–sodium acetate buffer (pH = 5.0), were obtained. Laccase was not added to the control group. The mixture was then incubated in a water bath at 30 °C for 24 h in the dark and then added to the Petri dish with the seeds. The seeds were incubated at 28 °C for 3–5 days and the lengths of the shoots and roots were measured. The control acetic acid–sodium acetate buffer (pH = 5.0) (not adding dye). Differences in root and shoot length between the experimental groups (dyes with decolorization) and control group (dyes without decolorization) were analyzed using GraphPad Prism 8 software, with *p* < 0.01 indicating a highly significant difference (**) and *p* < 0.05 a significant difference (*).

#### 4.11.2. Detoxification of the Mixed Dyes

Twenty wheat or rice seeds of the same size were selected and placed in a petri dish. The 5 mL reaction system of the experimental group included mixed dyes (each at final concentrations of 200, 400, 600, and 800 mg/L), 0.5 U/mL of crude laccase preparation, and 100 mM of acetic acid–sodium acetate buffer (pH = 5.0). The remaining steps were the same as in Section 4.11.1.

### 4.12. Statistical Analysis

All measurements were repeated three times, with the data expressed as mean ± standard deviation. The significance of differences between the mean values was determined using a *t* test or analysis of variance. All data were analyzed using GraphPad Prism 8 software. Differences in the mean values between groups were assessed using the *t* test, with *p* < 0.01 indicating a highly significant difference (**) and *p* < 0.05 a significant difference (*).

## 5. Conclusions

In this study, we prepared a crude laccase from a fungal strain *Pleurotus eryngii* with high laccase production. On this basis, we systematically investigated the decolorization efficacy and reaction rates of single dyes in four structural classes. The crude laccase from *Pleurotus eryngii* showed efficient decolorization of single dyes (azo, anthraquinone, triphenylmethane, and indigo dyes). We also investigated the tolerance of the crude laccase from *P. eryngii* to metal salts and organic solvents at different concentrations during the degradation of single dyes. Our results showed that the crude laccase from *P. eryngii* was highly tolerant to SO_4_^2−^ salts and organic solvents, such as glycerol and ethylene glycol, during the decolorization of various types of dyes. Based on the degradation of single dyes, we further investigated the decolorization of mixed dyes, and the crude laccase from *P. eryngii* was found to be efficient in the decolorization of mixed dyes (mixtures of various types of dyes). The crude laccase from *P. eryngii* was also efficient in the repeated-batch decolorization of single dyes as well as two- and four-dye mixtures. The crude laccase preparation showed a high stability and reusability in repeated-batch decolorization. The crude laccase from *P. eryngii* was efficient in the detoxification of different types of single dyes and mixed dyes containing different types of dyes, and the phytotoxicity of decolorized dyes (single and mixed dyes) was significantly reduced. In conclusion, our results indicated that the crude laccase from *Pleurotus eryngii* had unique advantages and properties. These included efficient decolorization of a broad spectrum of dyes, efficient decolorization of mixed dyes (mixtures of various types of dyes), strong tolerance to different metal salts and organic solvents during dye degradation, repeated-batch decolorization of single and mixed dyes with high enzyme stability, and efficient detoxification of the phytotoxicity of single and mixed dyes. The crude laccase from *Pleurotus eryngii* offers great potential for the bioremediation and efficient treatment of pollutants containing single and mixed dyes with various molecular structures. Our results offer valuable insight and practical guidance for using crude laccase from *Pleurotus eryngii* in the efficient treatment of industrial wastewater containing dyes with diverse molecular structures.

## Figures and Tables

**Table 1:** The reaction rate constants and R^2^ values for different single dyes.

Types of Dye	Dye	The Reaction Rate Constants of Various Dyes (min^−1^)	R^2^ Values
Azo dye	New Coccine (NC)	$1.84\times 10^{-2}$	0.9938
	Reactive Black 5 (RB5)	$7.44\times 10^{-3}$	0.9582
	Acid Orange 7 (AO7)	$7.15\times 10^{-3}$	0.9711
Anthraquinone Dye	Remazol Brilliant Blue R (RBBR)	$6.05\times 10^{-2}$	0.9706
	Reactive Blue 4 (RB4)	$1.81\times 10^{-2}$	0.9827
Triphenylmethane Dye	Methyl Green (MG)	$7.23\times 10^{-4}$	0.9742
	Bromophenol Blue (BB)	$4.34\times 10^{-2}$	0.9565
	Acid Fuchsin (AF)	$3.87\times 10^{-4}$	0.9733
Indigo dye	Indigo Carmine (IC)	$3.05\times 10^{-3}$	0.9742

**Table 2:** The reaction rate constants and R^2^ values for different mixed dyes.

Mixed Dyes	Dye	The Reaction Rate Constants of Various Dyes (min^−1^)	R^2^ Values
NC + RB4	NC	$2.00\times 10^{-2}$	0.9516
	RB4	$1.28\times 10^{-2}$	0.9503
NC + RBBR	NC	$2.84\times 10^{-2}$	0.9808
	RBBR	$2.68\times 10^{-2}$	0.9539
NC + IC	NC	$7.58\times 10^{-4}$	0.9535
	IC	$4.62\times 10^{-3}$	0.9877
NC + MG	NC	$9.25\times 10^{-3}$	0.9848
	MG	$3.30\times 10^{-3}$	0.9505
RB4 + IC	RB4	$7.01\times 10^{-2}$	0.9866
	IC	$6.17\times 10^{-2}$	0.9815
MG + CR	MG	$1.48\times 10^{-2}$	0.9755
	CR	$1.14\times 10^{-2}$	0.9575
AF + BB	AF	$7.80\times 10^{-3}$	0.9707
	BB	$1.59\times 10^{-2}$	0.9792
CR + IC	CR	$1.00\times 10^{-2}$	0.9545
	IC	$1.55\times 10^{-1}$	0.9523
CR + RBBR	CR	$1.81\times 10^{-2}$	0.9529
	RBBR	$3.46\times 10^{-2}$	0.9652
MG + BB	MG	$5.78\times 10^{-3}$	0.9936
	BB	$3.34\times 10^{-3}$	0.9963
NC + RB5	NC	$6.04\times 10^{-3}$	0.9610
	RB5	$2.76\times 10^{-3}$	0.9536

**Table 3:** Full names, abbreviations, structures, and maximum absorption wavelengths of different dyes used in this study.

Type of Dye	Dye	Abbreviation	Structure	Maximum Absorption Wavelength (nm)
Azo Dye	New Coccine	NC	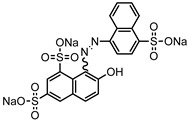	506
	Reactive Black 5	RB5	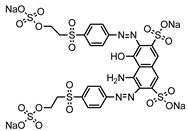	598
	Acid Orange 7	AO7	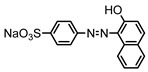	481
	Sunset Yellow	SY	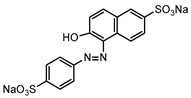	482
	Direct Red81	DR81	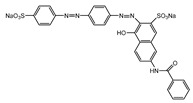	512
	Reactive Orange 16	RO16	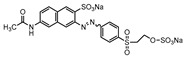	494
	Orange G	OG	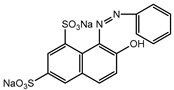	478
	Remazol Brilliant violet 5R	RBV5R	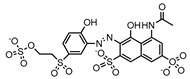	558
Anthraquinone Dye	Remazol Brilliant Blue R	RBBR	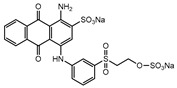	591
	Reactive Blue 4	RB4	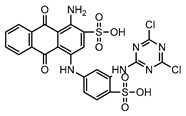	603
Triphenylmethane Dye	Methyl Green	MG	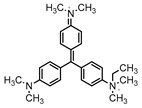	632
	Cresol Red	CR	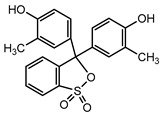	435
	Bromophenol Blue	BB	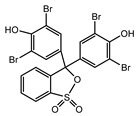	591
	Acid Fuchsin	AF	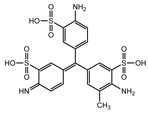	546
Indigo dye	Indigo Carmine	IC	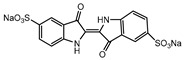	610

## Data Availability

The data presented in this study are available in article and Supplementary Materials.

## Supplementary Materials

The following supporting information can be downloaded at: https://www.mdpi.com/article/10.3390/molecules29030669/s1.

## References

[1] Patel N., Shahane S., Shivam Majumdar R., Mishra U. (2019). Mode of action, properties, production, and application of laccase: A review. Recent Pat. Biotechnol..

[2] Janusz G., Pawlik A., Świderska-Burek U., Polak J., Sulej J., Jarosz-Wilkołazka A., Paszczyński A. (2020). Laccase Properties, Physio-logical Functions, and Evolution. Int. J. Mol. Sci..

[3] Piontek K., Antorini M., Choinowski T. (2002). Crystal structure of a laccase from the Fungus *Trametes versicolor* at 1.90-Å resolution containing a full complement of coppers. J. Biol. Chem..

[4] Bhardwaj P., Kaur N., Selvaraj M., Ghramh H.A., Al-Shehri B.M., Singh G., Arya S.K., Bhatt K., Ghotekar S., Mani R. (2022). Laccase-assisted degradation of emerging recalcitrant compounds—A review. Bioresour. Technol..

[5] Kyomuhimbo H.D., Brink H.G. (2023). Applications and immobilization strategies of the copper-centred laccase enzyme; a review. Heliyon.

[6] Jeyabalan J., Veluchamy A., Priyan V V., Kumar A., Chandrasekar R., Narayanasamy S. (2023). A review on the laccase assisted decolourization of dyes: Recent trends and research progress. J. Taiwan Inst. Chem. Eng..

[7] Gao Y., Wang M., Shah K., Singh Kalra S., Rome L.H., Mahendra S. (2022). Decolorization and detoxification of synthetic dye compounds by laccase immobilized in vault nanoparticles. Bioresour. Technol..

[8] Yao C.Y., Xia W., Dou M.D., Du Y.Y., Wu J. (2022). Oxidative degradation of UV-irradiated polyethylene by laccase-mediator system. J. Hazard. Mater..

[9] Santo M., Weitsman R., Sivan A. (2013). The role of the copper-binding enzyme laccase in the biodegradation of polyethylene by the actinomycete *Rhodococcus ruber*. Int. Biodeterior. Biodegrad..

[10] Zhang J.B., Liu X.P., Xu Z.Q., Chen H., Yang Y.X. (2008). Degradation of chlorophenols catalyzed by laccase. Int. Biodeterior. Biodegrad..

[11] Chen J.H., Liu J.S., Chen B.X., Yang F., Li B.X., Li H.N., Jiang Z.B., Song H.T. (2023). Effective biodegradation of chlorophenols, sulfonamides, and their mixtures by bacterial laccase immobilized on chitin. Ecotoxicol. Environ. Saf..

[12] Xu P., Du H., Peng X., Tang Y., Zhou Y., Chen X., Fei J., Meng Y., Yuan L. (2020). Degradation of several polycyclic aromatic hydrocarbons by laccase in reverse micelle system. Sci. Total Environ..

[13] Hadibarata T., Yuniarto A. (2020). Biodegradation of polycyclic aromatic hydrocarbons by high-laccase basidiomycetes fungi isolated from tropical forest of Borneo. Biocatal. Agric. Biotechnol..

[14] Das A., Singh J., Yogalakshmi K.N. (2017). Laccase immobilized magnetic iron nanoparticles: Fabrication and its performance evaluation in chlorpyrifos degradation. Int. Biodeterior. Biodegrad..

[15] Rudakiya D.M., Patel D.H., Gupte A. (2020). Exploiting the potential of metal and solvent tolerant laccase from *Tricholoma giganteum* AGDR1 for the removal of pesticides. Int. J. Biol. Macromol..

[16] Dlamini M.L., Lesaoana M., Kotze I., Richards H. (2023). An oxidoreductase enzyme, fungal laccase immobilized on zeolitic imidazolate frameworks for the biocatalytic degradation of an endocrine-disrupting chemical, dimethyl phthalate. J. Environ. Chem. Eng..

[17] Barrios-Estrada C., de Jesús M.R.A., Gutiérrez M.B.D., Iqbal H.M.N., Kannan S., Parra-Saldívar R. (2018). Emergent contaminants: Endocrine disruptors and their laccase-assisted degradation—A review. Sci. Total Environ..

[18] Liu Y.Q., Maulidiany N., Zeng P., Heo S. (2021). Decolourization of azo, anthraquinone and triphenylmethane dyes using aerobic granules: Acclimatization and long-term stability. Chemosphere.

[19] Yang X.Q., Zhao X.X., Liu C.Y., Zheng Y., Qian S.J. (2009). Decolorization of azo, triphenylmethane and anthraquinone dyes by a newly isolated *Trametes* sp. SQ01 and its laccase. Process Biochem..

[20] Soares G.M.B., Amorim M.T.P., Hrdina R., Costa-Ferreira M. (2002). Studies on the biotransformation of novel diazo dyes by laccase. Process Biochem..

[21] Toker S.K., Evlat H., Koçyïğït A. (2021). Screening of newly isolated marine-derived fungi for their laccase production and decolorization of different dye types. Reg. Stud. Mar. Sci..

[22] Tomar T., Kahandawala N., Kaur J., Thounaojam L., Choudhary I., Bera S. (2023). Bioremediation of synthetic dyes from wastewater by using microbial nanocomposites: An emerging field for water pollution management. Biocatal. Agric. Biotechnol..

[23] Qurrat-ul-Ain, Khurshid S., Gul Z., Khatoon J., Shah M.R., Hamid I., Khan I.A.T., Aslam F. (2020). Anionic azo dyes removal from water using amine-functionalized cobalt–iron oxide nanoparticles: A comparative time-dependent study and structural optimization towards the removal mechanism. RSC Adv..

[24] Vats S., Srivastava S., Maurya N., Saxena S., Mudgil B., Yadav S., Chandra R. (2022). Chapter 8—Advances in Dye Contamination: Health Hazards, Biodegradation, and Bioremediation. Advances in Pollution Research, Biological Approaches to Controlling Pollutants.

[25] Herath I.S., Udayanga D., Jayasanka D.J., Hewawasam C. (2024). Textile dye decolorization by white rot fungi—A review. Bioresour. Technol. Rep..

[26] Hashemi S.H., Kaykhaii M. (2022). Azo dyes: Sources, occurrence, toxicity, sampling, analysis, and their removal methods. Emerg. Freshw. Pollut..

[27] Chung K.T. (2016). Azo dyes and human health: A review. J. Environ. Sci. Health Part C Environ. Carcinog. Ecotoxicol. Rev..

[28] Zhou Q., Zhao Y.C. (2005). Health Impacts of Typical Dyes and Pigments. J. Environ. Health.

[29] Uddin F. (2021). Environmental hazard in textile dyeing wastewater from local textile industry. Cellulose.

[30] Kumar V., Pallavi P., Kumar Sen S., Raut S. (2024). Harnessing the potential of white rot fungi and ligninolytic enzymes for efficient textile dye degradation: A comprehensive review. Water. Environ. Res..

[31] Zeng X.K., Cai Y.J., Liao X.R., Zeng X.L., Luo S.P., Zhang D.B. (2012). Anthraquinone dye assisted the decolorization of azo dyes by a novel *Trametes trogii* laccase. Process Biochem..

[32] Pan F. (2007). Decoloration of Printing and Dyeing Wastewater by Crude Enzyme of White-rot Fungus. J. Anhui Agric. Sci..

[33] Yanto D.H.Y., Anita S.H., Solihat N.N. (2022). Enzymatic degradation and metabolic pathway of acid blue 129 dye by crude laccase from newly isolated *Trametes hirsuta* EDN 082. Biocatal. Biotransformation.

[34] Couto S.R. (2007). Decolonization of industrial azo dyes by crude laccase from *Trametes Hirsuta*. J. Hazard. Mater..

[35] Jasińska A., Soboń A., Góralczyk-Bińkowska A., Długoński J. (2019). Analysis of decolorization potential of *Myrothecium roridum* in the light of its secretome and toxicological studies. Environ. Sci. Pollut. Res..

[36] Zeng X.K., Cai Y.J., Liao X.R., Zeng X.L., Li W.X., Zhang D.B. (2011). Decolorization of synthetic dyes by crude laccase from a newly isolated *Trametes trogii* strain cultivated on solid agro-industrial residue. J. Hazard. Mater..

[37] Hou H.M., Zhou J.T., Wang J., Du C.H., Yan B. (2004). Enhancement of laccase production by *Pleurotus ostreatus* and its use for the decolorization of anthraquinone dye. Process Biochem..

[38] Wong Y., Yu J. (1999). Laccase-catalyzed decolorization of synthetic dyes. Water Res..

[39] Yadav A., Yadav P., Singh A.K., Kumar V., Sonawane V.C., Markandeya, Bharagava R.N., Raj A. (2021). Decolourisation of textile dye by laccase: Process evaluation and assessment of its degradation bioproducts. Bioresour. Technol..

[40] Osma J.F., Herrera J.L.T., Couto S.R. (2007). Banana skin: A novel waste for laccase production by *Trametes pubescens* under solid-state conditions. Application to synthetic dye decolouration. Dye. Pigment..

[41] Grassi E., Scodeller P., Filiel N., Carballo R., Levin L. (2011). Potential of *Trametes trogii* culture fluids and its purified laccase for the decolorization of different types of recalcitrant dyes without the addition of redox mediators. Int. Biodeterior. Biodegrad..

[42] Li J.H. (2019). Purification and Characterization of Three Laccases from Pycnoporus sanguineus MX5 and Their Decolorization of Industrial Dyes.

[43] Hadibarata T., Yusoff A.R.M., Aris A., Salmiati Hidayat S.T., Kristanti R.A. (2012). Decolorization of Azo, Triphenylmethane and Anthraquinone Dyes by Laccase of a Newly Isolated *Armillaria* sp. F022. Water Air Soil Pollut..

[44] Núria C., Teodor P., Teresa V., Glòria C., Montserrat S. (2009). Metabolites from the biodegradation of triphenylmethane dyes by *Trametes versicolor* or laccase. Chemosphere.

[45] Hu M.R., Chao Y.P., Zhang G.Q., Xue Z.Q., Qian S. (2009). Laccase-mediator system in the decolorization of different types of recalcitrant dyes. J. Ind. Microbiol. Biotechnol..

[46] Collins P.J., Dobson A.D.W. (1997). Regulation of laccase gene transcription in *Trametes versicolor*. Appl. Environ. Microbiol.

[47] Enayatzamir K., Alikhani H.A., Couto S.R. (2009). Simultaneous production of laccase and decolouration of the diazo dye Reactive Black 5 in a fixed-bed bioreactor. J. Hazard. Mater..

[48] Darvishi F., Moradi M., Jolivalt C., Madzak C. (2018). Laccase production from sucrose by recombinant *Yarrowia lipolytica* and its application to decolorization of environmental pollutant dyes. Ecotoxicol. Environ. Saf..

[49] Domínguez A., Couto S.R., Sanroman M.A. (2005). Dye decolorization by *Trametes hirsuta* immobilized into alginate beads. World J. Microbiol. Biotechnol..

[50] Kandelbauer A., Maute O., Kessler R.W., Erlacher A., Gübitz G.M. (2004). Study of dye decolorization in an immobilized laccase enzyme-reactor using online spectroscopy. Biotechnol Bioeng..

[51] Ramírez-Montoya L.A., Hernández-Montoya V., Montes-Morán M.A., Jáuregui-Rincón J., Cervante F.J. (2015). Decolorization of dyes with different molecular properties using free and immobilized laccases from *Trametes versicolor*. J. Mol. Liq..

[52] Gomes E., Aguiar A.P., Carvalho C.C., Bonfá M.R.B., Silva R.D., Boscolo M. (2009). Ligninases production by *Basidiomycetes* strains on lignocellulosic agricultural residues and their application in the decolorization of synthetic dyes. Braz. J. Microbiol..

[53] Cardoso B.K., Linde G.A., Colauto N.B., do Valle J.S. (2018). *Panus strigellus* laccase decolorizes anthraquinone, azo, and triphenylmethane dyes. Biocatal. Agric. Biotechnol..

[54] Pasti-Grigsby M.B., Paszczynski A., Goszczynski S., Crawford D.L., Crawford R.L. (1992). Influence of aromatic substitution patterns on azo dye degradability by *Streptomyces* spp. and *Phanerochaete chrysosporium*. Appl. Environ. Microbiol..

[55] Azmi W., Sani R.K., Banerjee U.C. (1998). Biodegradation of triphenylmethane dyes. Enzym. Microb. Technol..

[56] Wang X.W., Zhan H.Y., He W. (2003). Effect of metal ions on laccase activity. China Pulp Pap. Ind..

[57] Couto S.R., Sanromán M., Gübitz G.M. (2005). Influence of redox mediators and metal ions on synthetic acid dye decolourization by crude laccase from *Trametes hirsuta*. Chemosphere.

[58] Zilly A., da Silva J.C.M., Bracht A., de Souza C.G.M., Carvajal A.E., Koehnlein E.A., Peralta R.M. (2011). Influence of NaCl and Na_2_SO_4_ on the kinetics and dye decolorization ability of crude laccase from *Ganoderma lucidum*. Int. Biodeterior. Biodegrad..

[59] Gao E.L., Zhong W.J., Fu X.L., Chen F.S., Ye Z.G. (2012). Decolorization on Indigo Dyeing Wastewater by Laccase from *Coriolus versicolor*. J. Anhui Agric. Sci..

[60] Wang Y.Q., Xie X.H., Zheng X.L., Zhang Q.Y., Xu K.X., Liu J.S. (2019). Advances in research on activators promoting microbial degradation of dyes. Chem. Ind. Eng. Prog..

[61] Forootanfar H., Moezzi A., Aghaie M.K., Mahmoudjanlou Y., Ameri A., Niknejad F., Faramarzi M.A. (2012). Synthetic dye decolorization by three sources of fungal laccase. Iran. J. Environ. Health Sci Eng..

[62] Zille A., Górnacka B., Rehorek A., Cavaco-Paulo A. (2005). Degradation of azo dyes by *Trametes villosa* laccase over long periods of oxidative conditions. Appl. Environ. Microbiol..

