# Supplementary material for: Effective Decolorization and Detoxification of Single and Mixed Dyes with Crude Laccase Preparation from a White-Rot Fungus Strain Pleurotus eryngii
Source: Molecules. 2024 Jan 31;29(3):669. doi: 10.3390/molecules29030669 (PMC10856677; doi:10.3390/molecules29030669)
Supplement: Supplementary file 1 [file molecules-29-00669-s001.zip › molecules-2811403-supplementary.pdf]

Supplementary Materials

Article

Effective Decolorization and Detoxification of Single and Mixed Dyes with Crude Laccase Preparation from a White-Rot Fungus Strain *Pleurotus eryngii*

Mingrui Ge, Wei Deng, Ziyi Wang, Chenwen Weng and Yang Yang *

School of Life Sciences, Hubei Key Laboratory of Genetic Regulation and Integrative Biology, Central China Normal University, Wuhan, 430079, China
* Correspondence author: yangy@mail.ccnu.edu.cn

Table S1. The values of the molar absorption coefficient（ε）of different dyes.

Types of dye	Dye	Molar Absorption Coefficient（ε）
Azo dye	New Coccine (NC)	3.94×10^{-2}
	Reactive Black 5 (RB5)	2.14×10^{-2}
	Acid Orange 7 (AO7)	4.63×10^{-2}
Anthraquinone Dye	Remazol Brilliant Blue R (RBBR)	7.00×10^{-3}
	Reactive Blue 4 (RB4)	5.10×10^{-3}
Triphenylmethane Dye	Methyl Green (MG)	5.24×10^{-2}
	Bromophenol Blue (BB)	1.02×10^{-1}
	Acid Fuchsin (AF)	3.41×10^{-2}
Indigo dye	Indigo Carmine (IC)	2.73×10^{-2}

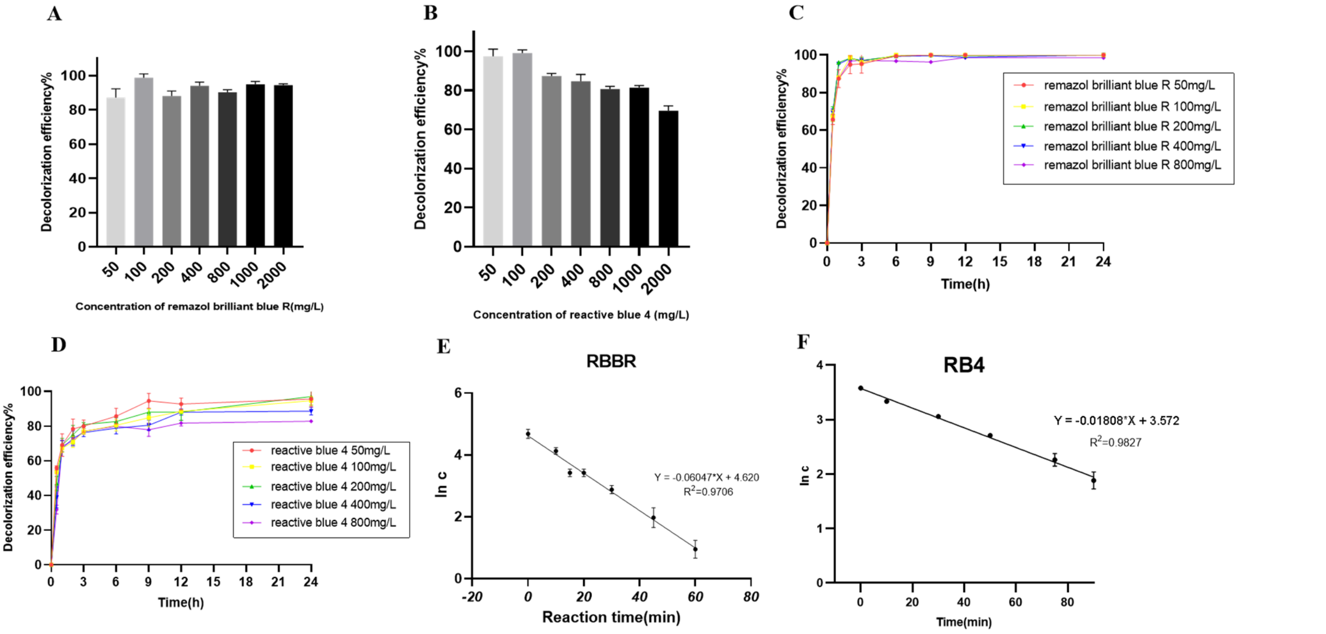

Figure S1. Decolorization of anthraquinone dyes with the crude laccase from *P. eryngii*. (A) Decolorization efficiencies of Remazol Brilliant Blue R at different concentrations. (B) Decolorization efficiencies of Reactive Blue 4 at different concentrations. (C) Time course of decolorization of Remazol Brilliant Blue R (final concentration was 50, 100, 200, 400, 800 mg/L). (D) Time course of decolorization of Reactive Blue 4 (final concentration was 50, 100, 200, 400, 800 mg/L). (E) The ln c-t curve of decolorization of the anthraquinone dye RBBR (100 mg/L). (F) The ln c-t curve of decolorization of the anthraquinone dye RB4 (100 mg/L). RBBR: Remazol Brilliant Blue R; RB4: Reactive Blue 4.

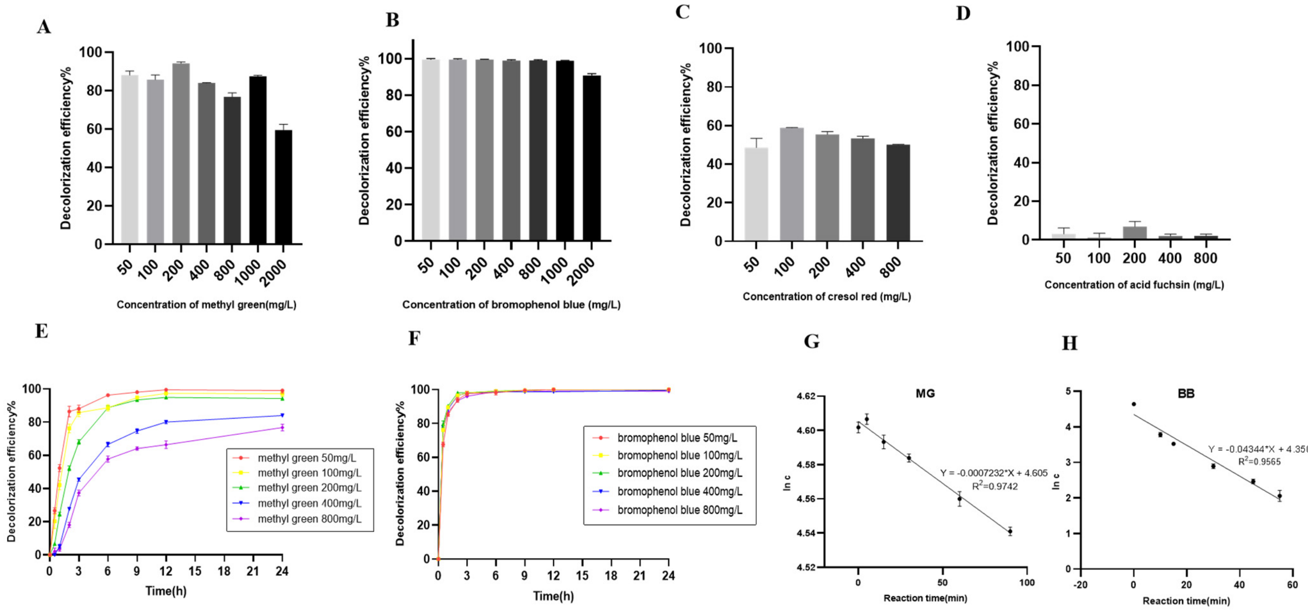

Figure S2. Decolorization of triphenylmethane dyes with the crude laccase from *P. eryngii*. (A) Decolorization efficiencies of Methyl Green at different concentrations. (B) Decolorization efficiencies of Bromophenol Blue at different concentrations. (C) Decolorization efficiencies of Cresol Red at different concentrations. (D) Decolorization efficiencies of Acid Fuchsin at different concentrations. (E) Time course of decolorization of Methyl Green (final concentration was 50, 100, 200, 400, 800 mg/L). (F) Time course of decolorization of Bromophenol Blue (final concentration was 50, 100, 200, 400, 800 mg/L). (G) The ln c-t curve of decolorization of the triphenylmethane dye MG (100 mg/L). (H) The ln c-t curve of decolorization of the triphenylmethane dye BB (100 mg/L). MG: Methyl Green; BB: Bromophenol Blue.

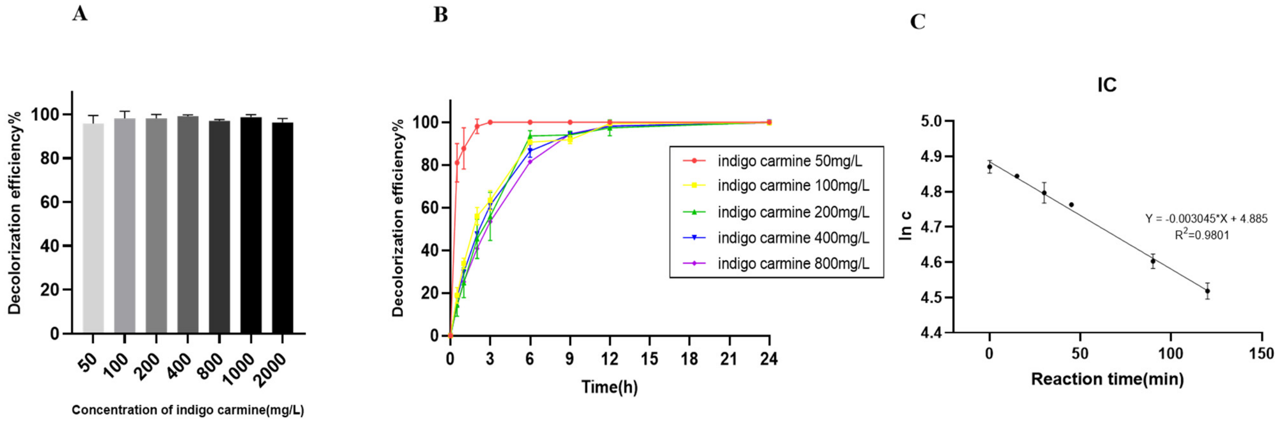

Figure S3. Decolorization of indigo dye with the crude laccase from *P. eryngii*. (A) Decolorization efficiencies of Indigo Carmine at different concentrations. (B) Time course of decolorization of Indigo Carmine (final concentration was 50, 100, 200, 400, 800 mg/L). (C) The ln c-t curve of decolorization of the indigo dye IC (100 mg/L). IC: Indigo Carmine.

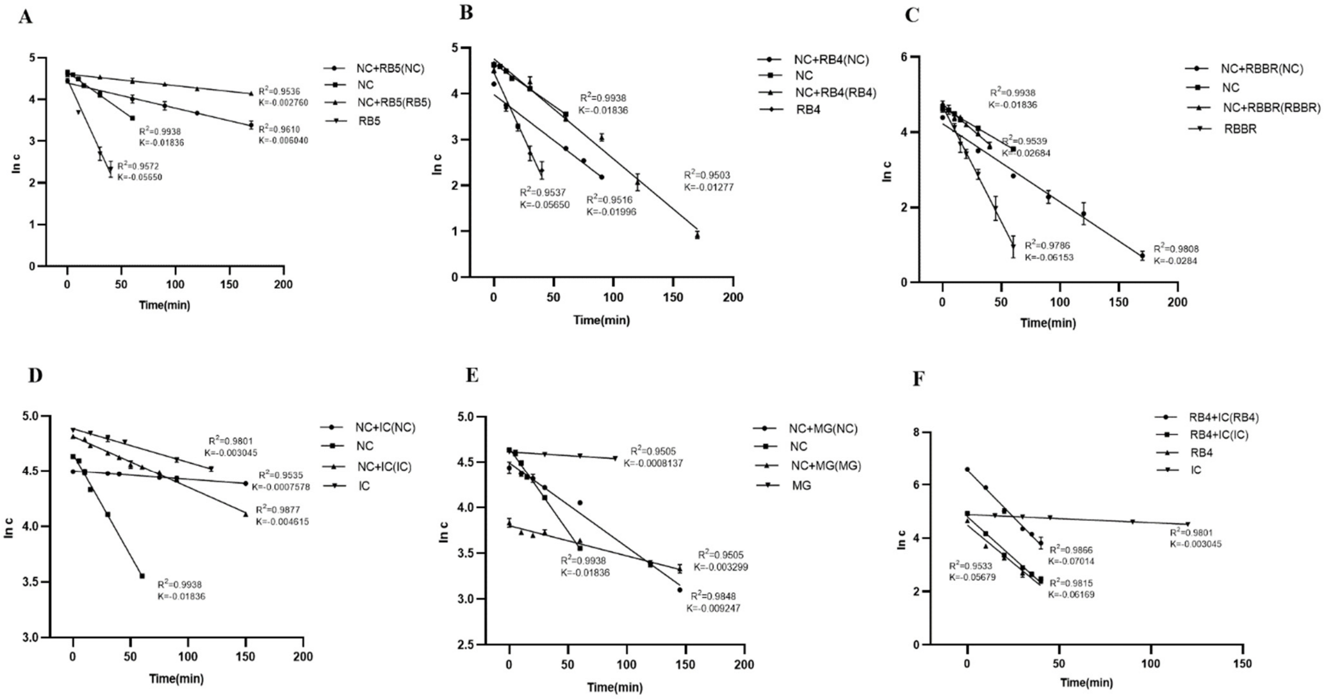

Figure S4. Decolorization kinetics of mixed dyes—the ln c-t curves for the single and mixed dyes. (A) Decolorization kinetics of azo + azo dyes. The ln c-t curves for the single (NC, RB5) and mixed dyes (NC+RB5). NC: New Coccine, RB5: Reactive Black 5. (B) Decolorization kinetics of azo + anthraquinone dyes. The ln c-t curves for the single (NC, RB4) and mixed dyes (NC+RB4). NC: New Coccine, RB4: Reactive Blue 4. (C) Decolorization kinetics of azo + anthraquinone dyes. The ln c-t curves for the single (NC, RBBR) and mixed dyes (NC+RBBR). NC: New Coccine, RBBR: Remazol Brilliant Blue R. (D) Decolorization kinetics of azo + indigo dyes. The ln c-t curves for the single (NC, IC) and mixed dyes (NC+IC). NC: New Coccine, IC: Indigo Carmine. (E) Decolorization kinetics of azo + triphenylmethane dyes. The ln c-t curves for the single (NC, MG) and mixed dyes (NC+MG). NC: New Coccine, MG: Methyl Green. (F) Decolorization kinetics of anthraquinone + indigo dyes. The ln c-t curves for the single (RB4, IC) and mixed dyes (RB4+IC). RB4: Reactive Blue 4, IC: Indigo Carmine.

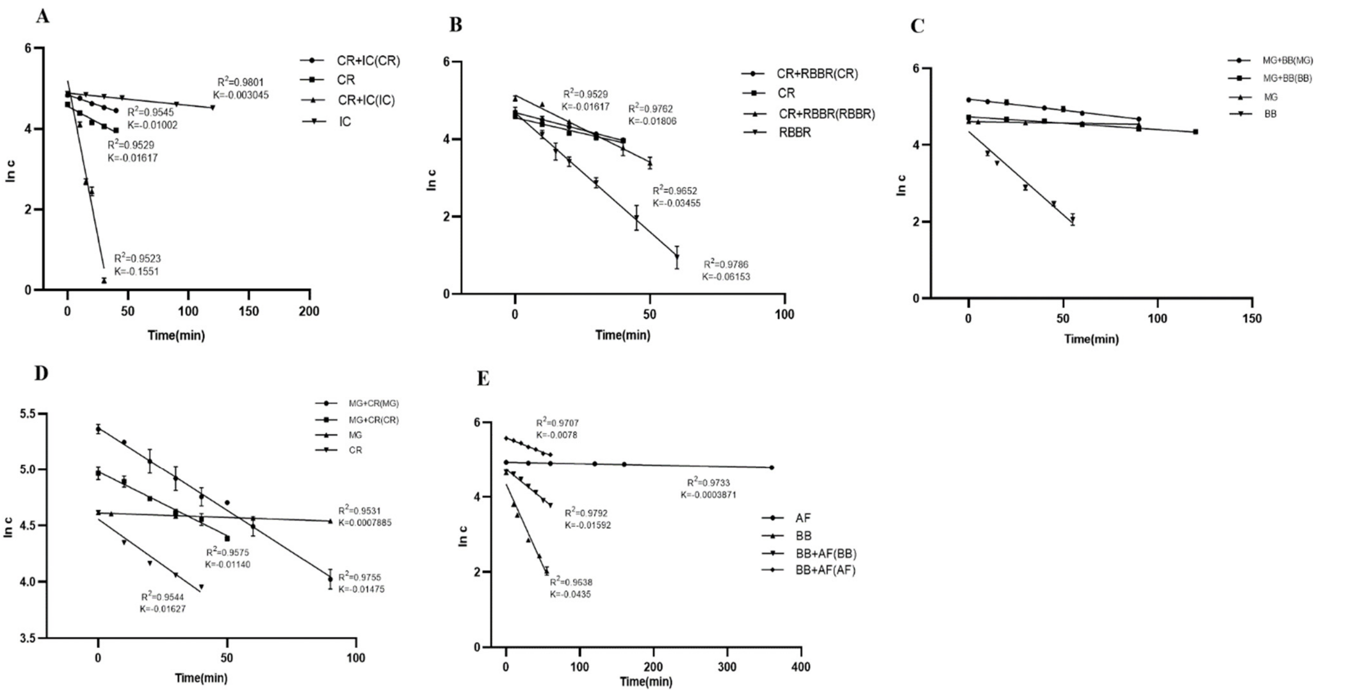

Figure S5. Decolorization kinetics of mixed dyes—the ln c-t curves for the single and mixed dyes. (A) Decolorization kinetics of indigo + triphenylmethane dyes. The ln c-t curves for the single (CR, IC) and mixed dyes (CR+IC). CR: Cresol Red, IC: Indigo Carmine. (B) Decolorization kinetics of anthraquinone + triphenylmethane dyes. The ln c-t curves for the single (CR, RBBR) and mixed dyes (CR+RBBR). CR: Cresol Red, RBBR: Remazol Brilliant Blue R. (C) Decolorization kinetics of triphenylmethane + triphenylmethane dyes. The ln c-t curves for the single (MG, BB) and mixed dyes (MG+BB). MG: Methyl Green, BB: Bromophenol Blue. (D) Decolorization kinetics of triphenylmethane + triphenylmethane dyes. The ln c-t curves for the single (MG, CR) and mixed dyes (MG+CR). MG: Methyl Green, CR: Cresol Red. (E) Decolorization kinetics of triphenylmethane + triphenylmethane dyes. The ln c-t curves for the single (BB, AF) and mixed dyes (BB+AF). BB: Bromophenol Blue, AF: Acid Fuchsin.

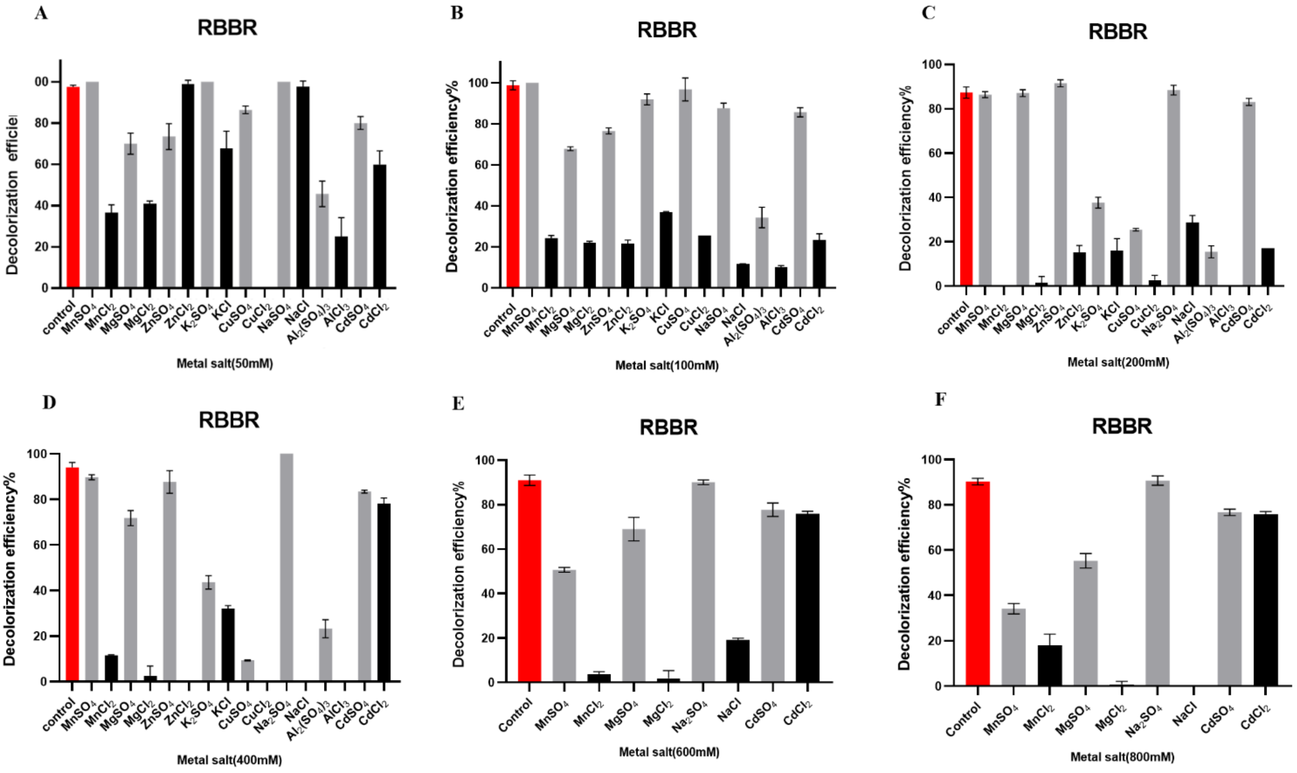

Figure S6. Effects of metal salts at different concentrations on the degradation of anthraquinone dye RBBR (Remazol Brilliant Blue R) with the crude laccase from *P. eryngii*. The metal salts used in this study were MnSO4, MnCl2, MgSO4, MgCl2, ZnSO4, ZnCl2, K2SO4, KCl, CuSO4, CuCl2, Na2SO4, NaCl, Al2(SO4)3, AlCl3, CdSO4, and CdCl2. The control group consisted of a 1 mL decolorization system without metal salts. (A) The final concentration of metal salt was 50 mM. (B) The final concentration of metal salt was 100 mM. (C) The final concentration of metal salt was 200 mM. (D) The final concentration of metal salt was 400 mM. (E) The final concentration of metal salt was 600 mM. (F) The final concentration of metal salt was 800 mM.

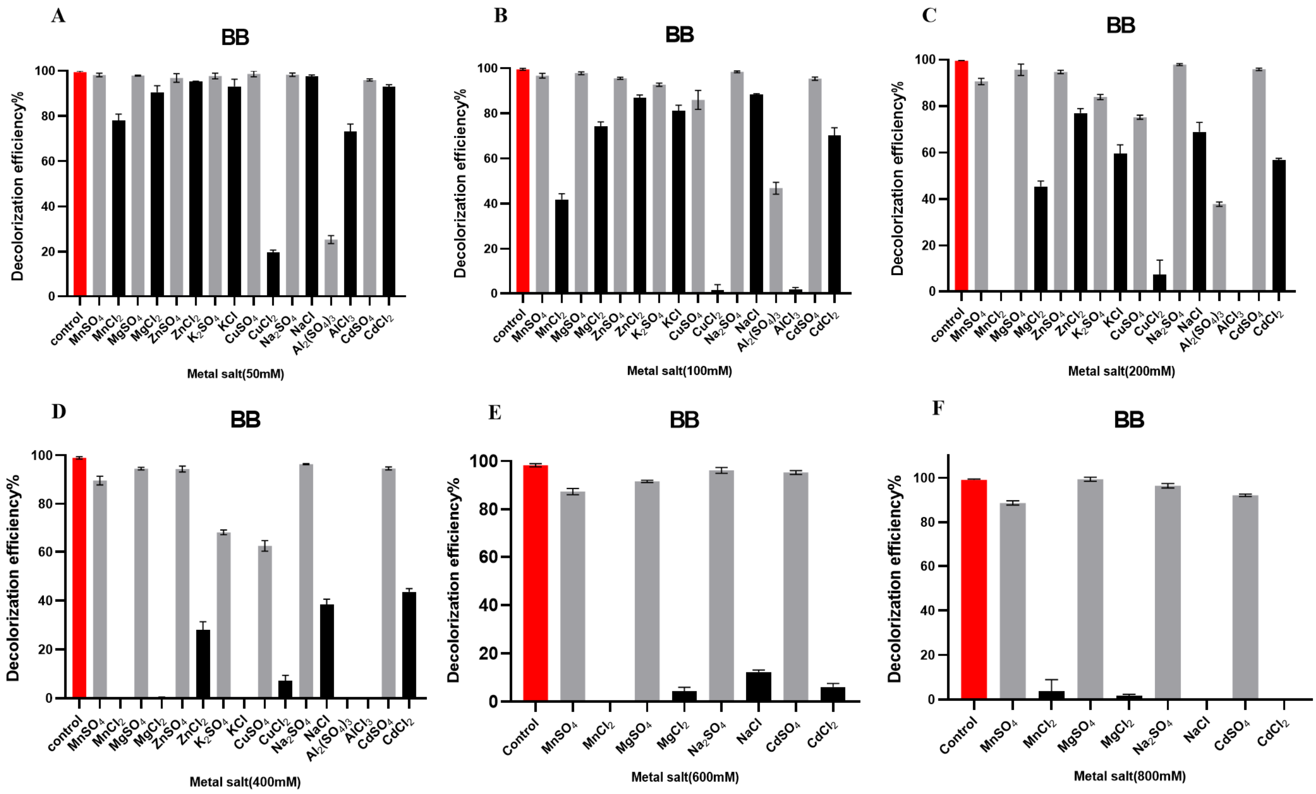

Figure S7. Effects of metal salts at different concentrations on the degradation of triphenylmethane dye BB (Bromophenol Blue) with the crude laccase from *P. eryngii*. The metal salts used in this study were MnSO4, MnCl2, MgSO4, MgCl2, ZnSO4, ZnCl2, K2SO4, KCl, CuSO4, CuCl2, Na2SO4, NaCl, Al2(SO4)3, AlCl3, CdSO4, and CdCl2. The control group consisted of a 1 mL decolorization system without metal salts. (A) The final concentration of metal salt was 50 mM. (B) The final concentration of metal salt was 100 mM. (C) The final concentration of metal salt was 200 mM. (D) The final concentration of metal salt was 400 mM. (E) The final concentration of metal salt was 600 mM. (F) The final concentration of metal salt was 800 mM.

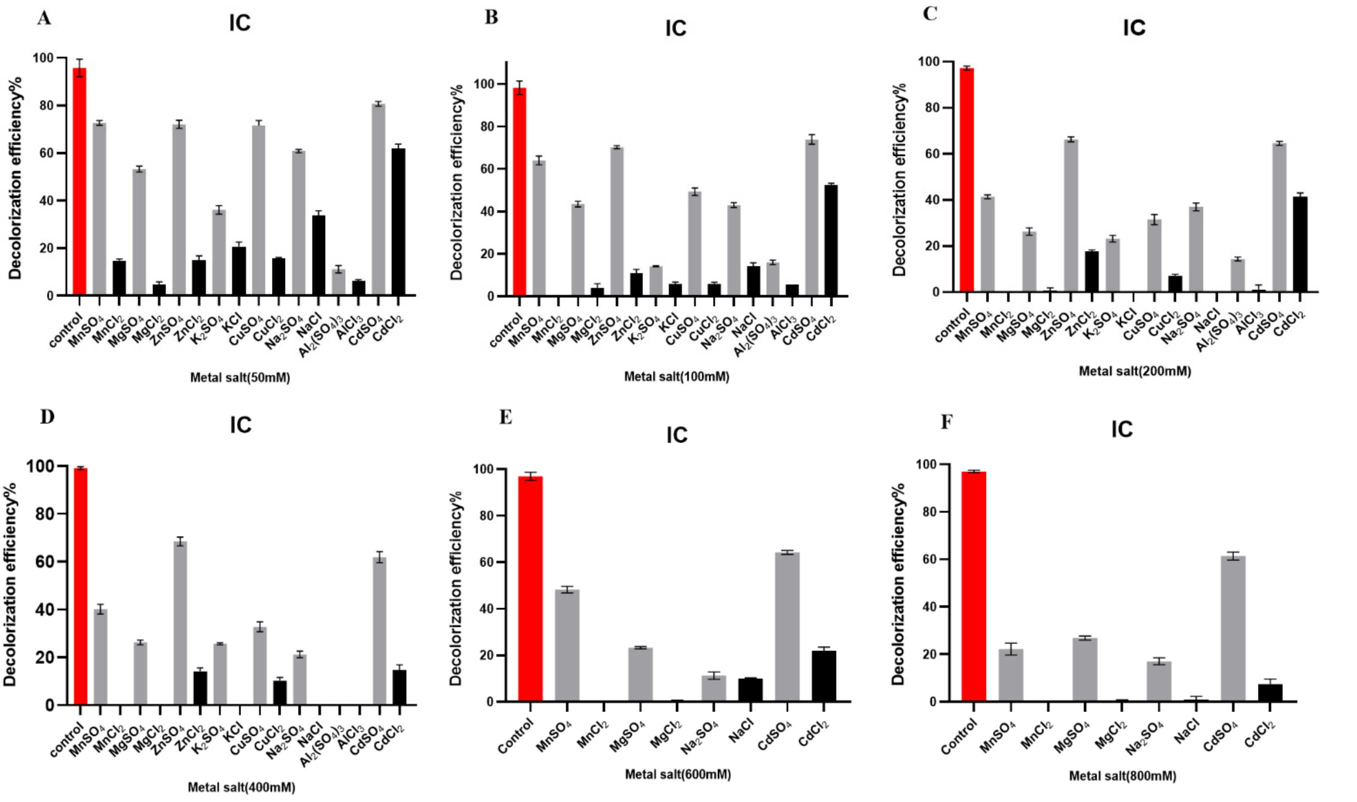

Figure S8. Effects of metal salts at different concentrations on the degradation of indigo dye IC (Indigo Carmine) with the crude laccase from *P. eryngii*. The metal salts used in this study were MnSO4, MnCl2, MgSO4, MgCl2, ZnSO4, ZnCl2, K2SO4, KCl, CuSO4, CuCl2, Na2SO4, NaCl, Al2(SO4)3, AlCl3, CdSO4, and CdCl2. The control group consisted of a 1 mL decolorization system without metal salts. (A) The final concentration of metal salt was 50 mM. (B) The final concentration of metal salt was 100 mM. (C) The final concentration of metal salt was 200 mM. (D) The final concentration of metal salt was 400 mM. (E) The final concentration of metal salt was 600 mM. (F) The final concentration of metal salt was 800 mM.

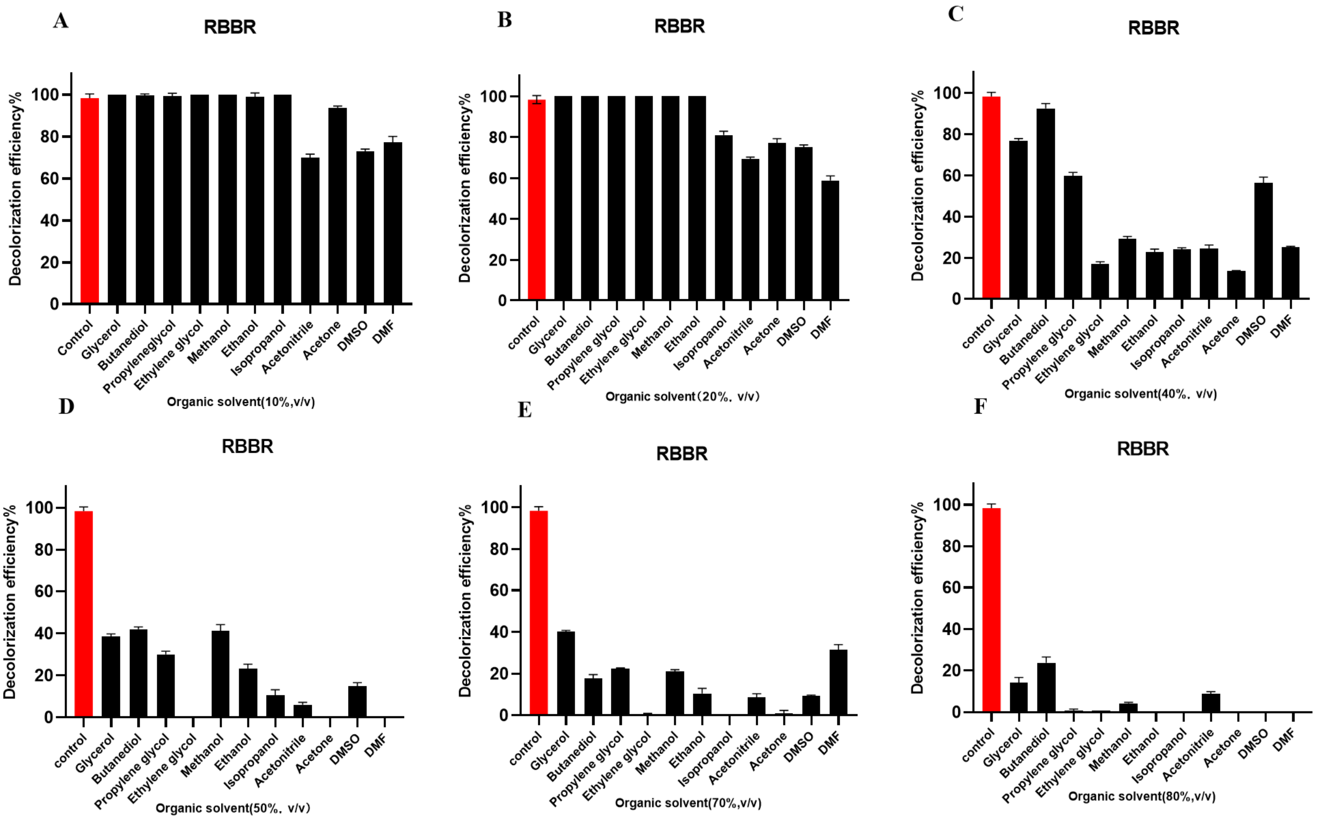

Figure S9 Effects of organic solvents at different concentrations on the degradation of anthraquinone dye RBBR (Remazol Brilliant Blue R) with the crude laccase from *P. eryngii*. The organic solvents used in this study were glycerol, butanediol, propylene glycol, ethylene glycol, methanol, ethanol, isopropanol, acetonitrile, acetone, DMSO, and DMF. The control group consisted of a 1 mL decolorization system without metal salts. (A) The final concentration of organic solvent was 10% (v/v). (B) The final concentration of organic solvent was 20% (v/v). (C) The final concentration of organic solvent was 40% (v/v). (D) The final concentration of organic solvent was 50% (v/v). (E) The final concentration of organic solvent was 70% (v/v). (F) The final concentration of organic solvent was 80% (v/v).

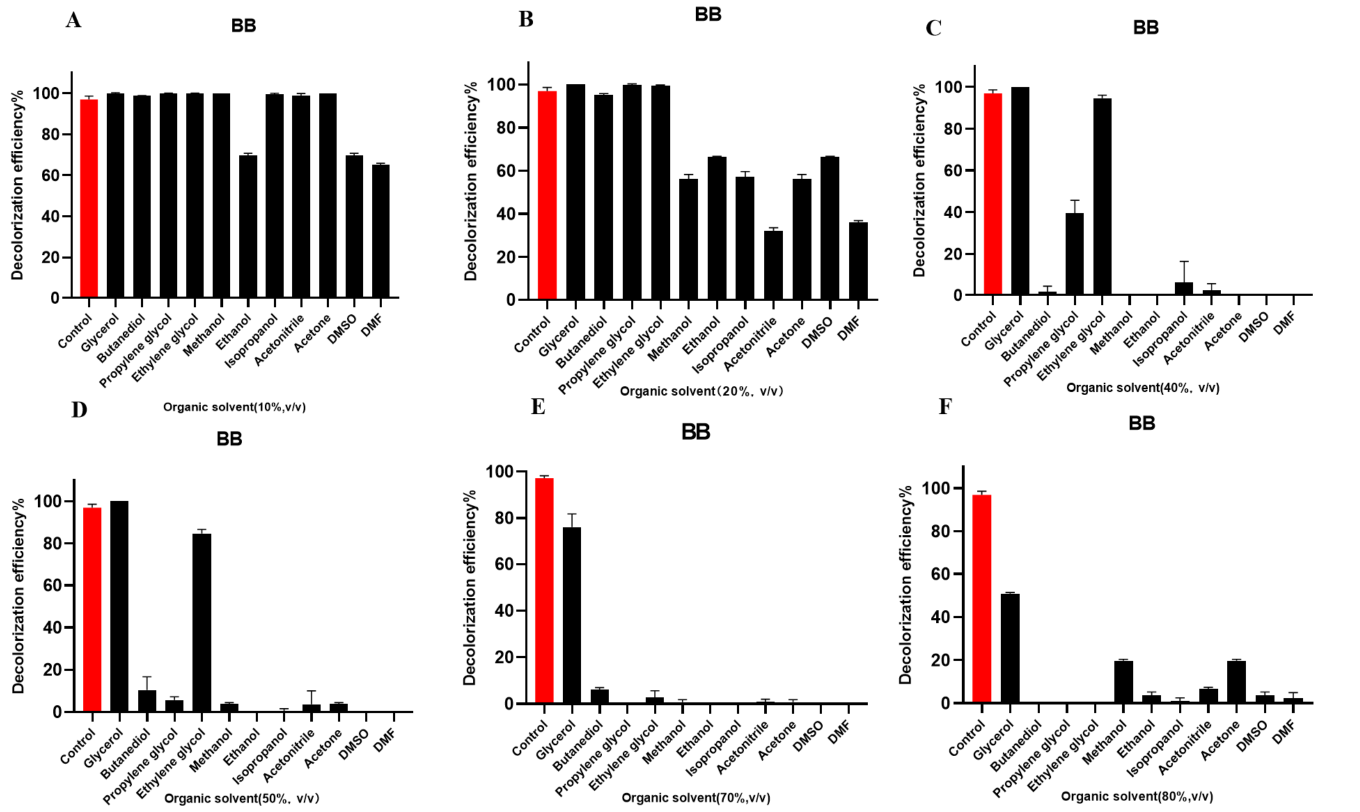

Figure S10. Effects of organic solvents at different concentrations on the degradation of triphenylmethane dye BB (Bromophenol Blue) with the crude laccase from *P. eryngii*. The organic solvents used in this study were glycerol, butanediol, propylene glycol, ethylene glycol, methanol, ethanol, isopropanol, acetonitrile, acetone, DMSO, and DMF. The control group consisted of a 1 mL decolorization system without metal salts. (A) The final concentration of organic solvent was 10% (v/v). (B) The final concentration of organic solvent was 20% (v/v). (C) The final concentration of organic solvent was 40% (v/v). (D) The final concentration of organic solvent was 50% (v/v). (E) The final concentration of organic solvent was 70% (v/v). (F) The final concentration of organic solvent was 80% (v/v).

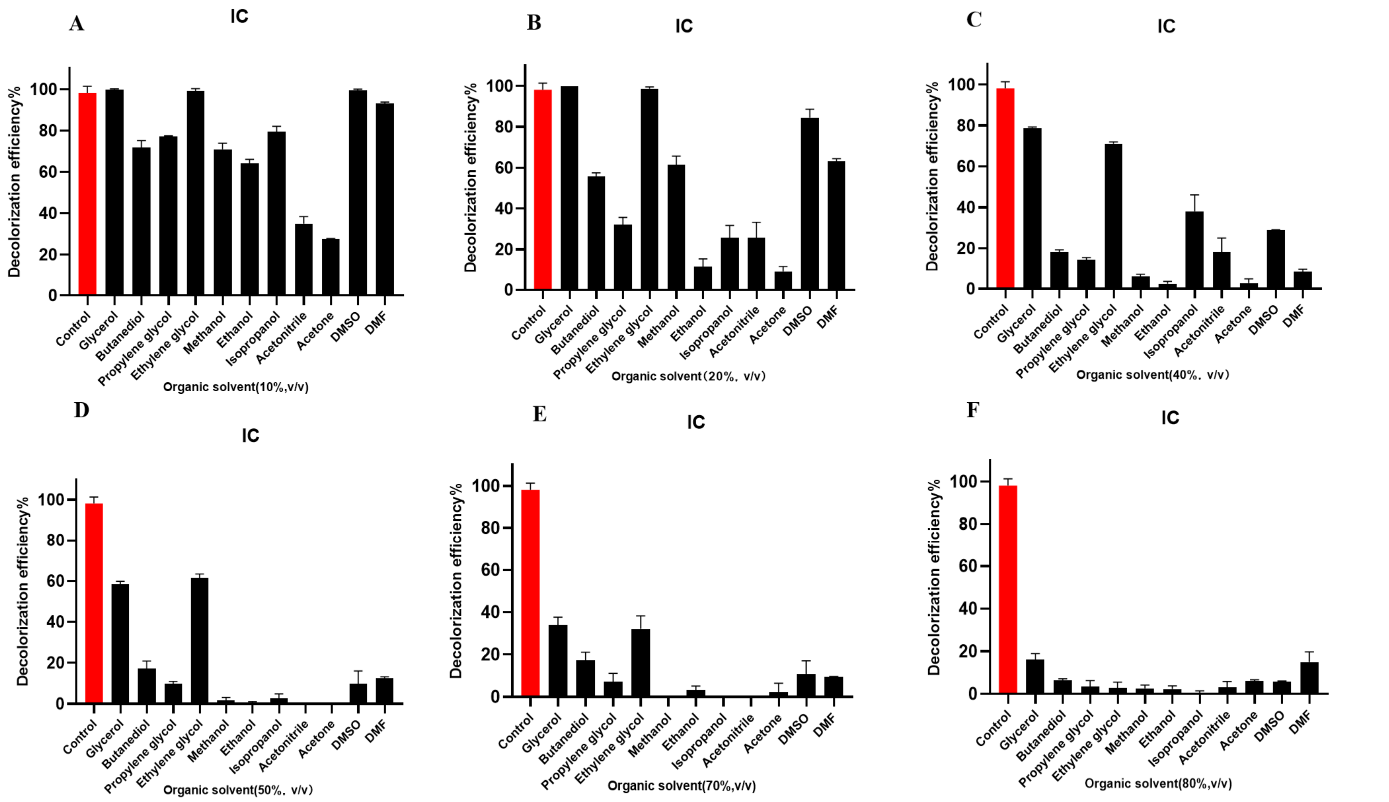

Figure S11 Effects of organic solvents at different concentrations on the degradation of indigo dye IC (Indigo Carmine) with the crude laccase from *P. eryngii*. The organic solvents used in this study were glycerol, butanediol, propylene glycol, ethylene glycol, methanol, ethanol, isopropanol, acetonitrile, acetone, DMSO, and DMF. The control group consisted of a 1 mL decolorization system without metal salts. (A) The final concentration of organic solvent was 10% (v/v). (B) The final concentration of organic solvent was 20% (v/v). (C) The final concentration of organic solvent was 40% (v/v). (D) The final concentration of organic solvent was 50% (v/v). (E) The final concentration of organic solvent was 70% (v/v). (F) The final concentration of organic solvent was 80% (v/v).

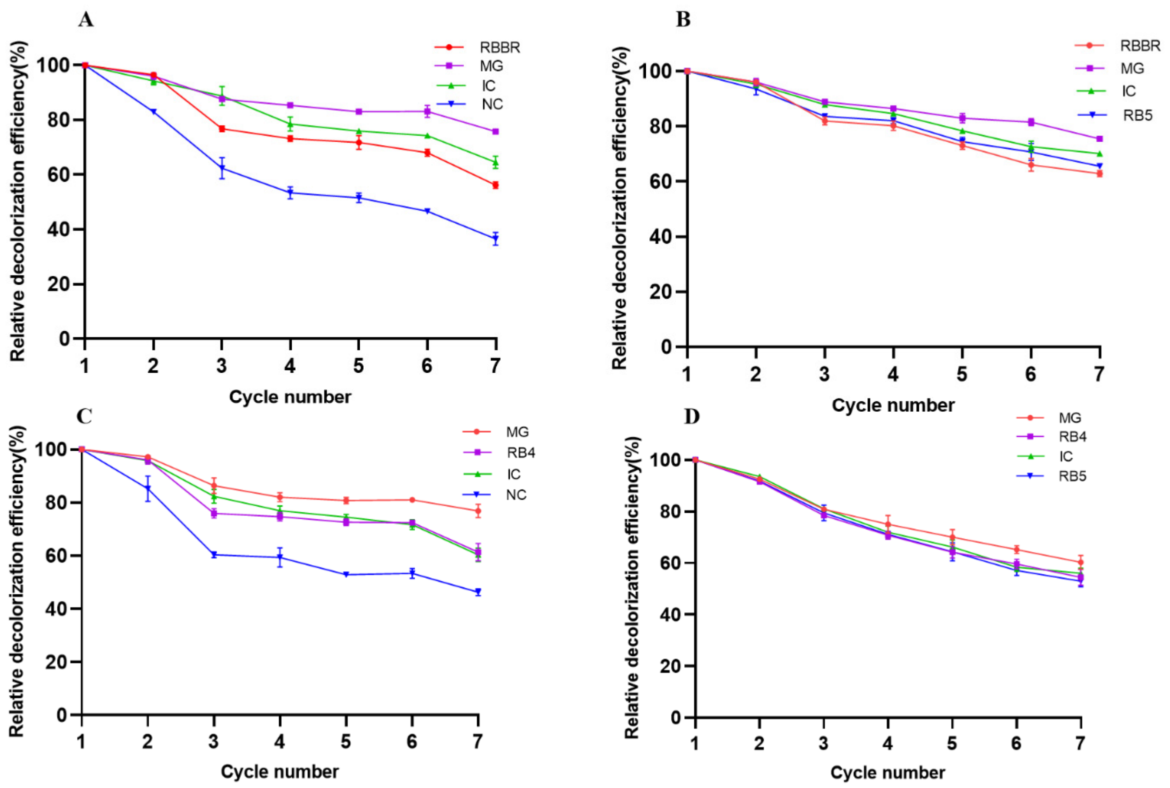

Figure S12. Repeated-batch decolorization of four-dye mixtures with the crude laccase from *P. eryngii*. (A) Repeated-batch decolorization of azo + anthraquinone + triphenylmethane + indigo dyes (NC+RBBR+MG+IC). (B) Repeated-batch decolorization of azo + anthraquinone + triphenylmethane + indigo dyes (RB5+RBBR+MG+IC). (C) Repeated-batch decolorization of azo + anthraquinone + triphenylmethane + indigo dyes (NC+RB4+MG+IC). (D) Repeated-batch decolorization of azo + anthraquinone + triphenylmethane + indigo dyes (RB5+RB4+MG+IC).

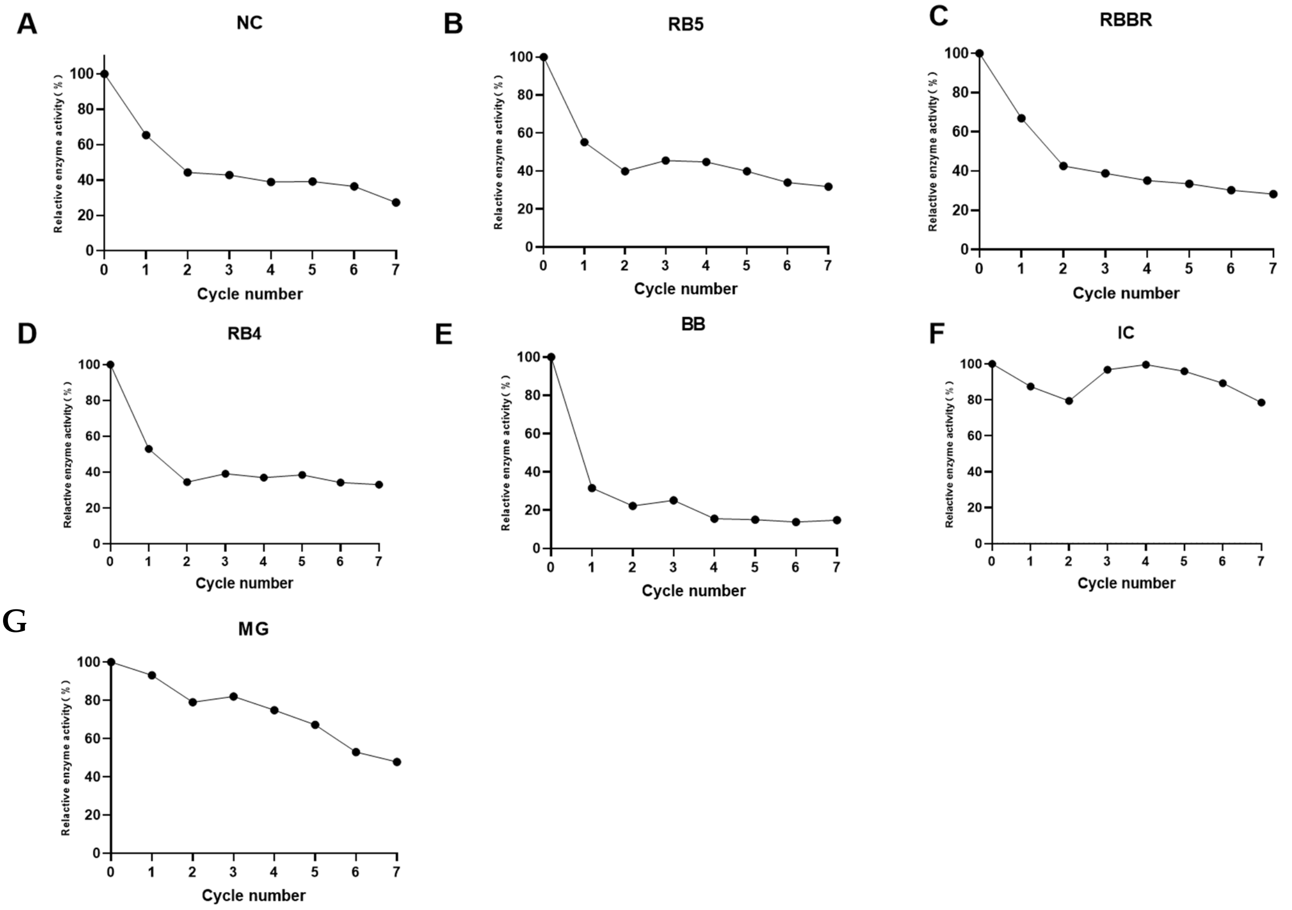

Figure S13. The change in laccase activity during the repeated-batch decolorization of single dyes. The initial laccase activity of crude laccase (cycle 0) was set to 100%, and the relative laccase activity of each cycle (%) was calculated. (A) NC. (B) RB5. (C) RBBR. (D) RB4. (E) BB. (F) IC. (G) MG.

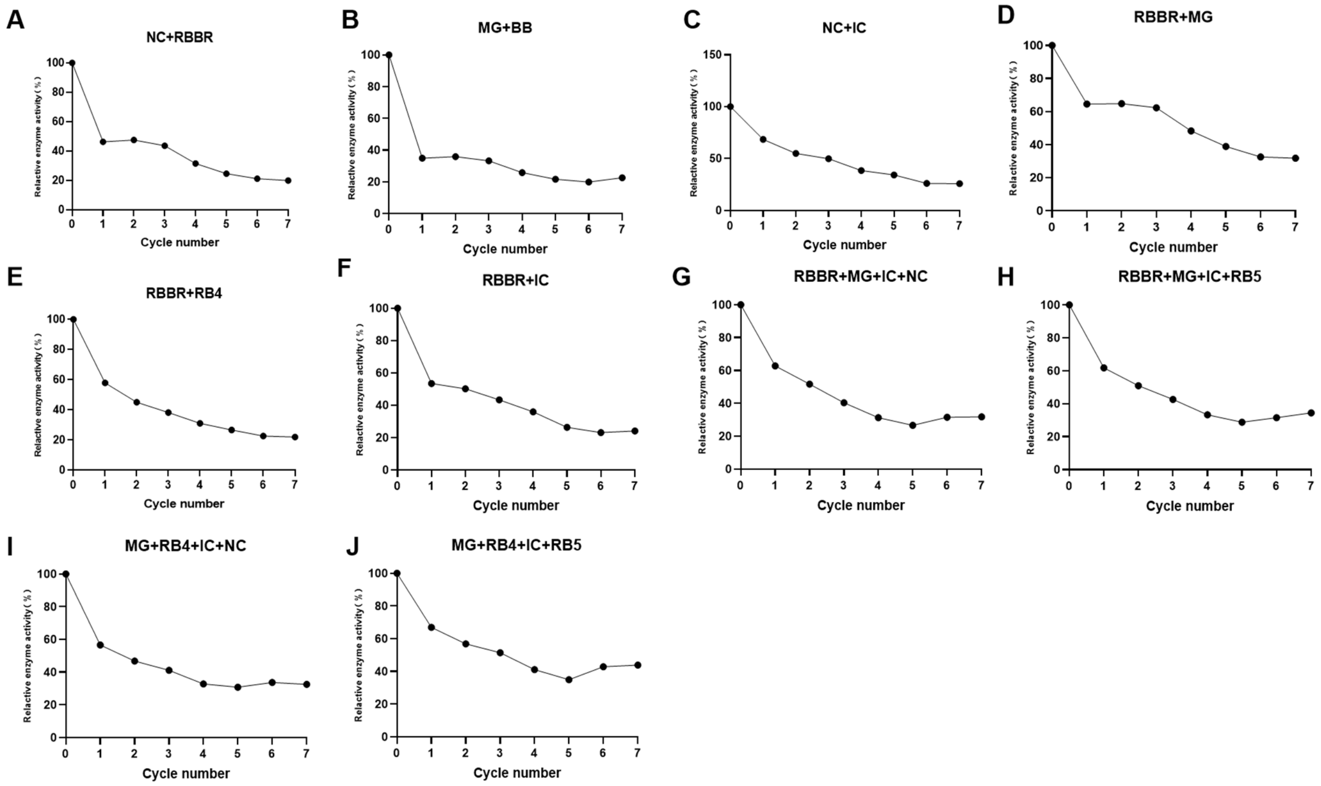

Figure S14. The change in laccase activity during the repeated-batch decolorization of two-dye mixtures and four-dye mixtures. The initial laccase activity of crude laccase (cycle 0) was set to 100%, and the relative laccase activity of each cycle (%) was calculated. (A)-(F) two-dye mixtures. (G)-(J) four-dye mixtures.
